# RedCom: A strategy for reduced metabolic modeling of complex microbial communities and its application for analyzing experimental datasets from anaerobic digestion

**DOI:** 10.1371/journal.pcbi.1006759

**Published:** 2019-02-01

**Authors:** Sabine Koch, Fabian Kohrs, Patrick Lahmann, Thomas Bissinger, Stefan Wendschuh, Dirk Benndorf, Udo Reichl, Steffen Klamt

**Affiliations:** 1 Max Planck Institute for Dynamics of Complex Technical Systems, Magdeburg, Germany; 2 Otto-von-Guericke University Magdeburg, Faculty for Process and Systems Engineering, Magdeburg, Germany; VU University, NETHERLANDS

## Abstract

Constraint-based modeling (CBM) is increasingly used to analyze the metabolism of complex microbial communities involved in ecology, biomedicine, and various biotechnological processes. While CBM is an established framework for studying the metabolism of single species with linear stoichiometric models, CBM of communities with balanced growth is more complicated, not only due to the larger size of the multi-species metabolic network but also because of the bilinear nature of the resulting community models. Moreover, the solution space of these community models often contains biologically unrealistic solutions, which, even with model linearization and under application of certain objective functions, cannot easily be excluded. Here we present RedCom, a new approach to build reduced community models in which the metabolisms of the participating organisms are represented by net conversions computed from the respective single-species networks. By discarding (single-species) net conversions that violate a minimality criterion in the exchange fluxes, it is ensured that unrealistic solutions in the community model are excluded where a species altruistically synthesizes large amounts of byproducts (instead of biomass) to fulfill the requirements of other species. We employed the RedCom approach for modeling communities of up to nine organisms involved in typical degradation steps of anaerobic digestion in biogas plants. Compared to full (bilinear and linearized) community models, we found that the reduced community models obtained with RedCom are not only much smaller but allow, also in the largest model with nine species, extensive calculations required to fully characterize the solution space and to reveal key properties of communities with maximum methane yield and production rates. Furthermore, the predictive power of the reduced community models is significantly larger because they predict much smaller ranges of feasible community compositions and exchange fluxes still being consistent with measurements obtained from enrichment cultures. For an enrichment culture for growth on ethanol, we also used metaproteomic data to further constrain the solution space of the community models. Both model and proteomic data indicated a dominance of acetoclastic methanogens (Methanosarcinales) and Desulfovibrionales being the least abundant group in this microbial community.

## Author summary

Microbial communities are involved in many fundamental processes in nature, health and biotechnology. The elucidation of interdependencies between the involved players of microbial communities and how the interactions shape the composition, behavior and characteristic features of the consortium has become an important branch of microbiology research. Many communities are based on the exchange of metabolites between the species and constraint-based metabolic modeling has become an important approach for a formal description and quantitative analysis of these metabolic dependencies. However, the complexity of the models rises quickly with a growing number of organisms and the space of predicted feasible behaviors often includes unrealistic solutions. Here we present RedCom, a new approach to build reduced stoichiometric models of balanced microbial communities based on net conversions of the single-species models. We demonstrate the applicability of our RedCom approach by modeling communities of up to nine organisms involved in degradation steps of anaerobic digestion in biogas plants. As one of the first studies in this field, we compare simulation results from the community models with experimental data of laboratory-scale biogas reactors for growth on ethanol and glucose-cellulose media. The results also demonstrate a higher predictive power of the RedCom vs. the full models.

## Introduction

Microbial communities are of major importance for human health [1,2], geochemical cycles [3,4] and biotechnological processes [5–7]. Despite of their importance, most microbial communities are still poorly understood due to their complex nature. Mathematical modeling can help to uncover the interactions and dependencies of the members of these communities. Different modeling formalisms have been used to simulate microbial communities including stoichiometric models, which can be analyzed by constraint-based methods [8–18]. An increasing number of stoichiometric community models considers *balanced growth* as a key assumption stating that all organisms must grow with the same growth rate in a stable community [11,15,16]. One central goal of these models is the characterization and prediction of possible community compositions and the analysis of the different modes of cross-feeding between the involved organisms.

Stoichiometric models of microbial communities with balanced growth usually result in bilinear models, where, in some equations, independent variables are multiplied with each other. Thus, apart from their increased size, these models have a more complex nature than the linear metabolic models of single species. To make bilinear models amenable to established constraint-based modeling approaches, they can be linearized by fixing either the community growth rate [16] or the community composition [11,15]. In this study, we first provide a unified framework for setting-up, analyzing, and linearizing community models. Even in linearized community models, the application of certain constraint-based techniques becomes quickly infeasible with an increasing number of organisms. Furthermore, one shortcoming of existing methods for modeling of communities is that the solution space often contains unrealistic solutions (where, for example, a species behaves unrealistically altruistic to produce substrates needed by other community members). We therefore introduce a new approach, RedCom, to build reduced community models. The main principle of RedCom is similar to what has been suggested by Taffs et al. [10], namely to compute, in a first step, relevant net conversions of the single-species models which serve as reactions for the reduced model. This reduced model can then be used to identify suitable combinations of single-species net conversions to obtain community-level conversions. However, while Taffs et al. [10] used elementary modes to describe the single-species net conversions, RedCom is based on the more general concept of elementary flux vectors [19,20]. This will be required to ensure balanced growth in the community model and to appropriately account for flux bounds and other (e.g. proteome allocation) constraints. Reduced community models obtained with RedCom do not only focus on most relevant solutions but allow for a comprehensive characterization of solution spaces also for communities with more than only two or three species. In the following, we apply the proposed techniques for different community models with increasing complexity from three up to nine species. The investigated communities are capable of degrading different substrates to biogas, a renewable energy source. Community models of the biogas process give insights on interdependencies and feasible community compositions and may contribute to increase productivity and stability of this process. As one of the first studies, we also compare simulation results from the community models with experimental data of laboratory-scale biogas reactors for growth on ethanol and glucose-cellulose media.

## Methods

### Constraint-based modeling

Constraint-based (stoichiometric) modeling of metabolic networks [21] relies on the assumption of a steady-state for internal metabolite concentrations leading to the mass balance equation:

Nr=0 (1)

The structure of the network is captured by the stoichiometric matrix **N** storing the stoichiometric coefficients of the metabolites (rows) in the metabolic reactions (columns). As consequence of eq. (1), steady-state flux vectors **r** fulfill the condition that no net accumulation or depletion of internal metabolites occurs. Additionally to the steady-state condition, reversibility constraints (2), flux bounds (3) and other types of inhomogeneous linear constraints (4) can be included:

$$r_{j}\geq 0\;for\;j\in Irrev$$ (2)

$$\alpha_{j}\leq r_{j}\leq \beta_{j}$$ (3)

Ar≤b. (4)

The set *Irrev* contains the indices of irreversible reactions. If only the steady-state (1) and the irreversibility constraints (2) are taken into account, the solution space forms a polyhedral (flux) cone; with any constraint of type (3) or (4) its shape becomes a (flux) polyhedron.

### Assembling a compartmented community model from single-species models

**Fig 1:**
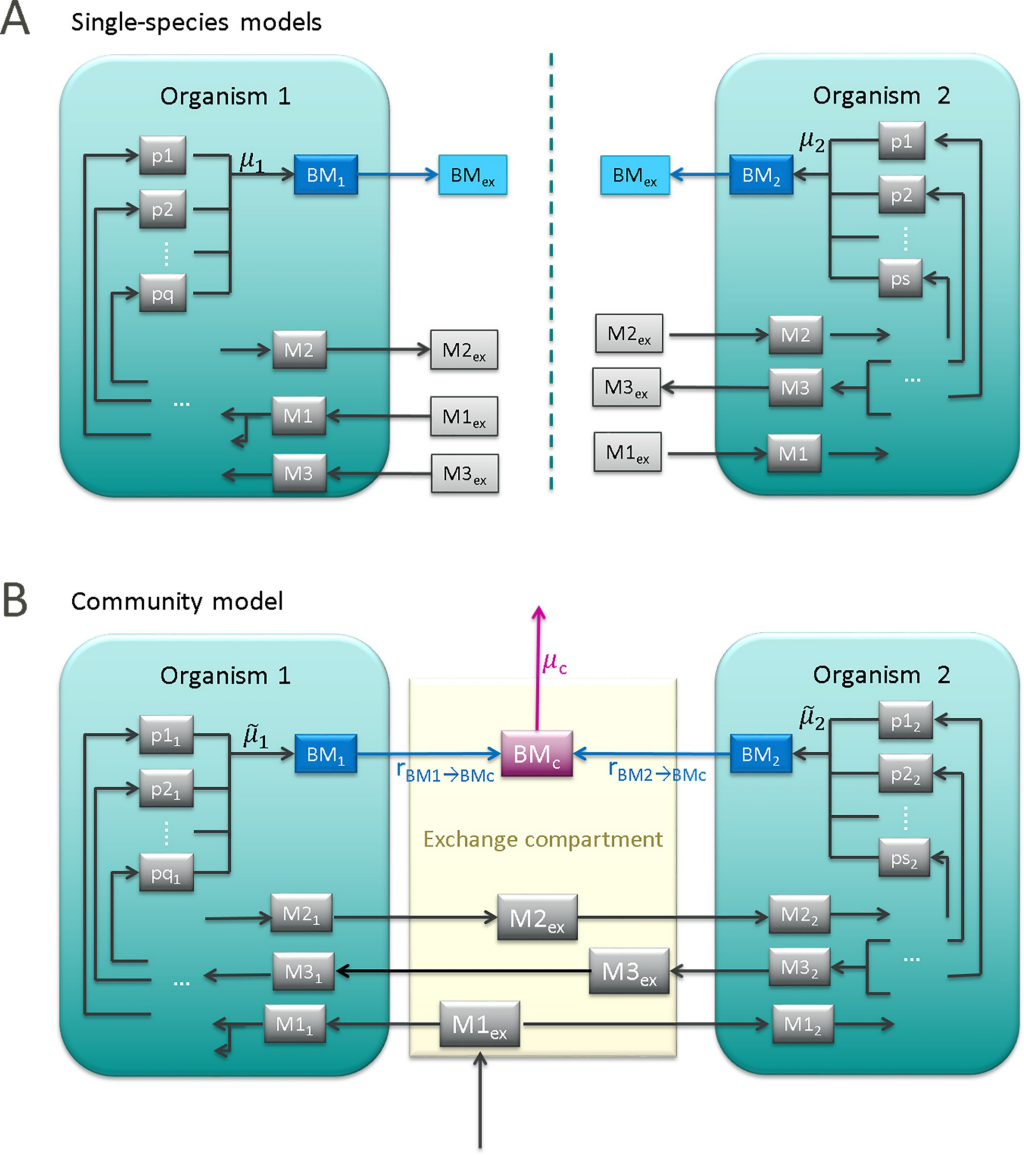
**Schematic overview of the structure of single-species models (A) and the resulting community model (B).** Metabolites are indicated with boxes, reactions are represented by arrows. External metabolites in single-species models become internal metabolites in the community model.

In order to create a community model combining all (*n*) single-species models, herein referred to as full model, a compartmented approach is usually employed [9,11,12,15,22,23]. Each organism represents one compartment and an additional exchange compartment allows for exchange of metabolites (substrates/products) between organisms and with the medium (Fig 1). With the new exchange compartment, the former external (unbalanced) metabolites become now internal ones and must be balanced in eq. (1). Exchange metabolites used by several species are combined such that they exist only once in the community model.

As described in [15] the units of the (specific) single-species reaction rates must be adapted to refer to the total community (instead of single-species) biomass. Accordingly, the units of all reaction rates change from mmol/gDW_*i*_/h to mmol/gDW_*c*_/h. Exceptions are the *n* biomass synthesis (growth) reactions producing the species biomasses BM_*i*_ from a (species-specific) set of precursors:

$$\gamma_{i,1}p_{i,1}+\gamma_{i,2}p_{i,2}+\cdots +\gamma_{i,q}p_{i,q}\to 1\;BM_{i}\;[gDW_{i}]\;(i=1\ldots n)$$ (5)

In the single-species models, the specific (growth) rates *μ*_*i*_ (*i* = 1…*n*) of these *n* reactions referred to unit 1/h, which is now changed to gDW_*i*_/gDW_*c*_/h. We indicate the changed units of these reaction rates in the community model by the symbol ${\tilde{\mu }}_{i}(i=1\ldots n).$

Furthermore, *n* new pseudo-reactions are introduced in the community model to describe the integration of the *n* species biomasses into the community biomass BM_*c*_ (Fig 1):

$$1\;BM_{i}\left[gDW_{i}\right]\to 1\;BM_{c}\left[gDW_{c}\right]\;(\mathrm{rate}:\;r_{BM_{i}\to BM_{c}}\;[gDW_{i}/gDW_{c}/h])\;(i=1\ldots n)$$ (6)

Finally, a new *community growth reaction* is introduced “exporting” the synthesized community biomass to the medium (Fig 1); the rate of this reaction is the community growth rate μ_*c*_ [1/h]:

$$1\;BM_{c}\left[gDW_{c}\right]\to \;\left(\mathrm{rate}:\;\mu_{c}\left[1/h\right]\right)$$ (7)

Note that, in steady state, ${\tilde{\mu }}_{i}=r_{{BM}_{i}{\to BM}_{c}}$ and $\sum_{i=1}^{n}{\tilde{\mu }}_{i}=\sum_{i=1}^{n}r_{{BM}_{i}{\to BM}_{c}}=\mu_{c}$. The obtained structure of the whole community network is captured in the community stoichiometric matrix **N**^*c*^ and the reaction rates in the community flux vector **r**^*c*^ (with units as described above). As for the single-species models, we demand steady-state for the metabolites (including all metabolites in the exchange compartment):

$$N^{c}r^{c}=0.$$ (8)

In a stable continuous culture, the growth rate of microorganisms is typically equal to the dilution rate. We assume that the same is true for a microbial community cultivated in a continuous process. In that case, the growth rates *μ*_*i*_ of all organisms (each normalized to the respective specific biomass) must be identical and equal the community growth rate *μ*_*c*_:

$$\mu_{1}=\mu_{2}=\cdots =\mu_{n}=\mu_{c}.$$ (9)

This concept of *balanced growth* of microbial communities has previously been proposed by Khandelwal et al. (2012) and is also an underlying principle of the OptDeg [15] and the SteadyCom [16] approach. It has been argued that, even if there is no steady state in a continuous cultivation, the specific growth rates of the organisms need to be the same on average because otherwise the fastest organism would outgrow the others. With constant growth rates, also the fractional biomass abundances

$$F_{i}=\frac{{\mathrm{BM}}_{i}}{{\mathrm{BM}}_{c}}$$ (10)

of each species *i* in the community biomass BM_*c*_ must be constant. The fractions *F*_*i*_ define the community composition **F** = (*F*_1_,…,*F*_*n*_) and sum up to unity:

$$\sum_{i=1}^{n}F_{i}=1.$$ (11)

With balanced growth, the fraction *F*_*i*_ of species *i* is given by the ratio of the specific biomass production rate of species *i* (normalized to the community biomass) and the community growth rate:

$$F_{i}=\frac{{\mathrm{BM}}_{i}}{{\mathrm{BM}}_{c}}=\frac{r_{BM_{i}\to BM_{c}}}{\mu_{c}}=\frac{{\tilde{\mu }}_{i}}{\mu_{c}}$$ (12))

Note that the fractional contributions to the synthesis of the community biomass (${\tilde{\mu }}_{i}=r_{{BM}_{i}{\to BM}_{c}}$; normalized to BM_*c*_) are not identical over the species, hence, the ${\tilde{\mu }}_{i}$ need not fulfill (9). However, for the specific growth rates *μ*_*i*_ (referring to BM_*i*_) it holds that $\mu_{i}={\tilde{\mu }}_{i}/F_{i}=\mu_{c}$ and thus (9) is indeed satisfied. For each species, we can rewrite (12) to the following constraint:

$$r_{BM_{i}\to BM_{c}}=F_{i}\mu_{c}.$$ (13)

(Alternatively we could also use ${\tilde{\mu }}_{i}$ instead of $r_{{BM}_{i}{\to BM}_{c}}$ in this equation). In the optimization problems considered below, constraints of type (13) need to be included only for *n*−1 species, because (6), (7), and (11) already imply (13) for the *n*-th species: $r_{{BM}_{n}{\to BM}_{c}}=\mu_{c}-\sum_{i=1\ldots n-1}r_{{BM}_{i}{\to BM}_{c}}=\mu_{c}-\sum_{i=1\ldots n-1}F_{i}\mu_{c}=\mu_{c}-(1-F_{n})\mu_{c}={F_{n}\mu }_{c}$.

Due to the re-normalization of the reaction rates from specific to community biomass, as the last step in assembling the community model we also need to adjust the normalization of the original flux bounds (3) and other inhomogeneous conditions (4) by multiplying them with the fractional abundances:

$$F_{i}\alpha_{ij}\leq r_{ij}^{c}\leq F_{i}\beta_{ij}$$ (14)

where *α*_*ij*_ and *β*_*ij*_ are the lower and upper bounds for reaction *j* in organism *i* and $r_{ij}^{c}$ is the reaction rate of reaction *j* in organism *i* in the community model. Likewise, constraints (4) are adjusted for each organism to

$$A_{i}r_{i}^{c}\leq F_{i}b_{i}$$ (15)

(**A_*i*_**, **b**_*i*_ correspond to the respective variables in (4) for species *i*). The irreversibility constraints for the reaction rates are kept from the single-species models:

$$r_{ij}^{c}\geq 0\;\mathrm{for}\;j\in Irrev_{i}.$$ (16)

To exclude solutions with non-zero fluxes $r_{ij}^{c}\neq 0$ in organisms that are not present in the community (*F*_*i*_ = 0), we assume that every flux in species *i* is bounded (i.e., *α*_*ij*_ and *β*_*ij*_ are bounded). With (14), a non-zero flux $r_{ij}^{c}$ then implies *F*_*i*_>0. In principle, with the chosen constraints, one can also consider the case where the community is not growing (*μ*_*c*_ = 0), i.e., where dependencies arise exclusively from the maintenance metabolism of the participating species. However, if the community is growing (*μ*_*c*_>0), a non-zero flux $r_{ij}^{c}\neq 0$ in species *i* implies again *F*_*i*_>0 and, due to (13), then also $r_{{BM}_{i}{\to BM}_{c}}={\tilde{\mu }}_{i}>0$.

In analogy to classical flux balance analysis (FBA) in single organisms, we may formulate a (linear) objective function maximizing certain (combinations of) reaction rates in the community model:

$$\begin{array}{c} Maximize\;c^{T}r^{c}\\ s.t.\;\left(8\right),\;\left(11\right),\;\left(13\right)-\left(16\right) \end{array}$$ (17)

Due to the multiplication of (independent) variables in constraints (13), the community model and the associated optimization problem become bilinear. While non-linear solvers can be employed to solve the optimization problem (e.g., to search for maximum community growth rates or to scan feasible ranges of fluxes or community compositions; see below), a linearization can be applied to enable application of standard linear programming solvers and methods routinely used in (linear) constraint-based modeling.

### Linearization of the community model

**Fig 2:**
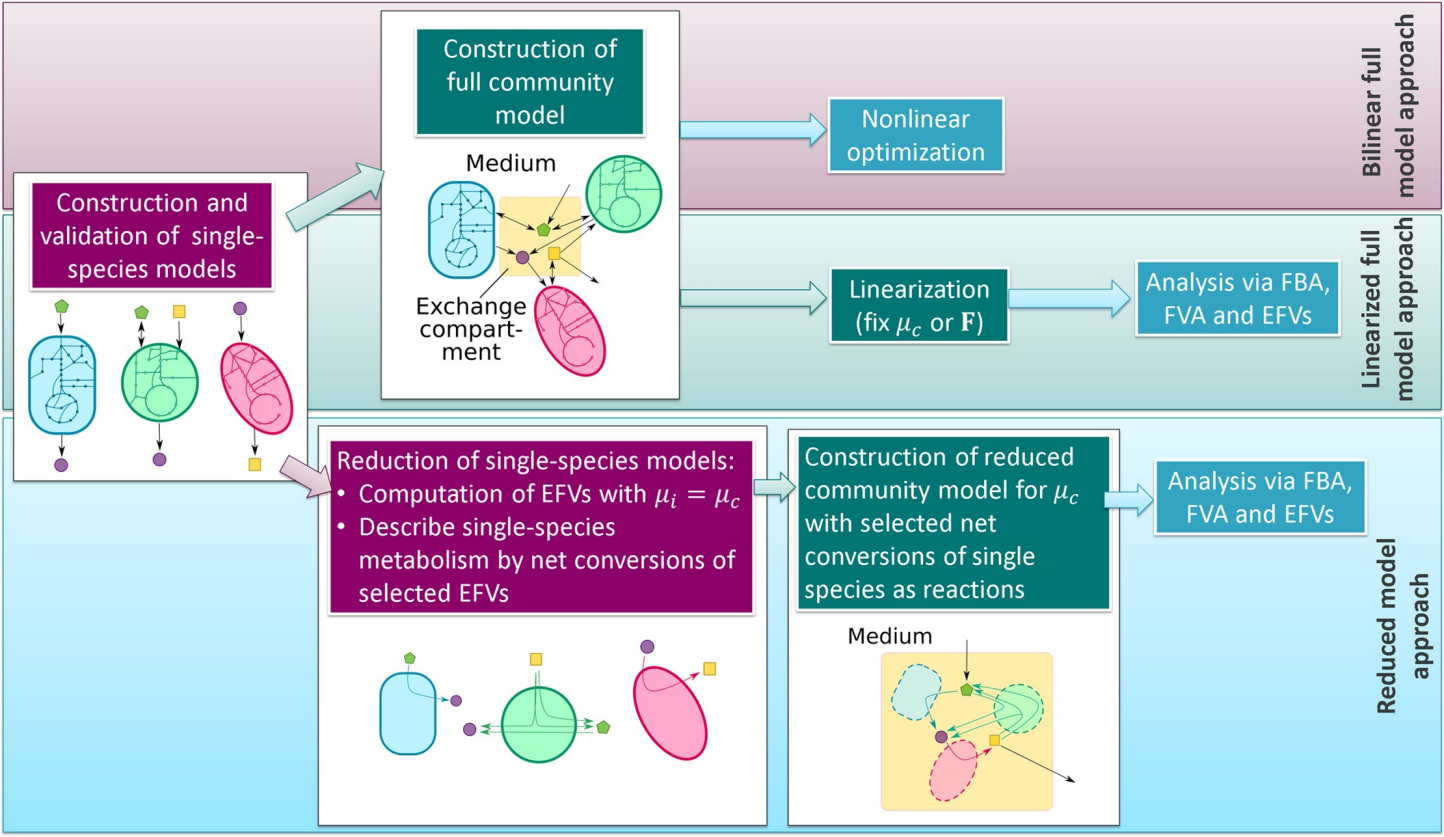
Workflow of constructing three different types of community models considered in this study.

Two approaches have been used to linearize bilinear community models and to simplify its analysis (Fig 2). In the first approach (utilized in SteadyCom [16]), the community growth rate *μ*_*c*_ is fixed to a constant (known) value. The constraints (13) become then linear and the optimization problem (17) thus treatable with standard linear programming (LP) solvers. Linearization by fixing the community growth rate is useful, for example, to analyze which community compositions are feasible for a given community growth rate. Repeating these analyses (in discrete steps) for the feasible range of community growth rates yields a more complete picture of the whole solution space.

An alternative linearization method was used in community FBA [11] and in the OptDeg approach [15]. Here, instead of the community growth rate, the community composition, i.e. all the fractional abundances *F*_*i*_, are fixed. Eq (13) becomes then again linear allowing the utilization of LP solvers. With given fractional abundances, constraint (11) can be removed from the optimization problem (17).

This second linearization approach is useful to scan, for example, the feasible community flux space for a given community composition. However, with a growing number of organisms, this scanning becomes very expensive in terms of the number of linear programs to be solved [16]. In this study, we therefore linearize community models by fixing *μ*_*c*_ as proposed in the SteadyCom approach. We used an iterative approach to find the maximum community growth rate *μ*_*c*,*max*_ in these linearized models. First, we set *μ*_*c*_ to a value of 0.005 h^-1^. If a feasible flux distribution exists (here, any (including a zero) objective function can be used in Eq (17)), we double *μ*_*c*_ and check again for a feasible flux distribution. We repeat these steps until no feasible flux distribution is found. We then take the average of this *μ*_*c*_ and the last feasible *μ*_*c*_ (or zero if the first *μ*_*c*_ did not yield a flux distribution). These steps are repeated (check for feasibility, use average of latest feasible and infeasible *μ*_*c*_ as new constraint and check again for feasibility) until the difference between the last feasible and infeasible *μ*_*c*_ is smaller than 0.00001 h^-1^.

Generally, for both linearization variants, apart from the FBA-like optimization in (17), other constraint-based methods like flux variability analysis (FVA) or metabolic pathway analysis based on elementary flux modes or elementary flux vectors can be carried out (see below).

### Species-level optimality in the community models

The described approaches for modeling communities under balanced growth can be used to define and analyze community solution spaces. However, these solution spaces often include unrealistic solutions on the species-level (e.g., a species synthesizes, without any benefit for its own growth, products required by another species in the community [15]). Consequently, the predicted ranges for community compositions or growth rates may become very large as they include many non-relevant phenotypes. FBA could be used to find community compositions fulfilling certain optimality criteria, but the question of suitable objective function in communities arises. In single-species models, a typical objective function is maximization of the growth rate. In community models we can also maximize the community growth rate [11]. But, again, even these optimal solutions may represent unrealistic community compositions in which some organisms waste substrate to ensure survival of the others [15]. We therefore proposed previously an optimization approach to minimize a weighted sum of substrate uptake rates to find community compositions in which all organisms grow with their maximum biomass yields [15]. This approach enabled us to narrow down the solution space to community compositions in which all organisms grow optimally with their maximum biomass yields at a given community growth rate. When introducing our model reduction approach below, we will use a similar method to exclude unrealistic community flux distributions.

### Elementary flux modes and elementary flux vectors

Elementary flux modes (EFMs) are non-decomposable flux vectors fulfilling Eqs (1) and (2) [24]. EFMs represent balanced pathways or cycles and have become an important tool for exploring metabolic networks [20,25–28]. However, one shortcoming of EFMs is that inhomogeneous constraints (Eqs (3) and (4) in the single-species models and (14) and (15) in the community model), such as non-growth associated ATP maintenance demand and substrate-uptake limits, cannot be considered. We therefore make use of the concept of elementary flux vectors (EFVs), a generalization of EFMs which can account for inhomogeneous constraints [19,20]. From the theory of EFVs, it is known that the flux polyhedron **P** resulting from a set of linear constraints is generated by convex combinations of bounded EFVs **p**^*k*^ and conic linear combinations of unbounded EFVs **x**^*i*^ and **y**^*j*^:

$$P=\{r\in {ℜ}^{n}|r=\sum_{k\in K}\gamma_{k}p^{k}+\sum_{i\in I}\alpha_{i}x^{i}+\sum_{j\in J}\beta_{j}y^{j},\;\gamma_{k}\geq 0,\sum_{k\in K}\gamma_{k}=1,\;\alpha_{i}\geq 0\}$$ (18)

Due to combinatorial explosion, EFVs can usually only be calculated in medium-scale metabolic networks and, thus, only in smaller community models combining the central metabolism of two or three species.

### RedCom: Building reduced community models with balanced growth from net conversions

We present RedCom, a new method to generate community models of reduced size and with reduced solution spaces excluding unrealistic community behaviors. The main idea of the reduction approach, which has some similarities with but is not identical to an approach presented by Taffs et al. [10], is to describe the metabolism of each organism by certain net conversions taken from the EFVs of the single-species models (Fig 2). Since we are mainly interested in community compositions and metabolic interactions (exchange reactions) between the community members, it is often sufficient to focus only on net conversions of the respective species instead of taking its whole metabolic reaction network explicitly into account. Furthermore, from the list of all net conversions of a species we select only those that obey certain optimality criteria avoiding unrealistic phenotypes in the community model. The selected net conversions are used as reactions in the reduced community model to be built. The construction of reduced community models with the RedCom approach is described in the following, a detailed example is given in S1 Text in the Supplements.

#### Computation of EFVs

For obtaining the reduced community model, the (community) growth rate *μ*_*c*_ is fixed to a particular (e.g., measured) value *μ*_*c*,*fix*_ and the growth rate in the single-species models is then also set to this value: *μ*_*i*_ = *μ*_*c*,*fix*_. Afterwards, all bounded and unbounded EFVs are calculated for each single-species network. Apart from the fixed community growth rate, other frequently used inhomogeneous constraints are (known) upper bounds for substrate uptake and product formation rates as well as a lower bound for the rate of the ATP consuming pseudo reaction reflecting the maintenance coefficient (*r*_*ATPmaint*_; Table 1). We can assume that all thermodynamically feasible solutions involve substrate uptake (internal cycles are discarded) and that substrate uptake is bounded by some upper limit. Therefore, we only need to consider bounded EFVs (**p**^*k*^ in Eq (18)) in our approach.

**Table 1:** Overview of the single-species models with model dimensions and constraints used for the community model. The last three columns indicate which models were used to build the three-, six- and nine-species community models.

Organism	# internal metabolites × #reactions	Constraints [mmol/gDW/h]	Number of EFVs (for *μ*_*c*_ = 0.008 h^-1^)	Three-species model	Six-species model	Nine-species model
*Escherichia coli* (EC)	99 x 117 Substrates: O_2_, Glyc, Glucn, Glc, CO_2_ Products: CO_2_, Succ, Lac, Eth, Ac, Form	r_ATPmaint_≥3.15 r_Glcup_≤18.5 r_Glycup_ = 0 r_Glucup_ = 0 r_O2up_ = 0	60653			x
*Acetobacterium woodii* (AW)	107 x 116 Substrates: Glc, H_2_, Eth, CO_2_, Lac, Fruc, Form, MeOH Products: Ac, CO_2_	r_ATPmaint_≥0.29 r_Glcup_≤2.75 r_Frucup_ = 0 r_Lacup_≤3 r_Ethup_≤9 r_MeOHup_ = 0 r_H2up_≤19.5 r_Formup_≤19.5 μ≤0.162	4582		x	x
*Propionibacterium freudenreichii* (PF)	105 x 111 Substrates: Lac, Eth, Glc, CO_2_ Products: Prop, Ac, Succ, CO_2_	r_ATPmaint_≥0.76 r_Glcup_≤4 r_lacup_≤11 r_EthOHup_≤11 μ≤0.16	23878		x	x
*Clostridium acetobutylicum* (CA)	111 x 123 Substrates: Glc, Glyc, Ac, Buty Products: Lac, Eth, Buty, Butol, Ac, Acon, Form, CO_2_, H_2_	r_ATPmaint_≥1 r_Glcup_≤12.75 r_Glyc_up_ = 0 r_Acon_ex = 0 r_Buolex_ = 0	1596			x
*Syntrophobacter fumaroxidans* (SF)	104 x 114 Substrates: Prop, Fum SO_4_ Products: Ac, Form, Succ, CO_2_, H_2_, H_2_S	r_ATPmaint_≥0.14 r_Propup_≤1.6 r_Fumup_≤0.5 r_SO4up_ = 0	39932		x	x
*Syntrophomonas wolfei* (SW)	110 x 114 Substrates: Buty, Crot Products: Buty, Ac, H_2_, Form, CO_2_	r_ATPmaint_≥0.14 r_Butup_≤4.25 r_Crotup_≤2.85	443			x
*Desulfovibrio vulgaris* (DV)	99 x 115 Substrates: Pyr, Lac, Eth, Ac, CO_2_, H_2_, SO_4_ Products: Ac, Form, CO_2_, H_2_, H_2_S	r_ATPmaint_≥4.3 r_Acex_≤50 r_SO4up_ = 0	840	x	x	x
*Methanosarcina barkeri* (MB)	96 x 103 Substrates: Ac, MeOH, H_2_, CO_2_ Products: CH_4_, CO_2_	r_ATPmaint_≥2.5 r_CH4ex_≤15	35	x	x	x
*Methanospirillum hungatei* (MH)	95 x 102 Substrates: H_2_, CO_2_, Form Products: CH_4_, CO_2_	r_ATPmaint_≥0.9 r_CH4ex_≤15	23	x	x	x

#### Selecting EFVs with minimal conversions

In a next step, for each organism, we select the EFVs, which we consider as relevant (realistic) “phenotypes”. Criteria for EFV selection can be adjusted as needed. Herein, we select EFVs with minimal (parsimonious) exchange fluxes and discard solutions, which lead to inefficient substrate conversion (e.g. with low biomass or/and ATP yields) and may result in unrealistic altruistic behavior of an organism in a given community. For this purpose, we project each EFV **e** to its (*q*) exchange reactions including the growth rate: $e=\left(\begin{array}{c} e_{1}\\ \vdots \\ e_{q-1}\\ e_{\mu } \end{array}\right)$. Next, to avoid redundancies, for EFVs with identical projection on the exchange reactions, we keep only one candidate. Assume now we have, for a given species, *s* non-redundant (projected) EFVs. We discard an EFV **e**^*k*^ if there exists a convex combination **w** of other projected EFVs in which all rates of the exchange reactions are smaller than or equal to the ones in **e**^*k*^. This condition is fulfilled (and **e**^*k*^ removed) if a solution **w** exists for the following system:

$$\begin{array}{c} E^{*}w\leq e^{k}\\ \left(1\cdots 1\right)w=1\\ w\geq 0 \end{array}$$ (19)

with E* = [**e**^1^…**e**^*k*−1^ **e**^k+1^…**e**^*s*^]. Note that the combination of the EFVs must be convex (second row in (19)) to ensure that the predefined growth rate and other inhomogeneous constraints are met. We thus only keep EFVs whose total turnover of at least one of the external metabolites is smaller compared to a convex combination of the other EFVs (recall that redundant projected EFV had been discarded before). In particular, this criterion ensures that, for a given combination of substrate(s) and product(s), only biomass-yield optimal EFVs will be kept. If there are, for a given substrate, different potential products, then at least one EFV will be kept for each product, even if it is biomass-yield suboptimal compared to another product. The reason is as follows: in some cases, accumulation of certain products in the medium can inhibit growth and result in a shift to another pathway. With the approach described above, we keep these options in the model to retain a certain metabolic flexibility. We save the retained EFVs in Matrix **E** (each row in **E** represents one selected projected EFV).

#### Net conversions from EFVs and reduced single-species model

Next, we calculate the stoichiometric matrix **N**^red^ of the reduced (single-species) network:

$$N^{red}=N^{EX}∙E.$$

(**N**^EX^ is the sub-matrix of **N** containing the columns and rows corresponding to the exchange reactions and external metabolites.) Hence, the net conversions (in terms of external metabolites) of the selected EFVs become now reactions (columns in **N**^red^) in the reduced model. The reversibility of a reaction in **N**^red^ depends on the reversibility of the respective EFV from which it was derived. Since the EFVs represent net conversions of substrates to products and biomass, the reactions are normally all irreversible.

#### Constructing the community model

We repeat these steps for all *n* organisms of the community. Afterwards we proceed as described earlier to construct the (reduced) community model from the (reduced) single-species models. By their nature, each reduced species network consists only of exchange metabolites and (overall) reactions converting them. Therefore, effectively only two compartments need to be considered in the community model: the exchange compartment and the medium (see Fig 2). However, each reaction is associated with exactly one of the species. Exchange metabolites occurring in several species are again combined such that they only exist once in the community model. All exchange metabolites, that are not allowed to accumulate in the medium, must fulfill the mass balance Eq (8) and the community growth rate is fixed to the value used in the reduced single-species models (*μ*_*c*_ = *μ*_*c*,*fix*_) by which the reduced community model becomes linear. Importantly, no other inhomogeneous constraints need to be considered; all flux bounds (and other constraints) from the original single-species models are automatically fulfilled in the reduced community model as long as *μ*_*c*_>0 (see S2 Text in the Supplements). S1 Text in the Supplements illustrates the construction of a reduced community model for an example community and also explains how the special case of *μ*_*c*_ = 0 can be treated by a minor modification of the reduced community model.

The linear reduced community model can be explored with standard analysis methods of constraint-based modeling. In particular, the feasible community compositions **F** can be determined and studied. Furthermore, “community EFVs” can be computed and analyzed (which should not be confused with the EFVs computed in the original single-species models to construct the reduced single-species models).

### Calculations

All models presented in the Results section were implemented and analyzed with *CellNetAnalyzer* version 2018.1, a MATLAB package for structural and functional analysis of metabolic and signaling networks [29,30]. CPLEX was used as a solver for linear optimizations and *efmtool* for computation of EFVs. For solving bilinear problems, we used the *fmincon* solver for nonlinear optimization in MATLAB. The solver needs an initial flux distribution that we retrieved from the linearized model.

### Data from laboratory-scale biogas reactor on glucose-cellulose medium

Experimental data from a laboratory-scale biogas reactor on a defined glucose-cellulose medium were published earlier [31] and used for a comparison with predictions from the nine-species biogas producing community (see Results). The data were taken from steady-state conditions [31]. We calculated the average methane and CO_2_ production rates over a course of 100 days. To achieve steady-state conditions, the reactors were operated under similar conditions for 190 days prior to this time period. Additionally to the data already published, we estimated biomass dry weights by measuring protein concentrations with the Lowry Assay [32] and dividing them by the factor 0.64 (assumed fraction of protein of the total biomass in the model). We used these data to calculate specific production and consumption rates for comparison with simulation results.

### Continuous enrichment cultures on ethanol

A detailed description of the procedures applied for inoculation, feeding, and sample analyses along with cultivation setup and parameters is given in the S6 Text. Briefly, two 1.5 L bioreactor systems were inoculated with sludge from the aforementioned enrichment and fed with the same medium containing 14.6% (v/v) ethanol as main carbon source instead of glucose and cellulose. After an adaption period, continuous cultivation mode was initiated using constant feeding rates and volume control. In the following, different dilution rates were sampled at steady-state conditions, starting from 5.3∙10^−4^ h^-1^ further increasing until the biogas production collapsed. Sampling and subsequent analyses comprised pH, biomass protein content, biogas composition and biogas volume produced. In addition, samples were analyzed for residual ethanol and accumulated organic acids. Finally, taxonomic analysis was carried out using an established MS-based metaproteomic workflow (see S8 Text).

## Results

In the following, we first describe the construction of metabolic models of nine organisms capturing major degradation steps in the biogas process. Subsequently we combine these single-species models to community models of increasing complexity containing three, six, and nine strains. For each considered community, we construct, analyze and compare three different types of models (bilinear model, linearized full model, reduced model obtained with RedCom) as described in the Methods section. For the six- and nine-species communities, we compare model predictions with experimental data.

### Model organisms

Using KEGG [33] and MetaCyc [34] as well as various literature references we manually constructed single-species models of the central metabolism of nine different organisms all being relevant for the biogas process: four primary fermenting bacteria (*Acetobacterium woodii*, *Escherichica coli*, *Clostridium acetobutylicum*, *Propionibacterium freudenreichii*), three secondary fermenting bacteria (*Syntrophomonas wolfei*, *Syntrophobacter fumaroxidans*, *Desulfovibrio vulgaris*) and two methanogenic archaea (*Methanospirillum hungatei* and *Methanosarcina barkeri)*. As suggested by Taffs et al. [10], we consider each of these organisms as one functional guild in the biogas process with certain metabolic properties. More specifically, under anaerobic conditions, *E*. *coli* produces ethanol as well as different organic acids like formate, lactate, acetate and succinate from glucose, glycerol and gluconate. *A*. *woodii* is an homoacetogenic organism that can either ferment sugars like glucose and fructose but also lactate, formate or hydrogen and CO_2_ to produce acetate via the Wood-Ljungdahl pathway [35,36]. *P*. *freudenreichii* can ferment glucose, glycerol and lactate to succinate and propionate. The organism uses the methyl-malonyl-CoA pathway to produce propionate. Some organisms using the methyl-malonyl-CoA pathway like *Pelobacter propionicus* are also capable of using ethanol as a substrate [37]. Since we aimed to represent the functional guild of propionate producing bacteria using the methyl-malonyl-CoA pathway, we also added ethanol oxidation to propionate to the model. *C*. *acetobutylicum* ferments glucose and glycerol to different organic acids and solvents like acetate, butyrate, ethanol, butanol and aceton. The organism is known to grow in two different phases [38]. In the first phase, the organism produces organic acids like acetate and butyrate. These pathways have high ATP yields but the acids produced lower the pH in the medium. In the second phase, acids are taken up and solvents like butanol and aceton are the main product. *C*. *acetobutylicum* represents primary fermenting bacteria in our community model and we assumed that mainly production of formate, acetate, butyrate and ethanol is relevant in anaerobic digestion. We therefore disabled production of the other solvents in the community model. *D*. *vulgaris* is a sulfate-reducing bacterium that can grow on organic substrates like pyruvate, lactate and ethanol using sulfate or thiosulfate as an electron acceptor. In the absence of electron acceptors, the organism can also grow in syntrophic associations with hydrogen utilizing organisms. The products formed by *D*. *vulgaris* are either acetate and hydrogen plus CO_2_ or formate (in syntrophic cultures) or acetate plus hydrogen sulfide (when sulfate is present). Additionally, the organism can utilize hydrogen with acetate as a carbon source and sulfate as an electron acceptor. *S*. *fumaroxidans* can grow on propionate in syntrophy or with sulfate as an electron acceptor [39]. In pure culture the organism can grow on fumarate, fumarate plus propionate or succinate, formate or hydrogen plus sulfate [39]. *S*. *wolfei* is a secondary fermenting bacterium that can degrade saturated fatty acids from butyrate through octanoate either to acetate and hydrogen (even number of C-atoms) or to acetate, propionate and hydrogen (odd number off C-atoms) in syntrophic cultures [40]. Growth of *S*. *wolfei* is also possible on crotonate in monoculture [41].

The methanogenic organism *M*. *hungatei* (cytochrome-free) produces methane from formate or from hydrogen plus CO_2_ while *M*. *barkeri* (possesses cytochromes) can use hydrogen plus CO_2_, acetate, methanol and methylamines for methanogenesis. In addition to different substrates utilized by the methanogens they also differ in ATP yields and substrate affinities. *M*. *barkeri* has higher ATP yields but lower substrate affinity for hydrogenotrophic methanogens. In our *M*. *barkeri* model we only implemented methanogenesis from acetate, methanol, and hydrogen with CO_2_.

A summary of the single-species models with model dimensions (number of metabolites and reactions) and constraints is given in Table 1. The models of *D*. *vulgaris*, *M*. *barkeri* and *M*. *hungatei* were published before [15]. We estimated flux bounds for substrate uptake and product formation from experimental data or existing models, partially also from closely related organisms (see S3 Text). Maintenance coefficients (*r*_*ATPmaint*_) were taken from literature data but the reported values varied by more than one order of magnitude between the different species (Table 1, S3 Text). Below we will therefore carry out a sensitivity analysis to investigate the influence of the maintenance coefficients on simulation results. For model validation, we also compared model predictions with measured biomass yields reported in the literature (see S4 Text). All models are listed (and also provided in SBML format) in S1 Table in the Supplements.

For the simulations performed in this work, we focused on ethanol (three and six-species community) and glucose (nine-species community) as the only available substrates and switched the uptake of other substrates (glycerol, gluconate, methanol, fructose, sulfate) off to reflect the composition of media used in the experiments.

### Simulations of a three-species community

We investigated a three-species community model (Table 2) consisting of *D*. *vulgaris*, *M*. *hungatei* and *M*. *barkeri*. This community can convert ethanol to methane, CO_2_, and acetate and thus covers the last two steps of anaerobic digestion. A similar community was experimentally investigated by Tatton et al. [42] and simulated with FBA in a previous study [15]. In analogy to the study of Tatton et al. [42], the uptake of external CO_2_ was allowed to also include solutions in which the acetoclastic methanogen is non-essential.

**Table 2:** Model dimensions of full and reduced community model as well as the number of computed EFVs for a chosen scenario. Additionally, the chosen substrate for the community is listed. EFVs could not be computed for communities with five or more species in the full linearized model. For species abbreviations see Table **1**.

Number of organisms	#internal metabolites × #reactions		Number of flux-bound constraints	Number of EFVs (for *μ*_*c*_ = 0.008 *h*^−1^)		Substrate (products)
	Full model	Reduced model		Full model	Reduced model	
2 (DV, MM)	205 x 233	13 x 24	6	254	2	Lactate (CH_4_,CO_2_,Ac)
3 (+ MB)	302 x 337	15 x 29	8	13,574	13	Ethanol, CO_2_ (CH_4_,CO_2_,Ac)
4 (+ AW)	411 x 454	18 x 44	17	380,800	14	Ethanol (CH_4_,CO_2_,Ac)
5 (+ PF)	523 x 568	25 x 80	22	-	28	Ethanol (CH_4_,CO_2_,Ac, Prop)
6 (+ SF)	628 x 684	27 x 115	26	-	172	Ethanol (CH_4_,CO_2_,Ac, Prop)
7 (+ CA)	742 x 813	31 x 129	29	-	51,021	Glucose (CH_4_,CO_2_,Ac, Prop, Buty)
8 (+ SW)	854 x 929	34 x 137	32	-	70,074	Glucose (CH_4_,CO_2_,Ac, Prop, Buty, Mal)
9 (+ EC)	953 x 1,048	35 x 158	37	-	147,694	Glucose (CH_4_,CO_2_,Ac, Prop, Buty, Mal)

#### Simulations with the full (bilinear) model

We constructed the community model by combining the models of the three participating species in a compartmented approach (see Methods). Initially, neither the community composition **F** nor the community growth rate *μ*_*c*_ were fixed resulting in a bilinear community model (see Methods). We used this bilinear model and a non-linear solver to compute the maximum community growth rate *μ*_*c*,*max*_ of the three-species community for growth on ethanol and obtained *μ*_*c*,*max*_ = 0.052 h^-1^. As another important characteristic of a community model, we next computed the possible ranges of the fractional abundances (*F*_*i*_), of the exchange reactions, and of the methane yield (by separately minimizing and maximizing each off these values). We considered two different scenarios (with and without allowed accumulation of acetate) and the results can be found in S5 Text. Generally, the bilinear model predicts broad ranges for exchange rates, community composition, and methane yield. This is not surprising given that these predictions are made for all possible growth rates (dilution rates). The bilinear models also predicts correctly that *D*. *vulgaris* is essential for the community while the methanogens are compositionally variable in the scenario with allowed acetate accumulation and *M*. *barkeri* essential in the second scenario where no acetate accumulation is allowed.

#### Linearized model with fixed community growth rate

**Fig 3:**
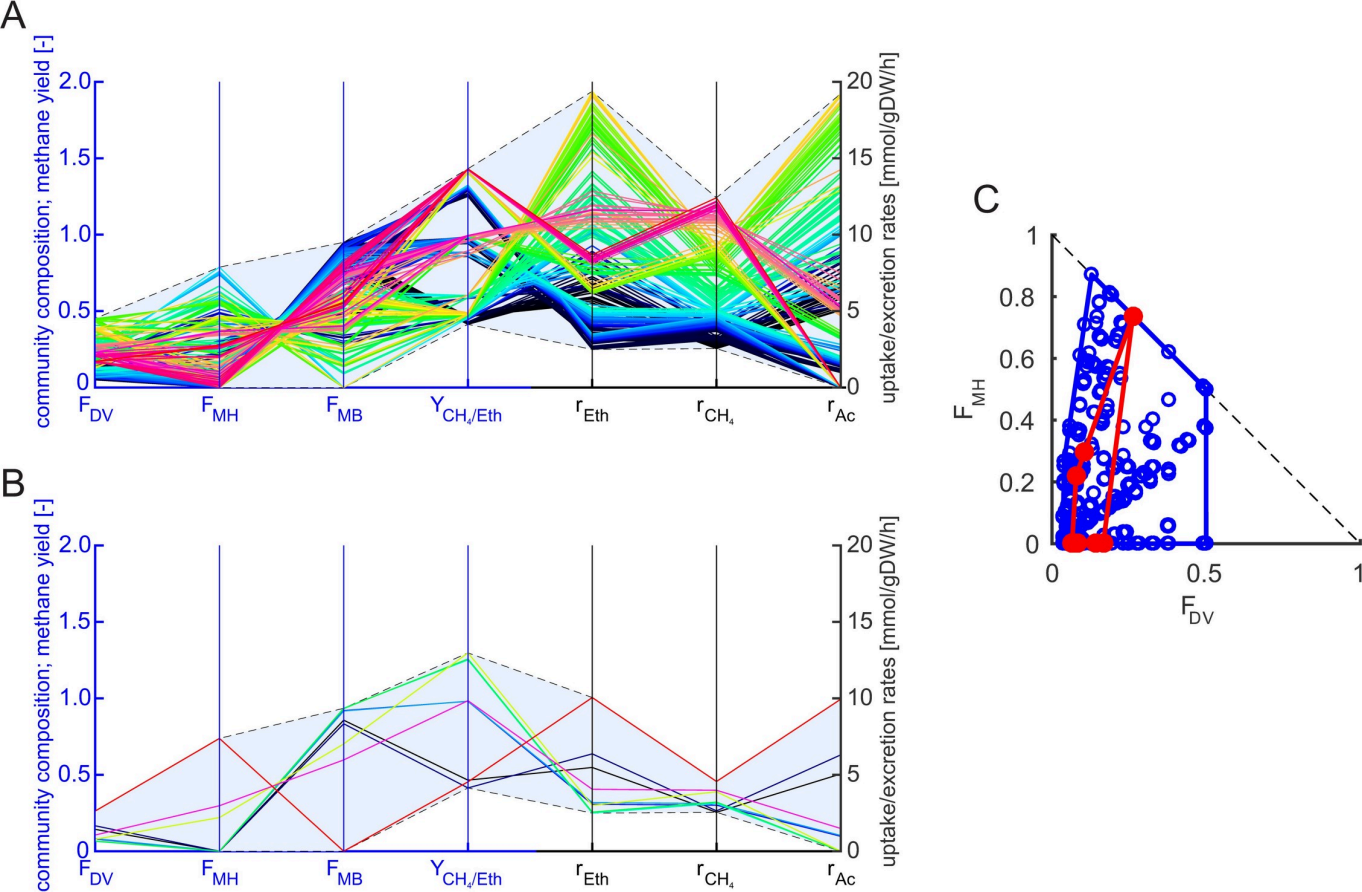
**Elementary flux vectors (EFVs) in the linearized full model (A) and in the reduced three-species community model (B) and their projection onto the fractional biomass abundances (C).** In all cases, ethanol served as substrate, the community growth rate was fixed to ***μ***_***c***_ = 0.0261 h^-1^ and acetate accumulation was allowed (cases in which no acetate accumulates correspond to EFVs with r_Ac_ = 0; compare also with S5 Text). In (A) and (B), the EFVs are projected onto their fractional biomass abundances (F_X_: fractional biomass abundance of species X), methane yields (Y_CH4/Eth_) and exchange rates (r_Eth_: ethanol uptake, r_CH4_: methane production, r_Ac_: acetate production) and are colored from red (highest methane excretion rate) via purple, orange, yellow, green, cyan, blue to black (lowest rate). The blue axes refer to biomass abundances and methane yield and the black axes to the exchange rates. The feasible ranges of compositions, exchange rates, and yields spanned by the EFVs are indicated by a blue area in (A) and (B). The 2D-plot in (C) shows the EFVs of the linearized full model (blue) and of the reduced model (red) projected onto the fractional biomass abundances of *D*. *vulgaris* (F_DV_) and *M*. *hungatei* (F_MH_)). The abundance of *M*. *barkeri* (F_MB_) follows from F_DV_+F_MH_+F_MB_ = 1.

Next, we derived the linearized full model from the bilinear model by fixing the community growth rate (see Methods). We used an iterative approach for fast identification of the maximum community growth rate *μ*_*c*,*max*_ via a series of linear optimizations with different (fixed) *μ*_*c*_ (see Methods). The maximum community growth rate for growth on ethanol predicted with the linearized model is 0.052 h^-1^ and thus, as expected, identical to the result retrieved with the bilinear model. We then fixed the community growth rate to four different values corresponding to 99%, 50%, 5% and 0% of *μ*_*c*,*max*_ and computed the feasible ranges of fractional abundances and exchange rates (via flux variability analysis with linear optimizations) as well as for methane yield (via linear-fractional optimization; see [43]), again, for the two scenarios with and without acetate accumulation (S5 Text). As expected, the predicted ranges are consistent with (lie always within the feasible ranges of) the bilinear model. Due to the fixed growth rates, the predicted ranges are significantly smaller than in the bilinear model (where ranges where computed for all feasible growth rates), however, they are partially still relatively large and thus not very conclusive, especially for exchange rates (S5 Text). The blue area in Fig 3A illustrates predicted feasible ranges for community compositions, yields and exchange rates in the full linearized model for a fixed *μ*_*c*_ = 0.026 h^-1^ (corresponding to 50% of *μ*_*max*_).

While the min/max ranges for fractional organism abundances and exchange rates can be calculated in the bilinear as well as in the linearized full model, interdependencies between these abundances are not immediately obvious. To analyze the space of possible community compositions more thoroughly, a sampling approach could be used where the *F*_*i*_ for one (or several) organism(s) is fixed and the influence on the remaining *F*_*i*_ is then investigated (see, for example, [15] and [16]). However, even in medium-scale community models, the computational costs for such a sampling approach become quickly prohibitive when sampling along the abundances of more than four or five species, even in the linearized model. Here, an analysis of elementary flux vectors (EFVs; [20]; see also Methods) can deliver useful insights. In the three-species linearized full model with fixed *μ*_*c*_ = 0.026 h^-1^ we computed these characteristic vectors yielding 13,574 EFVs (Fig 3A and 3C). The resulting ranges for **F** are the same as obtained by linear optimization but more subtle dependencies are uncovered by the EFVs. Each EFV contains a community composition and corresponding flux rates for all reactions in the model (Fig 3A shows the fractional abundances and community exchange rates for all EFVs, whereas Fig 3C shows a projection of the EFVs onto their fractional abundances). This enables us to immediately identify and characterize community compositions and flux distributions which have high CH_4_ production rates or yields and which reactions or pathways are essential for these solutions. In our example, *M*. *barkeri* becomes essential if acetate does not accumulate in the medium or if no external CO_2_ is supplied (see also S5 Text). Additionally, all EFVs with a methane yield above 0.5 require *M*. *barkeri* and the acetoclastic methanogenesis pathway and the highest methane yield is reached with high but not with the highest abundance of *M*. *barkeri*. Acetate produced by *D*. *vulgaris* is an additional substrate for methanogenesis and *M*. *barkeri* is the only organism present in the community, which is capable of converting acetate to methane and CO_2_. Such relationships, which are often non-obvious especially in more complex or less studied communities, can exhaustively be analyzed with EFVs. The information retrieved via EFVs can subsequently also be used to find interventions in the community to increase product yields (e.g. removal of certain species; knockout of certain pathways etc.).

#### Reduced model based on EFVs of single species models

Next, we constructed a reduced community model of the three-species community using our new reduction approach RedCom (see Methods and the example in S1 Text in the Supplements). RedCom is based on reduced single-species models constructed from the net conversions of EFVs that fulfill a minimality criterion in the exchange fluxes. Solutions with inefficient substrate use are discarded by this criterion which also ensures that flux vectors with suboptimal biomass yield for a given substrate and product combination are excluded avoiding solutions in the community model where a species altruistically synthesizes large amounts of products (instead of biomass) required by other species. The reduced community model is constructed for a particular *μ*_*c*_ and thus linear.

Using the iterative approach also employed in the full linearized model, we first determined the maximum community growth rate *μ*_*c*_ and found that it is, as expected, the same as in the bilinear and linearized full model (0.052 h^-1^). As for the linearized full model, we computed then the feasible ranges of species abundances, exchange reactions and methane yield for four different *μ*_*c*_ for each of the two considered scenarios with allowed / not allowed accumulation of acetate. (S5 Text). Due to the exclusion of unrealistic solutions with low biomass yields in the single-species models, the reduced model has a smaller solution space compared to the linearized full model resulting in significantly smaller predicted ranges, especially for exchange rates. This can exemplarily be seen by comparing the blue areas in Fig 3A and 3B showing the predicted feasible ranges in the linearized full vs. the reduced model for the fixed *μ*_*c*_ = 0.026 h^-1^ (50% of *μ*_*c*,*max*_). For a more detailed analysis of the reduced model, we also computed the EFVs in the reduced model for a given *μ*_*c*_ = 0.026 h^-1^ yielding 12 EFVs, a tremendous reduction compared to 13,574 EFVs in the linearized model. The EFVs span the reduced solution space with narrower ranges especially for the exchange reactions (Fig 3B). In the biomass abundance diagram (Fig 3C), they span a smaller subset of the possible biomass compositions from the linearized model. In this region, all organisms use their substrates efficiently. In particular, solutions with larger fractions of *D*. *vulgaris* are excluded favoring solutions with a higher percentage of *M*. *barkeri*. The EFVs of the reduced model also reveal that solutions with high methane yields (green and yellow EFV in Fig 3B) have lower methane production rates whereas in the linearized full model solutions with high methane yields and rates exist which most likely stem from (unrealistic) substrate-wasting pathways of some organism(s) resulting in higher overall conversions.

#### Computation of EFVs in larger community models

As emphasized before and exemplified in Fig 3, EFVs are very useful to analyze the solution space of community models instead of focusing only on single flux ranges or fractional abundances. However, in general, computation of EFVs becomes quickly infeasible in the linearized full version of complex community models with a larger number of species. To test the limits of both approaches, we stepwise increased the number of organisms in the community model and computed EFVs first for the full linearized and then for the reduced model (which, again, is itself constructed from the net conversions of selected EFVs of the single-species models). While the reduced model approach enables us to compute and analyze EFVs even for the nine-species model (discussed below), the computation of EFVs was not possible in linearized full models of communities with five or more organisms (Table 2).

### A six-species ethanol-degrading community model and comparison with experimental data

We extended the three-species community model to a model with six of the nine model organisms by additionally integrating *A*. *woodii*, *P*. *freudenreichii*, and *S*. *fumaroxidans* (Tables 1 and 2). The three additional organisms were chosen according to their potential of being part in an ethanol-degrading community; they represent functional guilds that extend the capability of the three-species community investigated above by additional pathways for homoacetogenesis and propionate fermentation. Growth of the other (remaining) three organisms (CA, SW, EC; see Table 1) is not supported with ethanol as substrate and they have therefore not been included yet. Note that, at this initial point, no experimental data have been used yet to adjust the composition of the community model; this will later be done when including metaproteomic data from a concrete enrichment culture.

#### Full bilinear model

In a first step, we used again the bilinear model to compute the maximum community growth rate. The solver delivered 0.0087 h^-1^ which is significantly lower than the predicted rate in the three-species model. Since the latter is a particular solution of the six-species community (where the other three species have zero abundance) the maximum community growth rate should be at least as high as in the three-species model (in fact, the true maximum community growth rate is *μ*_*c*,*max*_ = 0.10 h^−1^; see below). We observed that maximizing the growth rate in the bilinear model yields different results depending on the initial flux distribution used by the solver. This is likely due to plateaus in the objective function and indicates potential problems when solving this large non-linear system. Different starting solutions (flux vectors) and/or other solvers must therefore be tested in the bilinear model to obtain predictions with high fidelity. In contrast, the predicted ranges for substrate uptake and product formation rates as well as for methane yield (S7 Text) appear reasonable but are rather large providing thus only some rough information about the orders of magnitude.

**Table 3:** Feasible ranges for exchange rates, methane yields and methane to CO_2_ ratio predicted by the linearized full model and the reduced model for the six-species community with ethanol as substrate. For comparison, experimental data from the enrichment culture for growth on ethanol (average of two experiments) are listed. In the simulations, ***μ***_***c***_ was fixed to the dilution rate of the experiments (0.001 h^-1^) and accumulation of organic acids was switched off (according to experimental data). A table with simulation results and experimental data for other dilution rates can be found in S7 Text. The six species in the model are: *P*. *freudenreichii*, *A*. *woodii*, *D*. *vulgaris*, *S*. *fumaroxidans*, *M*. *barkeri* and *M*. *hungatei*.

	Exchange rates [mmol/gDW_c_/h]			Product yields and ratios [mol/mol]	
	Ethanol	CO_2_	Methane	CH_4_:CO_2_	CH_4_:Ethanol
Full model	0.21–8.35	0.086–4.15	0.29–12.50	3.01–3.43	1.40–1.50
Reduced model	0.21–1.31	0.086–0.63	0.30–1.94	3.06–3.43	1.40–1.48
Experimental data	0.59	0.27	0.87	3.25	1.48

#### Experimental data of enrichment cultures

**Fig 4:**
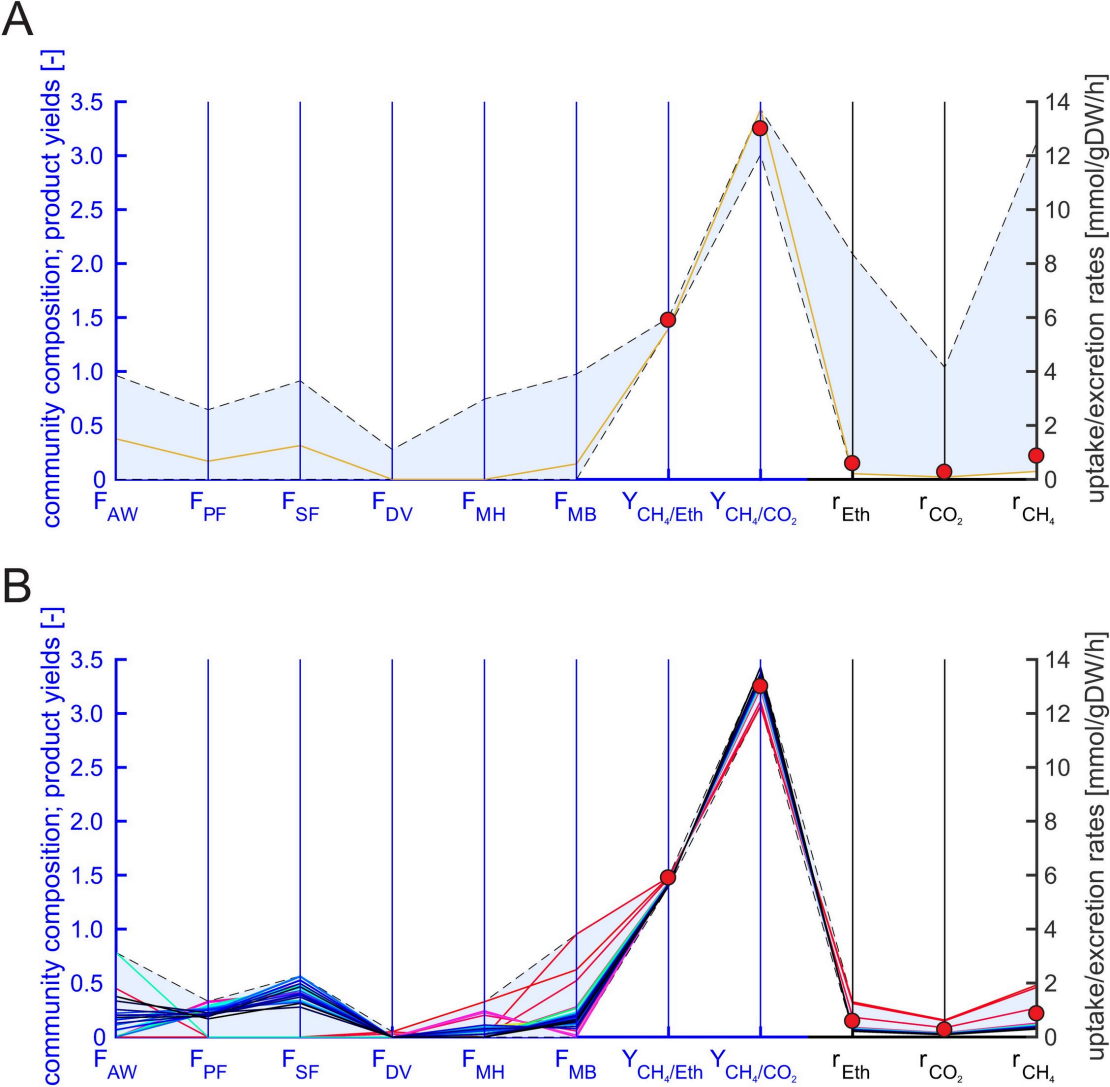
**Predicted community compositions (F_X_: fractional abundance of organism X; AW: *A*. *woodii*, PF: *P*. *freudenreichii*, SF: *S*. *wolfei*, DV: *D*. *vulgaris*, MH: *M*. *hungatei*, MB: *M*. *barkeri*), methane yield (Y_CH4/Eth_), ratio of methane to CO_2_ in the biogas (Y_CH4/CO2_), substrate uptake (r_Eth_) and product excretion rates (r_CO2_: CO_2_ excretion, r_CH4_: methane excretion) of the linearized full six-species model (A) and the reduced six-species model (B) for fixed ***μ***_***c***_ = 0.001 h^-1^.** The blue axes refer to biomass abundances, methane yield and methane to CO_2_ ratio whereas the black axes to the exchange rates. The ranges for the community composition in the full model were computed with flux variability analysis (FVA) (light blue area) whereas in the reduced model the EFVs were computed and plotted (solid lines) together with their convex hull (light blue area in (B)). The EFVs in (B) are colored from red (highest methane excretion rate) via orange, yellow, green, cyan and blue to black (lowest rate). In the linearized full model, we additionally minimized the ethanol uptake rate (corresponds to total biomass yield optimization), fixed the ethanol uptake rate to the computed minimum value and carried out another FVA for the remaining rates (orange area). In panel A as well as in panel B, experimental data (red circles) for a dilution rate of 0.001 h^-1^ are plotted (average values from two reactors; see Table **3**).

We carried out continuous cultivation experiments with enrichment cultures grown on ethanol (see Methods). Exchange rates calculated from measurement data are shown in Table 3 and Fig 4 for a dilution rate of *D* = *μ*_*c*_ = 0.001 h^-1^ (additional data for other dilution rates are provided in S7 Text). To compare the experimental results with the linearized full model and the reduced model (see below) we fixed *μ*_*c*_ to 0.001 h^-1^ and disabled accumulation of butyrate, propionate and acetate in the models because the concentration of these products were below the limit of quantification in the experiments.

#### Linearized full six-species model

As already mentioned above, EFV computation was not possible in the linearized full six-species model, but the ranges of substrate uptake and product formation rates as well as maximum yields could be computed for community growth rates fixed to the respective dilution rate with linear and linear-fractional optimization, respectively (Table 3, Fig 4A, S7 Text). The predicted ranges for the methane yield are very narrow. The experimental data do not always lie within but are very close to the predicted ranges thus largely confirming the community model (Table S7). Regarding the exchange rates, we found that the measured values lie all within the ranges of simulations. However, the ranges predicted by the linearized full model are very large and thus of low predictive power: maximum rates are partially more than 40 times higher than the minimum rates. These results can again be explained by many solutions with energy-wasting pathways where some organisms have a high substrate turnover with only little growth, which demands to take substrate efficiency into account. To focus on most relevant solutions in the full model, we may use flux balance analysis to identify solutions with high substrate efficiency (the same objective as used in the proposed RedCom approach to obtain the reduced model). However, maximizing the biomass yields of all organisms simultaneously is not trivial, especially if some organisms are capable of using multiple substrates [15]. One option is to maximize the total community biomass yield by minimizing the ethanol uptake rate for the community. We therefore first minimized the ethanol uptake rate (with fixed community growth rate), fixed the ethanol uptake flux to the minimum value and carried out a flux variability analysis for the remaining fluxes and yields (Fig 4A). Only four of the six organisms (*A*. *woodii*, *P*. *freudenreichii*, *S*. *fumaroxidans* and *M*. *barkeri*) are predicted to be present and no variability in exchange fluxes, yields and community composition was observed (hence, only a single optimal solution with maximal biomass yield exists). Further analysis of the flux ranges revealed that *P*. *freudenreichii* converts ethanol to propionate which is then consumed by *S*. *fumaroxidans* which produces acetate, hydrogen and CO_2_. All hydrogen is converted with CO_2_ to acetate by *A*. *woodii* and *M*. *barkeri* metabolizes the acetate to methane and CO_2_ by acetoclastic methanogenesis. The total biomass optimization always prefers organisms which have high biomass yields for the same substrate compared to other organisms for the chosen growth rate. Alternatives (in this case: different ethanol oxidizers, hydrogenotrophic methanogenesis) are excluded neglecting that other organisms may still have higher fitness, e.g. due to higher substrate affinities. In fact, in the metaproteomic data analysis described below, we will see that *D*. *vulgaris* and different methanogens are part of the studied enrichment culture while no homoacetogenesis or ethanol oxidation via propionate was observed.

#### Reduced six-species model

As before, we constructed the reduced six-species community model in which, in contrast to the full model, we could easily compute the (117) EFVs and from them the ranges of exchange rates and product yields (Table 3, S7 Text, and Fig 4B). The experimental data for the specific rates were again in the ranges predicted by the model but considerably smaller and thus more conclusive in the reduced model compared to the linearized full model. Hence, these results support the assumption of substrate-efficient flux distributions. The yield ranges are also very narrow and thus, similar to the full model, with high predictive power and in good agreement with measurements.

Again, the EFVs of the reduced model help to uncover more complex dependencies between fractional abundances and exchange rates in the reduced model and illustrate the enhanced predictive power compared to the linearized full model (Fig 4). While the latter predicts, for example, independent ranges (in blue in Fig 4A) for the community composition, the reduced model shows that e.g. *P*. *freudenreichii* and *S*. *fumaroxidans* always occur together in the community and that the methanogens are anti-correlated but at least one methanogen is always present (Fig 4B). Each EFV represents one possible (minimal) community phenotype with a characteristic community composition and substrate uptake rate as well as product yields and synthesis rates. Furthermore, any feasible community is a convex combination of these EFVs. Fig 4 also indicates again much tighter ranges in the exchange fluxes in the reduced model while the range of feasible methane yields is small in both the reduced and the full model. Contrary to the results from the total biomass optimization in the linearized full model (orange line in Fig 4A), we obtained several alternative community compositions and a range of possible exchange fluxes and yields. *A*. *woodii*, *P*. *freudenreichii* and *D*. *vulgaris* are alternative ethanol oxidizers in the community and both methanogens can be involved in methanogenesis. No organism is excluded based on its specific biomass yield since we selected net conversions from each organism with substrate efficiency at single-species level.

We noticed that the maximum community growth rate of *μ*_*c*,*max*_ = 0.10 h^−1^ calculated with both the reduced and the linearized full model was much higher than the highest dilution rate that supported a stable community in the experiments. Generally, *μ*_*c*,*max*_ is limited by the slowest essential organism in the community. The maximum substrate uptake rates (limiting the maximum growth rate) for these organisms were taken from single-species experiments. In these experiments, the growth conditions are usually optimized for that particular organism, which is not the case in the community experiments. In addition, substrates may differ between monocultures and enrichment cultures because growth on some substrates is only possible in the presence of other organisms (e.g. syntrophic acetate or ethanol oxidation) [44]. Furthermore, some substrate concentrations (e.g., hydrogen) in the experiments might not suffice for some organism to grow with *μ*_*max*_.

#### Model predictions are sensitive against ATP maintenance demand

**Fig 5:**
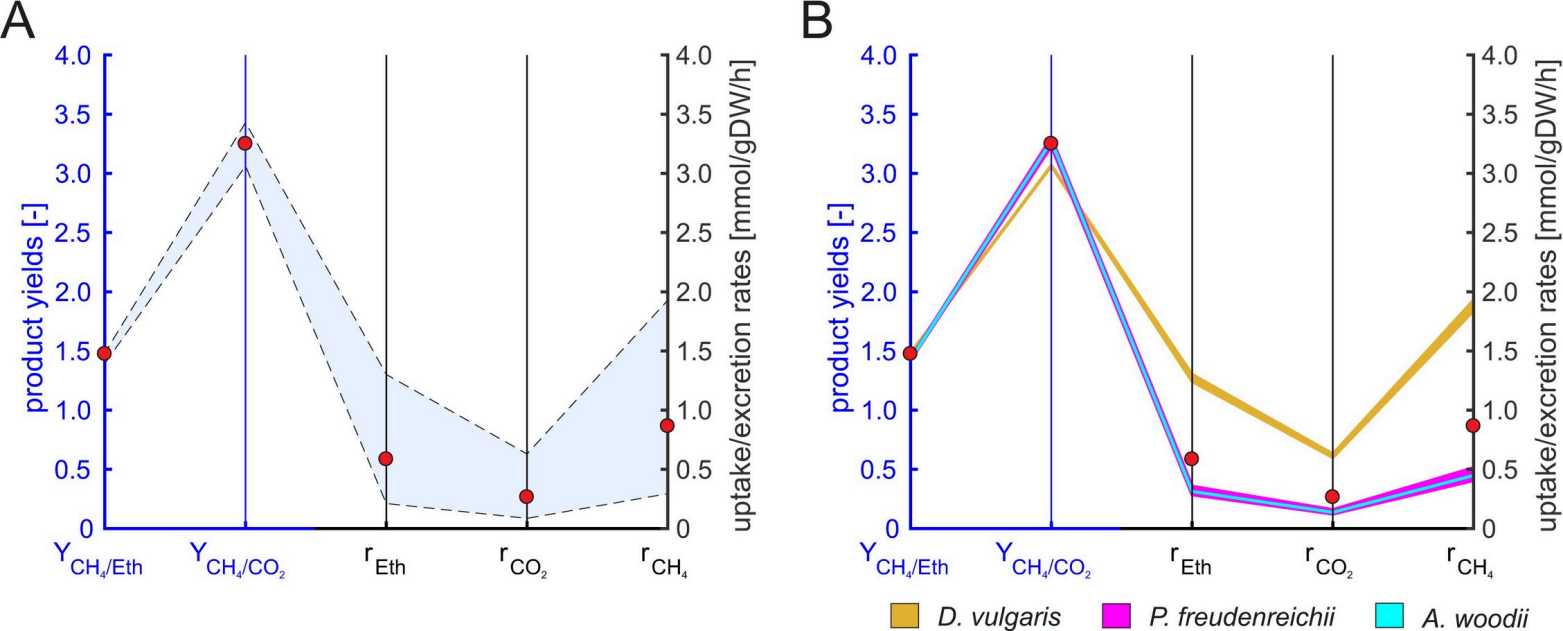
Influence of the different ethanol oxidizers in the reduced six-species model on predicted flux and yield ranges (colored areas) for the ethanol uptake rate (r_Eth_), CO_2_ excretion rate (r_CO2_), methane excretion rate (r_CH4_), methane to CO_2_ ratio (Y_CH4/CO2_) and methane yield (Y_CH4/Eth_). The blue axes refer to methane yield and methane to CO_2_ ratio whereas the black axes refer to the exchange rates. We first simulated all six organisms together (A) (cf. with Fig **4**B) and then the community with only one of the three different ethanol oxidizers active at a time (B): *D*. *vulgaris*: orange area; *P*. *freudenreichii* (with *S*. *fumaroxidans*, which consumes the propionate produced by *P*. *freudenreichii*): magenta area; *A*. *woodii*: cyan area. Note that the cyan and magenta regions are almost identical. The red circles show data from enrichment culture experiments (averaged values from two reactors for a dilution rate of 0.001 h^-1^; see Table **3**). The community growth rate in the model was set to the measured dilution rate of 0.001 h^-1^ with ethanol as the only substrate.

As mentioned earlier, while the measured exchange rates lie within the predicted ranges, these ranges are rather large, especially in the full but–partially—also in the reduced model. We therefore carried out further simulations with the reduced model to find the reasons for this variability. We first compared results using only one of the three different ethanol oxidizers at a time ((i) *D*. *vulgaris*, (ii) *A*. *woodii*, (iii) *P*. *freudenreichii* together with *S*. *fumaroxidans*, which can use propionate produced by *P*. *freudenreichii*; Fig 5). The variability in the exchange rates in these simulations is much smaller. The predicted exchange rates for simulations with *D*. *vulgaris* are considerably higher than the experimental data, with *A*. *woodii*, they are slightly above and with *P*. *freudenreichii* and *S*. *fumaroxidans* slightly lower than the experimental data (Fig 5B). One major difference between the ethanol oxidizers is the non-growth associated ATP maintenance demand. The maintenance coefficient for *D*. *vulgaris* estimated from literature was much higher than for the other organisms (Table 1), which may explain why the fractional abundance of *D*. *vulgaris* is predicted to be relatively low (Fig 4).

**Fig 6:**
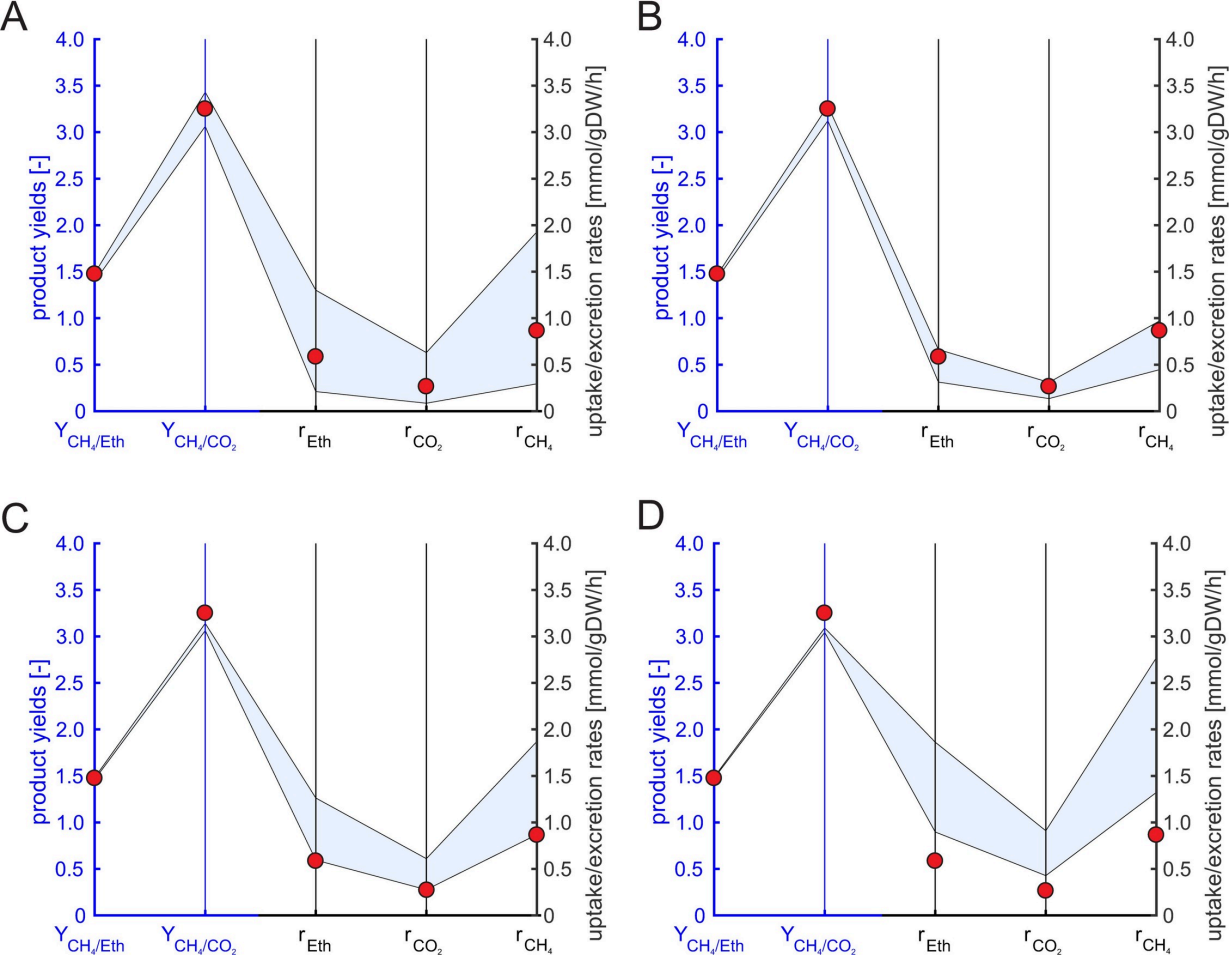
Influence of the maintenance coefficient on the predicted flux and yield ranges (light blue area) for the ethanol uptake rate (r_Eth_), CO_2_ excretion rate (r_CO2_), methane excretion rate (r_CH4_), methane to CO_2_ ratio (Y_CH4/CO2_) and methane yield (Y_CH4/Eth_). The blue axes refer to methane yield and methane to CO_2_ ratio whereas the black axes refer to the exchange rates. The maintenance coefficient was set to the original specific values (A) or to equal values of 1 (B), 2 (C) and 3 mmolATP/gDW/h (D) for all organisms. The red circles show data from enrichment culture experiments (averaged values of two reactors for a dilution rate of 0.001 h^-1^, see Table **3**). The community growth rate was fixed to 0.001 h^-1^ with ethanol as the only substrate.

In a second simulation, we therefore investigated the influence of the ATP maintenance coefficient (Fig 6). We used identical maintenance coefficients for all organisms and observed that this leads to smaller ranges for the predicted rates and yields. As expected, higher maintenance coefficients imply higher specific rates. Typically, the energy produced from substrate conversion is partly used for growth and partly for maintenance of the cell. The small growth rates measured in the experiments imply that a large portion of the substrate taken up is dedicated to maintenance processes. Out of the tested “averaged” maintenance coefficients, we found that 1 mmolATP/gDW/h for each organism reflected the experimental data best.

While the choice of the ethanol oxidizer as well as the maintenance coefficient clearly affected predicted uptake and production rates, we found that product yields and rate ratios barely changed and are thus less sensitive against uncertainties in these factors.

#### Mass spectrometric analysis of an ethanol enrichment culture and model refinement

**Fig 7:**
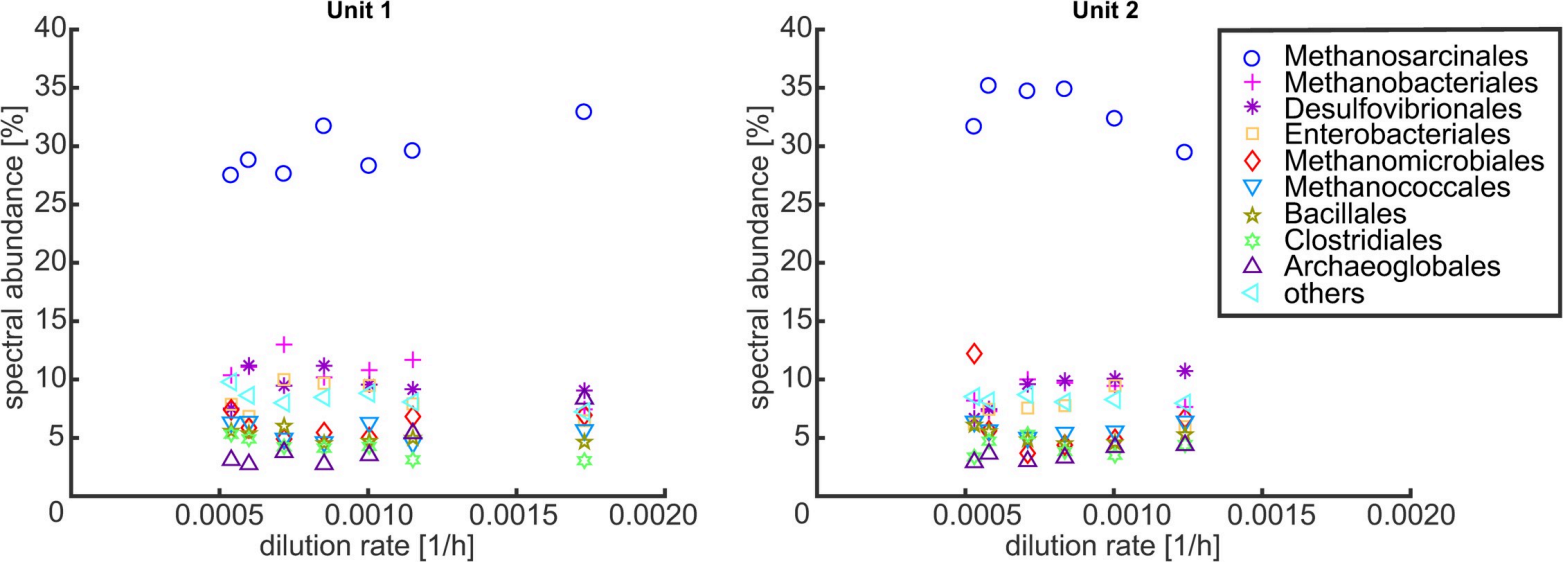
Spectral abundance for different taxonomic orders in the ethanol enrichment culture for different dilution rates. Spectral counts assigned to the superkingdom of virus or eukaryota and spectra not assigned to any taxonomic order were not considered; taxonomic orders which reached less than 5% in every sample were combined in ‘others’.

In addition to the abiotic data, we also analyzed metaproteomic data of the enrichment culture aiming for a taxonomic characterization and identification of active metabolic routes in the community (described in detail in S8 Text). The spectral abundances revealed that the most abundant taxonomic orders were Methanosarcinales, Methanobacteriales, Desulfovibrionales, Enterobacteriales, Methanomicrobiales, Methanococcales, Bacillales, Clostridiales and Archaeoglobales (Fig 7).

Additionally, we looked at the respective spectral counts for pathways potentially involved in the process of anaerobic digestion (S8 Text). High abundances could be found for enzymes for ethanol oxidation, methanogenesis, acetoclastic methanogenesis and acetate production while homoacetogenesis (*A*. *woodii*) and ethanol oxidation via propionate (*P*. *freudenreichii* and *S*. *fumaroxidans*) seem to be of minor relevance. As a consequence, mainly three out of the six (guild) organisms of the original six-species model are relevant to represent the ethanol enrichment culture. These organisms are *D*. *vulgaris*, *M*. *hungatei* and *M*. *barkeri*, which, accidentally, exactly correspond to the three-species community studied above. Accordingly, we adapted the reduced six-species model to reflect this composition (basically, the fractional biomass abundance of *A*. *woodii*, *P*. *freudenreichii* and *S*. *fumaroxidans* was set to zero). Further analysis also revealed that the majority of the spectral counts in Methanosarcinales belonged to the Methanosaeta species, which can only use acetate for methanogenesis. Since we have Methanosarcina instead of Methanosaeta as guild organism in our model, we also switched off the hydrogen uptake for *M*. *barkeri* to mimic Methanosaeta.

Including the respective constraints for the observations made in the six-species model (which then becomes effectively a three-species model) we can further confine the solution space of the reduced model (Table 4 and S8 Text). Due to the tighter constraints, the rates of specific ethanol uptake as well as of CO_2_ and methane production can now be determined exactly (no range) but the predictions appear, again, to be higher compared to the experimental data (Simulation 1 in Table 4). With the results from the six-species model, we may assume that the comparably very high maintenance coefficient of *D*. *vulgaris* (4.3 mmolATP/gDW/h) was overestimated. Indeed, using a common maintenance coefficient of 1 mmolATP/gDW/h for all three species (Simulation 2 in Table 4) the simulation results are closer to experimental data thus confirming a likely overestimation of the *D*. *vulgaris* maintenance coefficient. For Simulation 2, rates deviated less than 0.5 mmol/gDW/h and methane yields and methane to CO_2_ ratio less than 15% from the experimental data. Given a relatively high variation of the rate and yield measurements (cf. the two datasets for *μ*_*c*_ = 0.001 h^-1^ in S8 Text), a reasonable agreement between experimental data and predictions can thus be concluded.

**Table 4:** Feasible ranges for exchange rates, methane yields and methane to CO_2_ ratio for two simulations with different maintenance coefficients in the six-species model constrained by metaproteomic data (effectively, only the three species *D*. *vulgaris* (DV), *M*. *barkeri* (MB) and *M*. *hungatei* (MH) remain active). Simulation 1: original maintenance coefficients, simulation 2: maintenance coefficients of 1 mmol_ATP_/gDW/h for all species. Additionally, experimental data from the enrichment culture for growth on ethanol (average of two experiments; see S8 Text) are listed. In the simulations, ***μ***_***c***_ was fixed to the dilution rate of the experiments (0.001 h^-1^). Accumulation of organic acids was switched off (according to experimental data). A table with simulation results and experimental data for other dilution rates can be found in S8 Text.

	Exchange rates [mmol/gDW_c_/h]			Product yields and ratios [mol/mol]		Community composition		
	Ethanol	CO_2_	Methane	CH_4_:CO_2_	CH_4_:Ethanol	F_DV_	F_MH_	F_MB_
Simulation 1	1.30	0.63	1.93	3.06	1.48	0.05	0.33	0.62
Simulation 2	0.66	0.31	0.97	3.13	1.46	0.10	0.15	0.75
Experimental data	0.59	0.27	0.87	3.25	1.48	0.26	0.30	0.44

The model predicts a community composition of 62% Methanosarcinales, 33% hydrogenotrophic methanogens and 5% Desulfovibrionales when we use the original maintenance coefficients (Simulation 1) and 75% Methanosarcinales, 15% hydrogenotrophic methanogens and 10% of Desulfovibrionales if we use maintenance coefficients of 1 mmolATP/gDW/h for all three organisms (Simulation 2). The spectral abundance of the three taxonomic groups in the experiment was 44% of Methanosarcinales, 30% of the hydrogenotrophic and formate using methanogens and 26% of Desulfovibrionales (Table 4). Hence, simulations correctly predict the dominance of archaea and Methanosarcinales. However, the calculated percentage of Methanosarcinales was considerably higher than indicated by the spectral counts. One reason might be that we used a Methanosarcina species as a model organism whereas the experimental data suggest Methanosaeta species for acetoclastic methanogenesis. Methanosaeta has a lower ATP yield per acetate and therefore, also a lower biomass yield compared to Methanosarcina, which could explain this discrepancy. Additionally, many spectra could not be assigned to any of the above mentioned taxonomic orders leading to relatively large uncertainties in these results.

### Nine-species community model

We finally simulated a community capable of growth on glucose. Here, all of our nine guild organisms can potentially be involved in the process and are thus part of the community model (Tables 1 and 2). In addition to the six-species community studied above, this model included *E*. *coli*, *C*. *acetobutylicum* and *S*. *wolfei*. We first simulated the community with the bilinear model to predict the maximum community growth rate as well as ranges for substrate uptake, product excretion, biogas composition and methane yield (Table 5). As already observed for the six-species model, a reliable prediction for *μ*_*c*,*max*_ was thus not possible with this model (with the iterative approach in the linearized models we found that *μ*_*c*,*max*_ = 0.23 h^-1^) In contrast, the predicted ranges for reaction rates and yields seem reasonable.

**Table 5:** Simulation results of the nine-species community model (bilinear and linearized full model and reduced model). The minimum and maximum substrate uptake and product formation rates were computed with nonlinear optimization (bilinear model), FVA (linearized full model) or EFV analysis (reduced model). The community growth rate was set to 0.00067 h^-1^ (linearized full model, reduced model), which was the dilution rate used in an experiment with an enrichment culture grown on glucose-cellulose medium. The experimental data of two experiments and their average is also listed in the table. The nine species in the model are: *E*. *coli*, *C*. *acetobutylicum*, *S*. *wolfei*, *P*. *freudenreichii*, *A*. *wooddii*, *D*. *vulgaris*, *S*. *fumaroxidans*, *M*. *barkeri* and *M*. *hungatei* (see also Tables **1** and **2**).

	Community growth rate [h^-1^]	Exchange rates [mmol/gDW/h]			Product yields / rate ratios [mol/mol]	
		Glucose	CO_2_	CH_4_	CH_4_:CO_2_	CH_4_: Glucose
Bilinear model (feasible ranges)	Range could not be determined reliably	0.059–4.07	0.18–10.95	0.17–10.95	0.94–1.00	1.91–3.00
Experiment 1	0.00067	0.042	0.074	0.11	1.44	2.56
Experiment 2	0.00067	0.074	0.14	0.19	1.38	2.64
Average of both experiments	0.00067	0.057	0.11	0.15	1.40	2.61
Linearized full model	0.00067 (fixed to dilution rate)	0.069–3.65	0.19–10.95	0.19–10.94	0.99–1.00	2.79–3.00
Reduced model	0.00067 (fixed to dilution rate)	0.069–0.48	0.19–1.41	0.19–1.41	0.99–1.00	2.78–2.97

**Fig 8:**
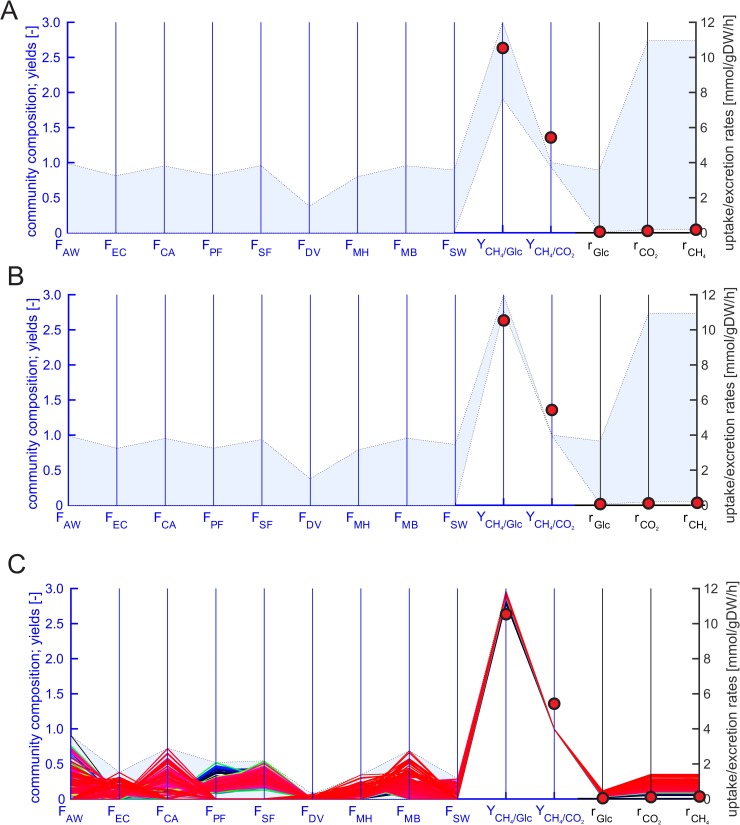
**Community composition (F: fractional biomass abundance, AW: *A*. *woodii*, EC: *E*. *coli*, CA: *C*. *acetobutylicum*, PF: *P*. *freudenreichii*, SF: *S*. *fumaroxidans*, DV: *D*. *vulgaris*, MH: *M*. *hungatei*, MB: *M*. *barkeri*, SW: *S*. *wolfei*), methane yield (Y_CH4/Glc_), methane to CO_2_ ratio: (Y_CH4/CO2_)) and metabolic rates (r_Glc_: glucose uptake, r_CO2_: CO_2_ excretion, r_CH4_: methane excretion) for the bilinear model (A), linearized full model (B) and the reduced model (C).** The blue axes correspond to the biomass abundances, methane yield and methane to CO_2_ ratio whereas the black axes to the exchange rates. In the bilinear model, the ranges (light blue area) were obtained by nonlinear optimization, in the linear model with FVA. For the reduced model, we computed the EFVs (solid colored lines, colored from red (highest methane production rate) via orange, yellow, green, cyan and blue to black (lowest methane production rate). Additionally, the experimental data (average from two reactors) are plotted (red circles) in all three cases. In the linear (full and reduced) models, *μ*_*c*_ was set to 0.0067 h^-1^ corresponding to the dilution rate of the experiment.

We then compared predictions of the linearized full model and the reduced model with experimental data (Table 5) from an enrichment culture grown on glucose-cellulose medium ([31]; see also Methods). Data were available for two duplicate experiments with identical dilution rate. Since hydrolysis of cellulose is not included in the model, we used glucose as a starting point and assumed that cellulose is converted to glucose by hydrolytic enzymes. We set the community growth rate to 0.00067 h^-1^, which corresponds to the dilution rate of the experiment and derived the corresponding linearized full community model and the reduced community model. EFV computation was possible with the reduced model (213689 EFVs) but not with the full model where we computed only ranges for biomass compositions, exchange rates, and methane yield via flux variability analysis (Table 5 and Fig 8).

Confirming findings from the three- and six-species models, we observed that the predicted ranges, especially of exchange rates and community compositions, are again considerably smaller in the reduced model compared to the linearized full model. In fact, the calculated ranges of exchange rates of the linearized full model are almost identical to the ones from the bilinear model, although the latter did not consider a fixed growth rate. The measured exchange rates were only slightly smaller than the minimum rates predicted by the models. The predicted ranges of the reduced model lie on the lower end of the range of the linearized full model and are thus closer to the experimental data indicating that the organisms use their substrate efficiently as assumed by our model reduction approach (Table 5 and Fig 8). The slight overestimation of the rates could again be a consequence of overestimating maintenance coefficients or an underestimation of ATP yields in the models. Furthermore, we noticed a relatively high variance of the measurements for the exchange rates which may partially explain deviations between data and model predictions. We also measured higher methane to CO_2_ ratios and lower methane yields than predicted by the models. Typically, we would expect a ratio of 1 methane to one CO_2_ for carbohydrates like glucose. However, some of the released CO_2_ might have been lost due to its better solubility in water (compared to methane).

## Discussion

Microbial communities are of major importance for health, nature, and biotechnological applications. Constraint-based stoichiometric modeling helps to obtain a better understanding of interrelationships in these communities and to make quantitative predictions. However, compared to classical constraint-based modeling of single-species metabolic networks, analysis of community models based on the favored concept of balanced growth is hampered by four major technical difficulties:

1. In contrast to linear single-species metabolic models, community models are bilinear due to the necessary explicit consideration of the community composition. In order to solve optimization problems in these models, one either has to rely on non-linear solvers, which may deliver only sub-optimal solutions, or one has to linearize the model, which requires to fix either the fractional abundances of the involved species or the community growth rate. While the latter case is generally easier to handle, multiple optimizations have to be performed to identify, for example, the maximum community growth rate and associated community compositions.
2. Flux variability analysis (FVA) is an essential tool for analysis of community models, especially for computing feasible ranges of community compositions and exchange rates. While min/max values of single abundances can be computed easily, deeper insights in relationships between fractional abundances (and metabolic exchange rates) require an exhaustive scanning of the whole solution space, which, becomes quickly prohibitive for communities with more than five species due to combinatorial reasons.
3. The solution space of a community model often contains spurious solutions in which one species behaves altruistically by synthesizing large amounts of products required by another species (instead of synthesizing its own biomass). Such unrealistic solutions may occur even for community compositions allowing maximum community growth rate [15]. It is thus important to identify and remove such solutions from the solution space before starting detailed analyses.
4. Due to their combinatorial complexity, linearized community models are often not amenable to detailed pathway analysis based on elementary flux modes (EFMs) or elementary flux vectors (EFVs), even if the community model has been compiled from smaller single-species core models where the EFVs are actually computable (cf. Tables 1 and 2). As shown in this paper, EFV analysis of the community models, if feasible, is very useful yielding deeper insights than simple FVA and avoids, for example, the problems mentioned under point (2).

Our introduced RedCom approach, where reduced community models are constructed from net conversions of the linear single-species models, addresses three of the above four issues ((2)-(4)). Taffs et al. [10] also published an approach where EFMs (instead of EFVs) of single-species models were used as input for the community model (“nested pathway consortium analysis approach”). While the basic principle is the same, our RedCom approach uses EFVs instead of EFMs which is mandatory to guarantee balanced growth of the community and to allow the consideration of flux bounds, maintenance coefficients, and other inhomogeneous constraints. A necessary pre-processing step is the calculation of EFVs in the single-species models for the fixed community growth rate followed by the selection of relevant EFVs projected onto their exchange fluxes. Different optimization or selection criteria can be used for selecting the relevant single-species behaviors. We decided to use all EFVs representing minimal conversions of exchange metabolites, which, as one particular advantage, ensures exclusion of unrealistic (altruistic) community behaviors of the respective species (see point (3)). Dependent on the application, other criteria could be used as well. In the three-, six-, and nine-species community models considered herein, the RedCom approach led to community models with desired properties: the models (a) are much smaller than the full (linearized) models, (b) exclude many spurious solutions, and (c) are amenable for detailed EFV analysis enabling the extraction of many important features of the community while avoiding an elaborate scanning of the solution space. There are two potential disadvantages of the reduction approach. First, the reduced community model contains information on the exchange fluxes while the internal flux distributions are not visible. However, in most applications of community models, the focus is indeed on predictions on the exchange fluxes, product yields, and feasible community compositions, which can all be derived from the respective flux vector of the reduced model. Furthermore, internal flux distributions of single-species could be “unpacked” from particular community net conversions whenever needed. A second potential disadvantage concerns the calculation of EFVs from the single-species models, which is usually not feasible if the latter are at genome-scale. However, with the typical application focus on exchange fluxes, single-species metabolic network models at the level of the central metabolism seem to be sufficient in many cases. Third, since the reduced community model requires eventually only the (minimal) net conversions of the single-species models, the (direct) calculation of elementary conversions might be a feasible approach even in genome-scale models [45].

We applied our RedCom approach to build community models of up to nine species relevant for the biogas process. We used a compartmented approach where each functional guild in anaerobic digestion is represented by a core model (central metabolism) of one organism. For the respective communities, we analyzed the maximum community growth rate and the feasible ranges of exchange rates, yields and fractional abundances of the involved species—with the bilinear as well as with the linearized and the reduced community model. Results were always consistent (in bilinear models, as long as the solver could reliably compute the respective minima and maxima). However, the reduced models obtained with the RedCom approach show a significantly narrower solution space by excluding solutions from the single-species models that are physiologically very unlikely resulting in more conclusive model predictions. While bilinear community models are usually linearized to make them amenable to constraint-based analysis techniques, we found that they can, in principle, be used to roughly gauge the community’s solution space. However, in larger models, some solutions found by the solver, especially for the determined maximum community growth rate, depended on starting values used for the solver pointing to potential issues with finding the global optimum in this non-linear optimization problem. Whenever the community growth rate can be fixed (e.g., to the maximum growth rate or to the dilution rate used in an experiment), the bilinear model becomes linear making its analysis and calculations simpler. With increasing numbers of organisms the computational costs, e.g. for an FVA-based scanning of the solution space, increase drastically also in the linearized (full) community model and EFVs could only be computed for models of up to four organisms. With the reduced community models, we were able to compute and analyze EFVs also for the largest community consisting of nine organisms.

In order to compare simulation results with experimental data from biogas communities and to investigate which solutions of the solution space are the most relevant in a concrete culture, we carried out experiments with an ethanol enrichment culture for different dilution rates. First, we compared experimental data with predicted ranges for specific substrate uptake and product formation rates as well as for biogas composition and methane yields obtained from the linearized full and the reduced six-species model. The predicted ranges of the specific rates covered the measured values but were very large and thus of low predictive power, especially in the full model. Confirming earlier findings [15], the maintenance coefficient of the different species has a tremendous impact on many properties of the community, especially on the predicted rates and community composition. Therefore, the maintenance coefficient should be determined as precisely as possible to obtain valid community models. As many other microbial communities, anaerobic digestion communities usually have a low growth rate implying that a relatively large fraction of the metabolism is devoted to maintenance processes. Generally, our results for the anaerobic digestion community indicate that the best agreement of model predictions and experimental data can be achieved when the maintenance coefficients of all species are approximately set to 1 mmol/(gDW∙h). In contrast to rates and community compositions, the predicted ranges for methane yields and biogas composition were much smaller and appeared to be less sensitive to the maintenance coefficients making these model predictions generally more reliable. In fact, methane yields and biogas compositions from the experiments were close to the predicted values for both the reduced and the full model. The predicted maximum growth rate of the full and the reduced six-species community model were identical but considerably higher than the maximum dilution rates that supported a stable process with the enrichment culture. Here, maximization of the community growth rate might not be a suitable objective function for communities in a realistic continuous process. In particular, maximum substrate uptake rates used in the models are usually derived from single-species cultures under their respective optimal conditions and it is likely that process conditions do not support optimal conditions and maximum growth rates for all organisms. The slowest (essential) species will then limit the overall community growth rate.

We used metaproteomic data from enrichment cultures for growth on ethanol to find out, which taxonomies and pathways were present in these cultures and to use this information to build a more constrained community model for this culture. The most abundant taxonomic orders identified in the experiments were *Methanosarcinales*, *Methanomicrobiales*, *Methanococcales*, *Methanobacteriales* and *Desulfovibrionales*, which correspond to the guilds represented by *M*. *barkeri*, *M*. *hungatei* and *D*. *vulgaris* in our six-species model. Furthermore, we found enzymes for ethanol oxidatation in *Desulfovibrionales*, acetoclastic and hydrogenotrophic methanogenesis in the archaeal superkingdom. There was little to no evidence for syntrophic acetate oxidation, homoacetogenesis and ethanol oxidation to propionate, which agrees well with the taxonomic analysis. In a last step, we used that information to further constrain the reduced six-species model and explored options to predict community compositions from the remaining solution space. The model predicted *M*. *barkeri* to be the dominant species in the community and *D*. *vulgaris* to be the least abundant organism. In fact, *Methanosarcinales* was also the taxonomic order with the highest spectral count abundance in the experiments while *D*. *vulgaris* had the lowest abundance confirming the model predictions. The experimental data also indicated that mainly *Methanosaeta* species were involved in acetoclastic methanogenesis. These organisms grow with lower biomass yield but higher substrate affinity compared to *Methanosarcina* species. Therefore, *Methanosaeta* should be added as a separate guild to the community model for future studies. Overall, to the best of our knowledge, the presented model-driven analysis of metaproteomic data from communities involved in anaerobic digestion is the biggest of its kind reported so far and demonstrates the high potential of a computer-aided approach to investigate properties and to assess experimental data of microbial communities.

## Supporting information

S1 Text Example for constructing and analyzing a reduced community model with the RedCom approach. (DOCX)

S2 Text Consistency of inhomogeneous constraints in single-species models with fluxes in the reduced community model. (DOCX)

S3 Text Flux constraints for the single-species models. (DOCX)

S4 Text Model validation. (DOCX)

S5 Text Simulation results for the three-species community for linearized full model, reduced model and bilinear model for different dilution rates. (DOCX)

S6 Text Detailed description of the experimental setup and analysis of the ethanol enrichment culture experiments. (DOCX)

S7 Text Simulation results for the six-species community model and experimental data from the ethanol enrichment culture. (DOCX)

S8 Text Metaproteomic analysis of ethanol enrichment culture and resulting model adjustments. (DOCX)

S1 Table Single species and reduced community models in text and SBML format. (XLSX)
