# Supplementary material for: RedCom: A strategy for reduced metabolic modeling of complex microbial communities and its application for analyzing experimental datasets from anaerobic digestion
Source: PLoS Comput Biol. 2019 Feb 1;15(2):e1006759. doi: 10.1371/journal.pcbi.1006759 (PMC6373973; doi:10.1371/journal.pcbi.1006759)
Supplement: S1 Text — (DOCX) [file pcbi.1006759.s001.docx]

# S1 Text: Example for constructing and analyzing a reduced community model with the RedCom approach

We use a simple example community to illustrate the construction of a reduced community model with the RedCom approach (see Methods in main text). We will also briefly show how the community models can be analyzed and how the special case of a community with no growth ($\mu_{c}$= 0 h^-1^) can be handled. The example community consists of three species (Figure A) and the structure of the community is shown in Figure B. The assumed inhomogeneous constraints (flux bounds) are listed in Table A. We consider the case of a fixed community growth rate of $\mu_{c}$=0.8 h^-1^.


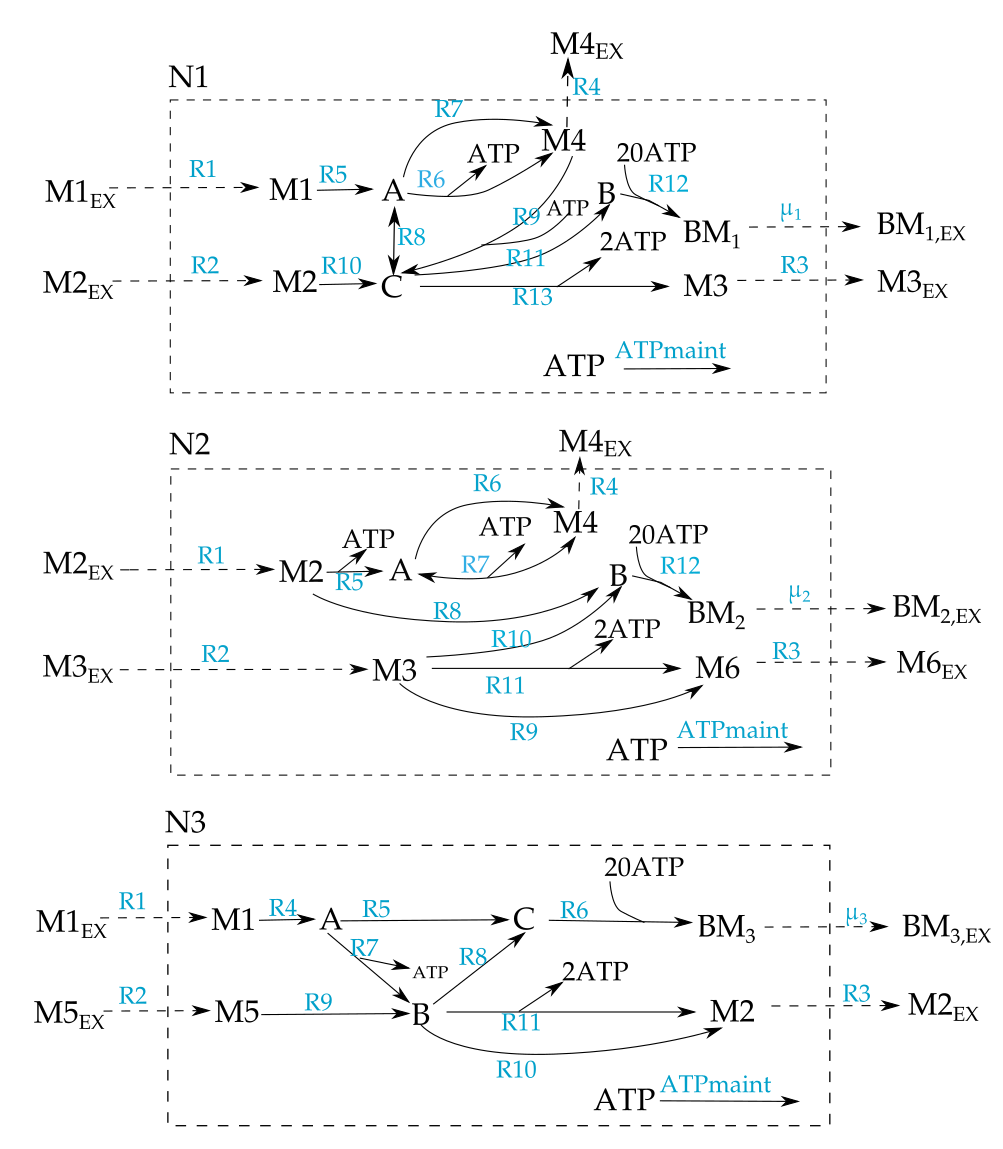


Figure A: Small example networks (N1, N2, N3) for three species. Dashed arrows: exchange reactions; index EX indicates external metabolites; BM_x_ corresponds to the biomass of species X.


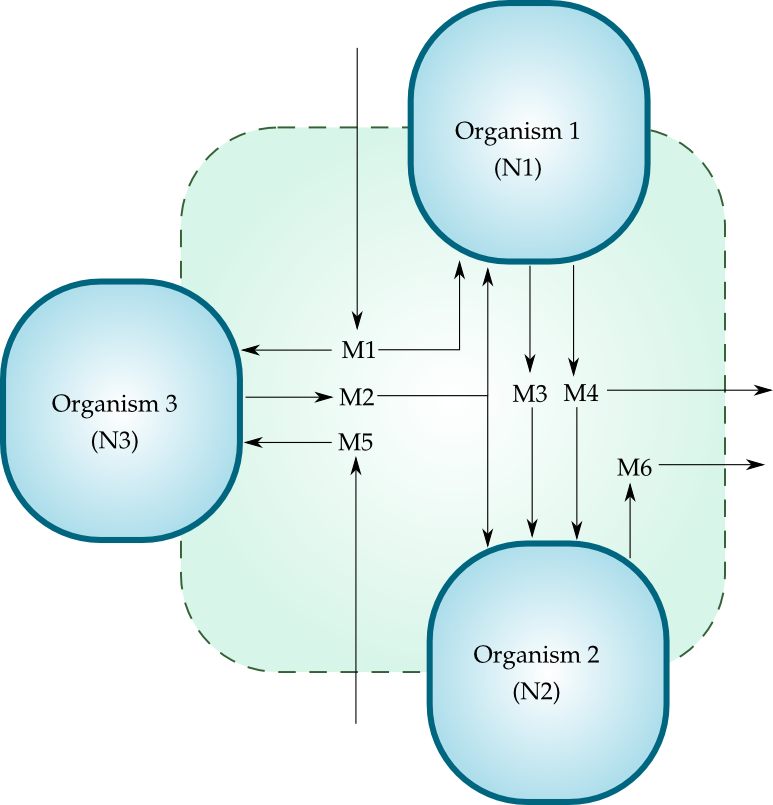


Figure B: Summary of the metabolite exchanges between the community members and the medium.

Table A: Inhomogeneous constraints for the example networks. The community growth rate (and thus the specific growth rates) were fixed to 0.8 h^-1^.

| **N1** | **N2** | **N3** |
| --- | --- | --- |
| R1 ≤ 40 mmol/g/h | R1 ≤ 40 mmol/g/h | R3 ≤ 50 mmol/g/h |
| R2 ≤ 30 mmol/g/h | R2 ≤ 30 mmol/g/h |  |
| R3 ≤ 50 mmol/g/h |  |  |
| ATPmaint ≥ 5 mmol/g/h | ATPmaint ≥ 2 mmol/g/h | ATPmaint ≥ 3 mmol/g/h |
| $\mu_{1}$ =0.8 h^-1^ | $\mu_{2}$=0.8 h^-1^ | $\mu_{3}$=0.8 h^-1^ |

We start by computing and selecting the net conversions for Network N1. The metabolites M1_EX_, M2_EX_, M3_EX_, M4_EX_ and BM_1,EX_ are defined as external metabolites and all other metabolites are internal (see Figure A). The reactions R1, R2, R3, R4 and $\mu_{1}$ involve external metabolites and therefore stand for interactions with the environment. These reactions are denoted here as exchange reactions.

In a first step, we compute all elementary flux vectors (EFVs) for N1 with the constraints given in Table A. The computation yields 44 bounded EFVs and one unbounded EFV (ray) representing an internal cycle consisting of R6, R8, R9). As explained in the main text, we do not consider rays in the following as they are thermodynamically infeasible and thus remove this EFV. Since we are interested in the net conversions (see main text), we project the EFVs onto the exchange reactions R1, R2, R3, R4, and growth ($\mu_{1})$:

| R1 | R2 | R3 | R4 | $\mu_{1}$ |  |
| --- | --- | --- | --- | --- | --- |
| 21.8 | 0 | 0 | 21 | 0.8 | EFV1 |
| 11.3 | 0 | 10.5 | 0 | 0.8 | EFV2 |
| 21 | 0.8 | 0 | 21 | 0.8 | EFV3 |
| 0 | 21.8 | 0 | 21 | 0.8 | EFV4 |
| 40 | 0 | 39.2 | 0 | 0.8 | EFV5 |
| 40 | 0 | 0 | 39.2 | 0.8 | EFV6 |
| 40 | 0.8 | 0 | 40 | 0.8 | EFV7 |
| 40 | 0 | 0 | 39.2 | 0.8 | EFV8 |
| 0 | 30 | 0 | 29.2 | 0.8 | EFV9 |
| 20.8 | 30 | 50 | 0 | 0.8 | EFV10 |
| 21.8 | 0 | 0 | 21 | 0.8 | EFV11 |
| 40 | 30 | 29.2 | 40 | 0.8 | EFV12 |
| 40 | 30 | 0 | 69.2 | 0.8 | EFV13 |
| 40 | 30 | 50 | 19.2 | 0.8 | EFV14 |
| 40 | 30 | 50 | 19.2 | 0.8 | EFV15 |
| 40 | 0 | 0 | 39.2 | 0.8 | EFV16 |
| 40 | 0 | 0 | 39.2 | 0.8 | EFV17 |
| 40 | 0.8 | 0 | 40 | 0.8 | EFV18 |
| 0 | 30 | 0 | 29.2 | 0.8 | EFV19 |
| 40 | 30 | 0 | 69.2 | 0.8 | EFV20 |
| 22.6 | 0 | 21.8 | 0 | 0.8 | EFV21 |
| 40 | 0 | 21.8 | 17.4 | 0.8 | EFV22 |
| 40 | 0 | 10.5 | 28.7 | 0.8 | EFV23 |
| 40 | 0 | 39.2 | 0 | 0.8 | EFV24 |
| 40 | 10.8 | 50 | 0 | 0.8 | EFV25 |
| 40 | 10.8 | 50 | 0 | 0.8 | EFV26 |
| 40 | 11.3 | 10.5 | 40 | 0.8 | EFV27 |
| 40 | 30 | 10.5 | 58.7 | 0.8 | EFV28 |
| 40 | 0 | 39.2 | 0 | 0.8 | EFV29 |
| 20.8 | 30 | 50 | 0 | 0.8 | EFV30 |
| 20.8 | 30 | 50 | 0 | 0.8 | EFV31 |
| 40 | 30 | 50 | 19.2 | 0.8 | EFV32 |
| 40 | 30 | 50 | 19.2 | 0.8 | EFV33 |
| 40 | 30 | 50 | 19.2 | 0.8 | EFV34 |
| 0 | 30 | 10.5 | 18.7 | 0.8 | EFV35 |
| 0 | 30 | 29.2 | 0 | 0.8 | EFV36 |
| 11.3 | 0 | 10.5 | 0 | 0.8 | EFV37 |
| 40 | 0 | 39.2 | 0 | 0.8 | EFV38 |
| 40 | 10.8 | 50 | 0 | 0.8 | EFV39 |
| 20.8 | 30 | 50 | 0 | 0.8 | EFV40 |
| 0 | 30 | 29.2 | 0 | 0.8 | EFV41 |
| 40 | 10.8 | 50 | 0 | 0.8 | EFV42 |
| 0 | 11.3 | 10.5 | 0 | 0.8 | EFV43 |
| 40 | 30 | 29.2 | 40 | 0.8 | EFV44 |

We then remove redundant projected EFVs (e.g., EFV1 is redundant with EFV11). In the next step, we remove EFVs that are non-optimal according to our optimality criterion (minimal conversions, see main text). For example, EFV6 is removed because all of its non-zero exchange rates are larger or equal to the ones in EFV11. We save the retained EFVs as columns in matrix $\mathbf{E}$:

|  |  |  |  |  |  | EFV43 | EFV4 | EFV37 | EFV11 |  |
| --- | --- | --- | --- | --- | --- | --- | --- | --- | --- | --- |
|  |  |  |  |  | R1 | 0 | 0 | 11.3 | 21.8 |  |
|  |  | $\mathbf{E}$**=** |  |  | R2 | 11.3 | 21.8 | 0 | 0 |  |
|  |  |  |  |  | R3 | 10.5 | 0 | 10.5 | 0 |  |
|  |  |  |  |  | R4 | 0 | 21 | 0 | 21 |  |
|  |  |  |  |  | $\mu_{1}$ | 0.8 | 0.8 | 0.8 | 0.8 |  |

We then construct the stoichiometric (sub)matrix $\mathbf{N}^{EX,1}$ which contains the external metabolites and the exchange reactions only:

|  |  | R1 | R2 | R3 | R4 | R5 | R6 | R7 | R9 | R10 | R11 | R12 | R13 | ATPmaint | $\mu$ |
| --- | --- | --- | --- | --- | --- | --- | --- | --- | --- | --- | --- | --- | --- | --- | --- |
|  | M1_EX_ | -1 | 0 | 0 | 0 | 0 | 0 | 0 | 0 | 0 | 0 | 0 | 0 | 0 | 0 |
|  | M2_EX_ | 0 | -1 | 0 | 0 | 0 | 0 | 0 | 0 | 0 | 0 | 0 | 0 | 0 | 0 |
|  | M3_EX_ | 0 | 0 | 1 | 0 | 0 | 0 | 0 | 0 | 0 | 0 | 0 | 0 | 0 | 0 |
|  | M4_EX_ | 0 | 0 | 0 | 1 | 0 | 0 | 0 | 0 | 0 | 0 | 0 | 0 | 0 | 0 |
|  | BM1_EX_ | 0 | 0 | 0 | 0 | 0 | 0 | 0 | 0 | 0 | 0 | 0 | 0 | 0 | 1 |
|  | M1 | 1 | 0 | 0 | 0 | -1 | 0 | 0 | 0 | 0 | 0 | 0 | 0 | 0 | 0 |
| $\mathbf{N}^{EX,1}$= | M2 | 0 | 1 | 0 | 0 | 0 | 0 | 0 | 0 | -1 | 0 | 0 | 0 | 0 | 0 |
|  | M3 | 0 | 0 | -1 | 0 | 0 | 0 | 0 | 0 | 0 | 0 | 0 | 1 | 0 | 0 |
|  | M4 | 0 | 0 | 0 | -1 | 0 | 1 | 1 | -1 | 0 | 0 | 0 | 0 | 0 | 0 |
|  | A | 0 | 0 | 0 | 0 | 1 | -1 | -1 | 0 | 0 | 0 | 0 | 0 | 0 | 0 |
|  | B | 0 | 0 | 0 | 0 | 0 | 0 | 0 | 0 | 0 | 1 | -1 | 0 | 0 | 0 |
|  | C | 0 | 0 | 0 | 0 | 0 | 0 | 0 | 1 | 1 | -1 | 0 | -1 | 0 | 0 |
|  | ATP | 0 | 0 | 0 | 0 | 0 | 1 | 0 | -1 | 0 | 0 | -20 | 2 | -1 | 0 |
|  | BM_1_ | 0 | 0 | 0 | 0 | 0 | 0 | 0 | 0 | 0 | 0 | 1 | 0 | 0 | -1 |

|  |  | R1 | R2 | R3 | R4 | $\mu_{1}$ |
| --- | --- | --- | --- | --- | --- | --- |
|  | M1_EX_ | -1 | 0 | 0 | 0 | 0 |
|  | M2_EX_ | 0 | -1 | 0 | 0 | 0 |
| $\mathbf{N}^{EX,1}$= | M3_EX_ | 0 | 0 | 1 | 0 | 0 |
|  | M4_EX_ | 0 | 0 | 0 | 1 | 0 |
|  | BM_EX_ | 0 | 0 | 0 | 0 | 1 |

This matrix $\mathbf{N}^{EX,1}$ is then multiplied with the selected and projected EFVs in $\mathbf{E}$. The resulting stoichiometric matrix $\mathbf{N}^{\mathrm{red},1}$ contains the net conversions of the selected EFVs:

|  |  | R^1,1^ | R^1,2^ | R^1,3^ | R^1,4^ |
| --- | --- | --- | --- | --- | --- |
|  | M1_EX_ | 0 | 0 | -11.3 | -21.8 |
|  | M2_EX_ | -11.3 | -21.8 | 0 | 0 |
| $\mathbf{N}^{\mathrm{red},1}\boldsymbol{=}\mathbf{N}^{EX,1}\mathbf{E}\boldsymbol{=}$ | M3_EX_ | 10.5 | 0 | 10.5 | 0 |
|  | M4_EX_ | 0 | 21 | 0 | 21 |
|  | BM_1_ | 0.8 | 0.8 | 0.8 | 0.8 |

We repeat those steps for the networks N2 and N3 and join the stoichiometric matrices of the organisms $\mathbf{N}^{\mathrm{red},i}$ into a stoichiometric matrix $\mathbf{N}^{c,red}$ for the community model:

|  |  | R^1,1^ | R^1,2^ | R^1,3^ | R^1,4^ | R^2,1^ | R^2,2^ | R^2,3^ | R^2,4^ | R^3,1^ | R^3,2^ |
| --- | --- | --- | --- | --- | --- | --- | --- | --- | --- | --- | --- |
|  | M1_EX_ | 0 | 0 | -11.3 | -21.8 | 0 | 0 | 0 | 0 | 0 | 0 |
|  | M2_EX_ | -11.3 | -21.8 | 0 | 0 | 0 | 0 | 0 | 0 | 0 | 0 |
|  | M3_EX_ | 10.5 | 0 | 10.5 | 0 | 0 | 0 | 0 | 0 | 0 | 0 |
|  | M4_EX_ | 0 | 21 | 0 | 21 | 0 | 0 | 0 | 0 | 0 | 0 |
|  | BM_1_ | 0.8 | 0.8 | 0.8 | 0.8 | 0 | 0 | 0 | 0 | 0 | 0 |
|  | M2_EX_ | 0 | 0 | 0 | 0 | 0 | -9.8 | -0.8 | -9 | 0 | 0 |
|  | M3_EX_ | 0 | 0 | 0 | 0 | -9.8 | 0 | -9 | -0.8 | 0 | 0 |
| $\mathbf{N}^{c,red}$ = | M4_EX_ | 0 | 0 | 0 | 0 | 0 | 9 | 0 | 9 | 0 | 0 |
|  | M6_EX_ | 0 | 0 | 0 | 0 | 9 | 0 | 9 | 0 | 0 | 0 |
|  | BM_2_ | 0 | 0 | 0 | 0 | 0.8 | 0.8 | 0.8 | 0.8 | 0 | 0 |
|  | M1_EX_ | 0 | 0 | 0 | 0 | 0 | 0 | 0 | 0 | 0 | -6.87 |
|  | M2_EX_ | 0 | 0 | 0 | 0 | 0 | 0 | 0 | 0 | 9.5 | 6.07 |
|  | M5_EX_ | 0 | 0 | 0 | 0 | 0 | 0 | 0 | 0 | -10.3 | 0 |
|  | BM_3_ | 0 | 0 | 0 | 0 | 0 | 0 | 0 | 0 | 0.8 | 0.8 |

The metabolites M1_EX_, M2_EX_, M3_EX_ and M4_EX_ occur in more than one network and need to be merged by summing up rows corresponding to the same metabolite:

|  |  | R^1,1^ | R^1,2^ | R^1,3^ | R^1,4^ | R^2,1^ | R^2,2^ | R^2,3^ | R^2,4^ | R^3,1^ | R^3,2^ |
| --- | --- | --- | --- | --- | --- | --- | --- | --- | --- | --- | --- |
|  | M1_EX_ | 0 | 0 | -11.3 | -21.8 | 0 | 0 | 0 | 0 | 0 | -6.87 |
|  | M2_EX_ | -11.3 | -21.8 | 0 | 0 | 0 | -9.8 | -0.8 | -9 | 9.5 | 6.07 |
|  | M3_EX_ | 10.5 | 0 | 10.5 | 0 | -9.8 | 0 | -9 | -0.8 | 0 | 0 |
| $\mathbf{N}^{c,red}$ = | M4_EX_ | 0 | 21 | 0 | 21 | 0 | 9 | 0 | 9 | 0 | 0 |
|  | M5_EX_ | 0 | 0 | 0 | 0 | 0 | 0 | 0 | 0 | -10.3 | 0 |
|  | M6_EX_ | 0 | 0 | 0 | 0 | 9 | 0 | 9 | 0 | 0 | 0 |
|  | BM_1_ | 0.8 | 0.8 | 0.8 | 0.8 | 0 | 0 | 0 | 0 | 0 | 0 |
|  | BM_2_ | 0 | 0 | 0 | 0 | 0.8 | 0.8 | 0.8 | 0.8 | 0 | 0 |
|  | BM_3_ | 0 | 0 | 0 | 0 | 0 | 0 | 0 | 0 | 0.8 | 0.8 |

Next, we add the reactions BM*_i_*🡪BMc (rate: $r_{{BM}_{i}{\to BM}_{c}}$), to merge the biomasses of the different species to the total biomass, and a reaction “exporting” the community biomass to the medium (with community growth rate $\mu_{c}$). In a final step, we add the sources and sinks for substrates and products for the community. We consider M1 and M5 as potential substrates for the community and M4 and M6 as products that can accumulate in the medium while M1, M2 and M3 are considered to not accumulate in the medium and have thus to be metabolized by the organisms. The exchange of metabolites between the organisms of the three networks is summarized in Figure B.

All metabolites are defined as internal metabolites in the obtained reduced community model and all reactions are irreversible. In addition, the (community) growth rate $\mu_{c}$ is fixed to the predefined value 0.8 h^-1^. The final stoichiometric matrix for the reduced community model reads (due to its size here shown in its transposed version):

|  |  | M1_EX_ | M2_EX_ | M3_EX_ | M4_EX_ | M5_EX_ | M6_EX_ | BM_1_ | BM_2_ | BM_3_ | BM_c_ |
| --- | --- | --- | --- | --- | --- | --- | --- | --- | --- | --- | --- |
|  | R^1,1^ | 0 | -11.3 | 10.5 | 0 | 0 | 0 | 0.8 | 0 | 0 | 0 |
|  | R^1,2^ | 0 | -21.8 | 0 | 21 | 0 | 0 | 0.8 | 0 | 0 | 0 |
|  | R^1,3^ | -11.3 | 0 | 10.5 | 0 | 0 | 0 | 0.8 | 0 | 0 | 0 |
|  | R^1,4^ | -21.8 | 0 | 0 | 21 | 0 | 0 | 0.8 | 0 | 0 | 0 |
|  | BM*_1_*🡪BMc | 0 | 0 | 0 | 0 | 0 | 0 | -1 | 0 | 0 | 1 |
|  | R^2,1^ | 0 | 0 | -9.8 | 0 | 0 | 9 | 0 | 0.8 | 0 | 0 |
|  | R^2,2^ | 0 | -9.8 | 0 | 9 | 0 | 0 | 0 | 0.8 | 0 | 0 |
|  | R^2,3^ | 0 | -0.8 | -9 | 0 | 0 | 9 | 0 | 0.8 | 0 | 0 |
|  | R^2,4^ | 0 | -9 | -0.8 | 9 | 0 | 0 | 0 | 0.8 | 0 | 0 |
|  | BM*_2_*🡪BMc | 0 | 0 | 0 | 0 | 0 | 0 | 0 | -1 | 0 | 1 |
| $\mathbf{N}^{c,red (T)}$= | R^3,1^ | 0 | 9.5 | 0 | 0 | -10.3 | 0 | 0 | 0 | 0.8 | 0 |
|  | R^3,2^ | -6.87 | 6.07 | 0 | 0 | 0 | 0 | 0 | 0 | 0.8 | 0 |
|  | BM*_3_*🡪BMc | 0 | 0 | 0 | 0 | 0 | 0 | 0 | 0 | -1 | 1 |
|  | $\mu_{c}$ | 0 | 0 | 0 | 0 | 0 | 0 | 0 | 0 | 0 | -1 |
|  | rM1in | 1 | 0 | 0 | 0 | 0 | 0 | 0 | 0 | 0 | 0 |
|  | rM2in | 0 | 1 | 0 | 0 | 0 | 0 | 0 | 0 | 0 | 0 |
|  | rM2ex | 0 | -1 | 0 | 0 | 0 | 0 | 0 | 0 | 0 | 0 |
|  | rM3in | 0 | 0 | 1 | 0 | 0 | 0 | 0 | 0 | 0 | 0 |
|  | rM3ex | 0 | 0 | -1 | 0 | 0 | 0 | 0 | 0 | 0 | 0 |
|  | rM4ex | 0 | 0 | 0 | -1 | 0 | 0 | 0 | 0 | 0 | 0 |
|  | rM6ex | 0 | 0 | 0 | 0 | 0 | -1 | 0 | 0 | 0 | 0 |
|  | rM5in | 0 | 0 | 0 | 0 | 1 | 0 | 0 | 0 | 0 | 0 |

## Simulations with the community model

For analysis of the community model, we consider two scenarios with M5 (scenario 1) or M1 (scenario 2) as substrate. We can apply standard methods of constraint-based modeling (e.g. flux balance analysis or EFV analysis) to analyze the community. The fractional biomass abundances $F_{i}$ can be calculated from the ratio of the rates $r_{\mathrm{BM}_{i}{\to BM}_{c}}$ and $\mu_{c}$. For our example, we computed the EFVs and plotted the fractional biomass abundances of N1 and N2 (Figure C). $F_{3}$ follows automatically: $F_{3}=1-F_{1}-F_{2}$. The results show which community members are exchangeable and which are essential under the given conditions.

| 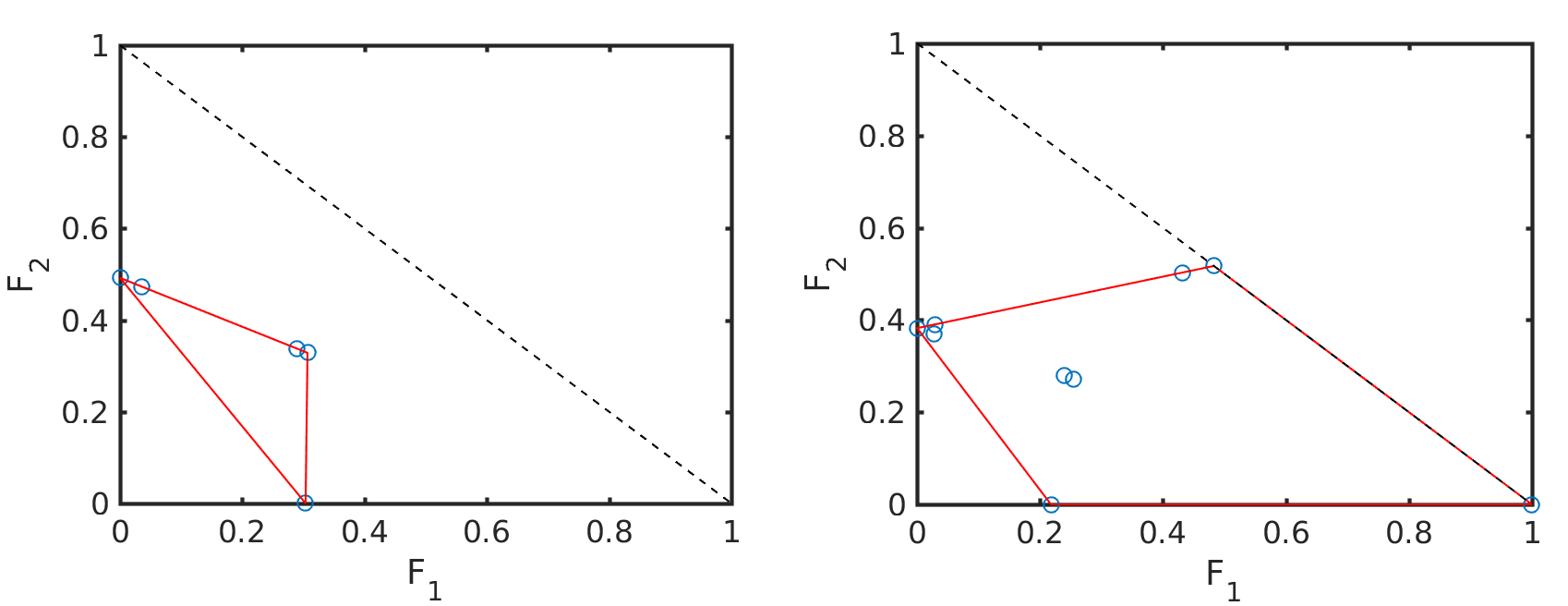  Figure C: Community composition calculated from the elementary flux vectors (EFVs) for scenario 1 (left) and scenario 2 (right). The community composition for each EFV is shown as a blue circle; the red line represents the convex hull which contains all feasible community compositions. Scenario 1 yielded 5 EFVs and while 9 EFVs result for scenario 2. Special case for non-growing communities ($\mu_{c}$=0) Above we described how to construct and analyze reduced community models with non-zero growth rates. For the case of non-growing communities ($\mu_{c}$=0) we need to change the structure of the community model in order to guarantee that the (specific) inhomogeneous constraints of the single-species models are full-filled (see S2 Text). Additionally, we cannot calculate the community composition from the biomass production rates (since no biomass is produced here) and therefore need to include them in the model in a different way.  For each organism *i*, we add an artificial pseudo metabolite $W_{i}$ which has a stoichiometric coefficient of +1 in each reaction of the stoichiometric matrix of the reduced model. Analogous to $r_{{BM}_{i}{\to BM}_{c}}$ in the standard reduced model, we add reactions consuming $W_{i}$ and producing $W_{c}$ (denoted as reactions $rF_{i}$): 1 $W_{i}$ 🡪 1 $W_{c}$ and analogous to $\mu_{c}$ we include a sink reaction for $W_{c}$ (${rW}_{ex}$). The rate for the latter reaction must be set to one for all simulations with the community model. The fractional biomass abundances for the organisms in flux vectors in this model correspond to the reaction rates of $rF_{i}$.  For our example the resulting (transposed) stoichiometric matrix $\mathbf{N}^{c,red}$for $\mu_{c}=0$ reads:   \|  \|  \| M1_EX_ \| M2_EX_ \| M3_EX_ \| M4_EX_ \| M5_EX_ \| M6_EX_ \| BM_1_ \| BM_2_ \| BM_3_ \| BM_c_ \| W*_1_* \| W*_2_* \| W*_3_* \| W*_c_* \| \| --- \| --- \| --- \| --- \| --- \| --- \| --- \| --- \| --- \| --- \| --- \| --- \| --- \| --- \| --- \| --- \| \|  \| R^1,1^ \| 0 \| -2.5 \| 2.5 \| 0 \| 0 \| 0 \| 0 \| 0 \| 0 \| 0 \| 1 \| 0 \| 0 \| 0 \| \|  \| R^1,2^ \| 0 \| -5 \| 0 \| 5 \| 0 \| 0 \| 0 \| 0 \| 0 \| 0 \| 1 \| 0 \| 0 \| 0 \| \|  \| R^1,3^ \| -2.5 \| 0 \| 2.5 \| 0 \| 0 \| 0 \| 0 \| 0 \| 0 \| 0 \| 1 \| 0 \| 0 \| 0 \| \|  \| R^1,4^ \| -5 \| 0 \| 0 \| 5 \| 0 \| 0 \| 0 \| 0 \| 0 \| 0 \| 1 \| 0 \| 0 \| 0 \| \|  \| rF*_1_* \| 0 \| 0 \| 0 \| 0 \| 0 \| 0 \| 0 \| 0 \| 0 \| 0 \| -1 \| 0 \| 0 \| 1 \| \|  \| BM_1_🡪BMc \| 0 \| 0 \| 0 \| 0 \| 0 \| 0 \| -1 \| 0 \| 0 \| 1 \| 0 \| 0 \| 0 \| 0 \| \|  \| R^2,1^ \| 0 \| 0 \| -1 \| 0 \| 0 \| 1 \| 0 \| 0 \| 0 \| 0 \| 0 \| 1 \| 0 \| 0 \| \|  \| R^2,2^ \| 0 \| -1 \| 0 \| 1 \| 0 \| 0 \| 0 \| 0 \| 0 \| 0 \| 0 \| 1 \| 0 \| 0 \| \|  \| rF*_2_* \| 0 \| 0 \| 0 \| 0 \| 0 \| 0 \| 0 \| 0 \| 0 \| 0 \| 0 \| -1 \| 0 \| 1 \| \|  \| BM_2_🡪BMc \| 0 \| 0 \| 0 \| 0 \| 0 \| 0 \| 0 \| -1 \| 0 \| 1 \| 0 \| 0 \| 0 \| 0 \| \|  \| R^3,1^ \| 0 \| 1.5 \| 0 \| 0 \| -1.5 \| 0 \| 0 \| 0 \| 0 \| 0 \| 0 \| 0 \| 1 \| 0 \| \|  \| R^3,2^ \| -1 \| 1 \| 0 \| 0 \| 0 \| 0 \| 0 \| 0 \| 0 \| 0 \| 0 \| 0 \| 1 \| 0 \| \| $\mathbf{N}^{c,red (T)}$= \| rF*_3_* \| 0 \| 0 \| 0 \| 0 \| 0 \| 0 \| 0 \| 0 \| 0 \| 0 \| 0 \| 0 \| -1 \| 1 \| \|  \| BM*_3_*🡪BMc \| 0 \| 0 \| 0 \| 0 \| 0 \| 0 \| 0 \| 0 \| -1 \| 1 \| 0 \| 0 \| 0 \| 0 \| \|  \| rM1in \| 1 \| 0 \| 0 \| 0 \| 0 \| 0 \| 0 \| 0 \| 0 \| 0 \| 0 \| 0 \| 0 \| 0 \| \|  \| rM2in \| 0 \| 1 \| 0 \| 0 \| 0 \| 0 \| 0 \| 0 \| 0 \| 0 \| 0 \| 0 \| 0 \| 0 \| \|  \| rM2ex \| 0 \| -1 \| 0 \| 0 \| 0 \|  \| 0 \| 0 \| 0 \| 0 \| 0 \| 0 \| 0 \| 0 \| \|  \| rM3in \| 0 \| 0 \| 1 \| 0 \| 0 \| 0 \| 0 \| 0 \| 0 \|  \| 0 \| 0 \| 0 \| 0 \| \|  \| rM3ex \| 0 \| 0 \| -1 \| 0 \| 0 \| 0 \| 0 \| 0 \| 0 \| 0 \| 0 \| 0 \| 0 \| 0 \| \|  \| rM4ex \| 0 \| 0 \| 0 \| -1 \| 0 \| 0 \| 0 \| 0 \| 0 \| 0 \| 0 \| 0 \| 0 \| 0 \| \|  \| rM5in \| 0 \| 0 \| 0 \| 0 \| 1 \| 0 \| 0 \| 0 \| 0 \| 0 \| 0 \| 0 \| 0 \| 0 \| \|  \| rM6ex \| 0 \| 0 \| 0 \| 0 \| 0 \| -1 \| 0 \| 0 \| 0 \| 0 \| 0 \| 0 \| 0 \| 0 \| \|  \| rW_EX_ \| 0 \| 0 \| 0 \| 0 \| 0 \| 0 \| 0 \| 0 \| 0 \| 0 \| 0 \| 0 \| 0 \| -1 \| \|  \| $\mu_{c}$ \| 0 \| 0 \| 0 \| 0 \| 0 \| 0 \| 0 \| 0 \| 0 \| -1 \| 0 \| 0 \| 0 \| 0 \| |
| --- | --- | --- | --- | --- | --- | --- | --- | --- | --- | --- | --- | --- | --- | --- | --- | --- | --- | --- | --- | --- | --- | --- | --- | --- | --- | --- | --- | --- | --- | --- | --- | --- | --- | --- | --- | --- | --- | --- | --- | --- | --- | --- | --- | --- | --- | --- | --- | --- | --- | --- | --- | --- | --- | --- | --- | --- | --- | --- | --- | --- | --- | --- | --- | --- | --- | --- | --- | --- | --- | --- | --- | --- | --- | --- | --- | --- | --- | --- | --- | --- | --- | --- | --- | --- | --- | --- | --- | --- | --- | --- | --- | --- | --- | --- | --- | --- | --- | --- | --- | --- | --- | --- | --- | --- | --- | --- | --- | --- | --- | --- | --- | --- | --- | --- | --- | --- | --- | --- | --- | --- | --- | --- | --- | --- | --- | --- | --- | --- | --- | --- | --- | --- | --- | --- | --- | --- | --- | --- | --- | --- | --- | --- | --- | --- | --- | --- | --- | --- | --- | --- | --- | --- | --- | --- | --- | --- | --- | --- | --- | --- | --- | --- | --- | --- | --- | --- | --- | --- | --- | --- | --- | --- | --- | --- | --- | --- | --- | --- | --- | --- | --- | --- | --- | --- | --- | --- | --- | --- | --- | --- | --- | --- | --- | --- | --- | --- | --- | --- | --- | --- | --- | --- | --- | --- | --- | --- | --- | --- | --- | --- | --- | --- | --- | --- | --- | --- | --- | --- | --- | --- | --- | --- | --- | --- | --- | --- | --- | --- | --- | --- | --- | --- | --- | --- | --- | --- | --- | --- | --- | --- | --- | --- | --- | --- | --- | --- | --- | --- | --- | --- | --- | --- | --- | --- | --- | --- | --- | --- | --- | --- | --- | --- | --- | --- | --- | --- | --- | --- | --- | --- | --- | --- | --- | --- | --- | --- | --- | --- | --- | --- | --- | --- | --- | --- | --- | --- | --- | --- | --- | --- | --- | --- | --- | --- | --- | --- | --- | --- | --- | --- | --- | --- | --- | --- | --- | --- | --- | --- | --- | --- | --- | --- | --- | --- | --- | --- | --- | --- | --- | --- | --- | --- | --- | --- | --- | --- | --- | --- | --- | --- | --- | --- | --- | --- | --- | --- | --- | --- | --- | --- | --- | --- | --- | --- | --- | --- | --- | --- | --- | --- | --- | --- | --- | --- | --- | --- | --- | --- | --- | --- | --- | --- | --- | --- | --- | --- | --- | --- | --- | --- | --- | --- | --- | --- | --- | --- | --- | --- | --- | --- | --- | --- | --- | --- | --- | --- | --- | --- | --- | --- | --- | --- | --- | --- | --- | --- | --- | --- | --- | --- |

Note that, as demanded, none of the species-specific net conversions in $\mathbf{N}^{c,red}$ produce biomass of a single organism (coefficients for BM*_1_*, BM*_2_*, and BM*_3_* are all zero) implying that the community growth rate $\mu_{c}$ is zero.
