# Supplementary material for: RedCom: A strategy for reduced metabolic modeling of complex microbial communities and its application for analyzing experimental datasets from anaerobic digestion
Source: PLoS Comput Biol. 2019 Feb 1;15(2):e1006759. doi: 10.1371/journal.pcbi.1006759 (PMC6373973; doi:10.1371/journal.pcbi.1006759)
Supplement: S2 Text — (DOCX) [file pcbi.1006759.s002.docx]

# S2 Text: Consistency of inhomogeneous constraints in single-species models with fluxes in the reduced community model

The net conversions of each organism in the reduced community model must agree with the inhomogeneous constraints in the respective single-species model. For example, the specific substrate uptake rate of species *i* in the reduced community model must not exceed the specific upper bound for this uptake rate originally given in the single-species model. In the following we show that this holds true as long as the (community) growth rate fixed in the single-species models (prior to construction of the reduced model) as well as in the constructed reduced community model itself are identical.

As in the main text, for the (single-species) metabolic model of species *i* we assume that substrate uptake is limited and that any non-zero flux vector $\mathbf{r}$ requires some minimum substrate uptake (no internal cycles and thus no unbounded elementary flux vectors (EFVs); see main text). We know that the set of feasible flux vectors $\mathbf{r}$ in species *i* forms a flux polyhedron $\mathbf{P}_{i}$ which is generated by convex combinations of the unique set of bounded EFVs **e** (see also Methods in main text):

$\mathbf{P}_{i}=\{\mathbf{r}\in\left. \mathfrak{R}^{z} \right|\mathbf{r}=\sum_{k\in K} \gamma_{ik}\mathbf{e}^{ik} \gamma_{ik}\geq0, \sum_{k\in K} \gamma_{ik}=1\}.$ (1)

We here focus on the exchange reactions and thus assume that the flux vectors $\mathbf{r}$ and the EFVs are projected onto these $z$ exchange reactions. Furthermore, as described in the main text, we assume that only the selected (substrate-efficient) EFVs are effectively used in (1), that is, all other EFVs not fulfilling this minimality criterion are excluded (or, alternatively, have a zero weight of $\gamma_{ik}=0$).

According to the description in the main text and S1 Text, the solution space (polyhedron) of flux vectors in the reduced community model is given by:

$\mathbf{P}^{c,red}\boldsymbol{=\{}{\boldsymbol{r}^{c,red}\boldsymbol{|}\mathbf{N}^{c,red}}\mathbf{r}^{c,red}=\mathbf{0,} \mathbf{r}^{c,red}\boldsymbol{\geq0,} \mu_{c}=\mu_{c,fix}\}$ (2)

$\mathbf{r}^{c,red}$ is the reaction rate vector of the community model. With $\mathbf{N}^{c,red, i}$ we denote the sub-matrix (reactions) of $\mathbf{N}^{c,red}$ that stem from the net conversions of the relevant EFVs from species *i* and $\mathbf{r}^{c,red,i}$ is the respective sub-vector of the corresponding reaction rates of these net conversions. We now have to show that the rates in $\mathbf{r}^{c,red,i}$ (with units [mmol/gDW_c_/h]) from the community model refer to a specific rate vector $\mathbf{r}$ from the flux polyhedron $\mathbf{P}_{i}$ of the *i*-th species (with units [mmol/gDW_i_/h], i.e., to a specific rate vector $\mathbf{r}$ which can be generated by the relevant EFVs as shown in eq. (1). We know that $\mathbf{N}^{c,red,i}\boldsymbol{=}\mathbf{N}^{EX,i}\mathbf{E}^{i}$, where $\mathbf{E}^{i}$contains the bounded EFVs $\mathbf{e}^{ik}$ of species *i* (used in eq. (1)) as columns. With that we can conclude that the specific rates $\mathbf{r}$ in species *i* can be obtained from the community rates $\mathbf{r}^{c,red,i}$ via:

$\mathbf{r}=\mathbf{E}^{i}\mathbf{r}^{c,red,i}/F_{i}$. (3)

with $F_{i}$ being the fractional abundance of species $i$*.* Combining (1) and (3) we thus have to show that

$\mathbf{E}^{i}\mathbf{r}^{c,red,i}/F_{i} = \mathbf{E}^{i}\boldsymbol{\gamma}^{i}$ , $\gamma_{ik}\geq0, \sum_{k\in K} \gamma_{ik}=1$

where $\boldsymbol{\gamma}^{i}$ is a vector containing the weighting factors $\gamma_{ik}$ for the EFVs which must sum up to 1. This can be simplified to

$\mathbf{r}^{c,red,i} = F_{i}\boldsymbol{\gamma}^{i}$ , $\gamma_{ik}\geq0, \sum_{k\in K} \gamma_{ik}=1$

With that it follows that the sum of the components of vector $\mathbf{r}^{c,red,i}$ must sum up to $F_{i}$:

${\sum_{\boldsymbol{j}} \boldsymbol{r}_{j}^{c,red,i}} = F_{i}$ . (4)

We now look at the steady-state condition (2) and focus on the steady-state equation for the biomass of species $i$ (BM*_i_*):

${\mathbf{N}_{BMi}^{c,red}}\mathbf{r}^{c,red}\mathbf{=}0$**.**

$\mathbf{N}_{BMi}^{c,red}$is the row in $\mathbf{N}^{c,red}$ that corresponds to metabolite BM*_i_*. Due to the way the EFVs were computed in the single-species model prior construction of the reduced model (growth rate fixed to a given $\mu_{c,fix}$) all coefficients in $\mathbf{N}_{BMi}^{c,red}$ which come from the EFV net conversions of species *i* must have a value identical to the fixed growth rate $\mu_{c}$ except the (later included) reaction incorporating the biomass of species $i$ into the community biomass (with rate $r_{{BM}_{i}{\to BM}_{c}}$) whose coefficient is −1 (see also the example in S1 Text). All other coefficients in $\mathbf{N}_{BMi}^{c,red}$ stem from other species and must thus be zero for metabolite BM*_i_*. We can thus conclude

$${\boldsymbol{\mu}_{c}}\mathbf{r}^{c,red,i}\mathbf{=}r_{{BM}_{i}{\to BM}_{c}}$$

which we can transform to

$\sum_{\boldsymbol{j}} \boldsymbol{r}_{j}^{c,red,i}\mathbf{=}\frac{r_{{BM}_{i}{\to BM}_{c}}}{\mu_{c}}=F_{i}$.

We thus see that, as demanded by (4), the fluxes in $\mathbf{r}^{c,red,i}$ indeed sum up to $F_{i}$ and the fluxes in the community model are thus consistent with flux bounds in the single-species model as long as the fixed $\mu_{c}$ is larger than zero.

In the special case of $\mu_{c}=0 h^{-1}$, which was discussed under S1 Text, the artificial metabolites $W_{i}$ and $W_{c}$ would be analogous to BM*_i_* and BM_c_, and $rW_{ex}$ would be analogous to the community growth reaction (with rate $\mu_{c})$ and $rF_{i}$ analogous to the reaction incorporating BM*_i_* into the community biomass (rate $r_{{BM}_{i}{\to BM}_{c}}$). For the same arguments derived above, this construction ensures that the community model is in agreement with the specific flux bounds in this particular case.
