# Supplementary material for: RedCom: A strategy for reduced metabolic modeling of complex microbial communities and its application for analyzing experimental datasets from anaerobic digestion
Source: PLoS Comput Biol. 2019 Feb 1;15(2):e1006759. doi: 10.1371/journal.pcbi.1006759 (PMC6373973; doi:10.1371/journal.pcbi.1006759)
Supplement: S3 Text — (DOCX) [file pcbi.1006759.s003.docx]

# S3 Text: Flux constraints for the single-species models

We here give an overview of data used to estimate (inhomogeneous) flux constraints such as maintenance coefficients and substrate uptake limits (Table A). Since we used the single-organism models as representatives for functional guilds and data are not available for every organism, we also used data from related organisms. The maintenance coefficient is represented by an ATP sink in the model. In some references the maintenance coefficient is given as a specific substrate uptake or product formation rate. In that case we converted the maintenance coefficient by multiplying it with the maximal ATP yield for that substrate or product from the model. When different maintenance coefficients were reported for one organism, we used the average value.

To estimate the upper bound for substrate uptake limits we used the maintenance coefficient and $\mu_{max}$ for the respective substrate as a constraint in the model and minimized the substrate uptake rate. The resulting uptake rate was used as an upper bound. Some organisms utilize different substrates and in many cases experimental data are available for growth on one substrate at a time only. We assume that the maximum growth rates will not add up when several different substrates are available for one organism. Therefore, we additionally put an upper bound to $\mu$ which we set to the highest observed value.

The models of *D. vulgaris*, *M. hungatei* and *M. barkeri* were published before [1] and are therefore not described herein.

## E. coli

We used a previously published model of *E. coli* and assumed an upper bound of 18.5 mmol/gDW/h for glucose uptake under anaerobic conditions and the ATP maintenance coefficient was set to 3.15 mmol_ATP_/gDW/h.

## S. fumaroxidans

We used the maintenance coefficient of 0.14 mmolATP/gDW/h published by Hamilton et al. [2].

Scholten et al. [3] published $\mu_{max}$ values for growth in monoculture on propionate ($\mu_{max}$=0.000833 h^-1^) plus sulfate and for cocultures on propionate ($\mu_{max}$=0.00867 h^-1^) and propionate plus sulfate ($\mu_{max}$=0.00371 h^-1^). The propionate requirement to achieve the given growth rates in the model was 1.6 mmol/gDW/h in the coculture scenario without sulfate.

## C. acetobutylicum

We used the maintenance coefficient used in the genome-scale model published by Lee et al. [4] which was 1 mmol_ATP_/gDW/h. For growth on glucose a growth rate of 0.72 h-1 was observed [5] corresponding to a maximum glucose uptake rate of 12.75 mmol/gDW/h in our model.

## S. wolfei

We did not find any information on maintenance coefficients in *S. wolfei* and therefore used the same maintenance coefficient as in the *S. fumaroxidans* model (see above). For growth on butyrate in different cocultures $\mu_{max}$ values between 0.008 and 0.0185 h^-1^ have been reported [6]. In crotonate monoculture Beaty et al. reported a growth rate of 0.039 h^-1^ (with rumen fluid) and 0.023 h^-1^ on defined medium [7]. The highest $\mu_{max}$ values were taken to estimate the upper substrate uptake bounds of 4.25 mmol/gDW/h (butyrate) and 2.85 mmol/gDW/h (crotonate).

## A. woodii

Data for estimation of the inhomogeneous constraints in the *A. woodii* model are summarized in Table A. We used the highest observed growth rate of 0.167 h^-1^ as an upper bound for $\mu$. For the maintenance coefficient used the average of the observed which was 0.29 mmol_ATP_/g/h.

## P. freudenreichii

Zhang et al. [8] measured a maintenance coefficient of 0.76 mmolATP/gDW/h. Maximum growth rates on glucose, lactate and ethanol with the corresponding uptake rates in the model are summarized in Table A.

Table A: Summary of maximum growth rates and maintenance coefficients for different substrates and organisms from literature data. The table also includes corresponding minimum uptake rates to achieve those growth rates in the model. The values taken as constraints in the model are underlined.

| **Organism** | **Substrate** | **Observed** $\boldsymbol{\mu}_{\boldsymbol{max}}$ **[h^-1^]** | **Corresponding uptake limit in the model** | **Observed/ calculated maintenance coefficient [mmol_ATP_/g/h]** | **Reference** |
| --- | --- | --- | --- | --- | --- |
| *P. freudenreichii* | glucose (+CO_2_) | 0.13 | 3.3 | 0.76 | [8] |
|  | glucose | 0.11 | 2.8 |  |  |
|  |  | 0.16 | 4.0 | - | [9] |
|  | lactate | 0.1 | 11.0 | - |  |
|  | ethanol | 0.1 | 11.0 | - | [10] |
| *S. wolfei* | butyrate (coculture) | 0.008 | 2.07 | - | [6] |
|  |  | 0.0185 | 4.25 | - | [11] |
|  | crotonate | 0.039 (with rumen fluid) | 2.85 | - | [7] |
|  |  | 0.023 | 1.72 | - |  |
|  |  | 0.029 | 2.14 | - | [6] |
| *A. woodii* | glucose | 0.167 | 2.75 | - | [12] |
|  | ethanol + bicarbonate | 0.1 | 9 | - | [12] |
|  | lactate | 0.036 | 3 | 0.00864 | [13] |
|  | H_2_ | 0.024 | 19.5 | 0.044 | [13] |
|  | lactate + H_2_ | 0.043 | - | 0.17 | [13] |
|  | methanol + formate | 0.046 | - | 0.573 | [14] |
|  | methanol + CO_2_ | 0.162 | 18.1 | 0.35 | [14] |
|  | fructose + CO_2_ | 0.147 | 2.42 | - | [15] |
| *S. fumaroxidans* | Fumarate | 0.0138 | 1.32 | - | [2] |
|  | Propionate (coculture) | 0.00867 | 1.60 | - | [3] |
|  |  | 0.0092 | 1.68 | - | [2] |
|  | Propionate + sulfate | 0.00371 | 0.25 | - | [3] |
|  | Propionate + fumarate | 0.03 | - | - | [2] |

References

1. Koch S, Benndorf D, Fronk K, Reichl U, Klamt S. Predicting compositions of microbial communities from stoichiometric models with applications for the biogas process. Biotechnol Biofuels. 2016; 9: 17. doi: 10.1186/s13068-016-0429-x.

2. Hamilton JJ, Calixto Contreras M, Reed JL. Thermodynamics and H2 Transfer in a Methanogenic, Syntrophic Community. PLoS Comput Biol. 2015; 11: e1004364. doi: 10.1371/journal.pcbi.1004364.

3. Scholten JCM, Conrad R. Energetics of Syntrophic Propionate Oxidation in Defined Batch and Chemostat Cocultures. Appl. Environ. Microbiol. 2000; 66: 2934–2942. doi: 10.1128/AEM.66.7.2934-2942.2000.

4. Lee J, Yun H, Feist AM, Palsson BØ, Lee SY. Genome-scale reconstruction and in silico analysis of the Clostridium acetobutylicum ATCC 824 metabolic network. Appl. Microbiol. Biotechnol. 2008; 80: 849–862. doi: 10.1007/s00253-008-1654-4.

5. Bahl H, Andersch W, Braun K, Gottschalk G. Effect of pH and butyrate concentration on the production of acetone and butanol by Clostridium acetobutylicum grown in continuous culture. European Journal of Applied Microbiology and Biotechnology. 1982; 14: 17–20. doi: 10.1007/BF00507998.

6. Beaty PS, McInerney MJ. Growth of Syntrophomonas wolfei in pure culture on crotonate. Arch. Microbiol. 1987; 147: 389–393. doi: 10.1007/BF00406138.

7. Beaty PS, McInerney MJ. Nutritional Features of Syntrophomonas wolfei. Appl. Environ. Microbiol. 1990; 56: 3223–3224.

8. Zhang A, Sun J, Wang Z, Yang S-T, Zhou H. Effects of carbon dioxide on cell growth and propionic acid production from glycerol and glucose by Propionibacterium acidipropionici. Bioresource Technol. 2015; 175: 374–381. doi: 10.1016/j.biortech.2014.10.046.

9. Barbirato F, Chedaille D, Bories A. Propionic acid fermentation from glycerol. Comparison with conventional substrates. Appl. Microbiol. Biotechnol. 1997; 47: 441–446. doi: 10.1007/s002530050953.

10. Seeliger S, Janssen PH, Schink B. Energetics and kinetics of lactate fermentation to acetate and propionate via methylmalonyl-CoA or acrylyl-CoA. FEMS Microbiology Letters. 2002; 211: 65–70. doi: 10.1111/j.1574-6968.2002.tb11204.x.

11. Beaty PS, McInerney MJ. Effects of Organic Acid Anions on the Growth and Metabolism of Syntrophomonas wolfei in Pure Culture and in Defined Consortia. Appl. Environ. Microbiol. 1989; 55: 977–983.

12. Buschhorn H, Dürre P, Gottschalk G. Production and Utilization of Ethanol by the Homoacetogen Acetobacterium woodii. Appl. Environ. Microbiol. 1989; 55: 1835–1840.

13. Peters V, Janssen PH, Conrad R. Efficiency of hydrogen utilization during unitrophic and mixotrophic growth of Acetobacterium woodii on hydrogen and lactate in the chemostat. FEMS Microbiology Ecology. 1998; 26: 317–324. doi: 10.1111/j.1574-6941.1998.tb00516.x.

14. Bainotti AE, Nishio N. Growth kinetics of Acetobacterium sp. on methanol‐formate in continuous culture. Journal of Applied Microbiology. 2000; 88: 191–201.

15. Godley A, Linnett P, Robinson J. The effect of carbon dioxide on the growth kinetics of fructose-limited chemostat cultures of Acetobacterium woodii DSM 1030. Arch. Microbiol. 1990; 154: 5–11. doi: 10.1007/BF00249170.
