# Supplementary material for: RedCom: A strategy for reduced metabolic modeling of complex microbial communities and its application for analyzing experimental datasets from anaerobic digestion
Source: PLoS Comput Biol. 2019 Feb 1;15(2):e1006759. doi: 10.1371/journal.pcbi.1006759 (PMC6373973; doi:10.1371/journal.pcbi.1006759)
Supplement: S4 Text — (DOCX) [file pcbi.1006759.s004.docx]

# S4 Text: Model validation

We summarize single-species biomass yields reported in the literature for growth of the organisms on different substrates and compare these data with model predictions (Table A). The observed biomass yields are typically dependent on the growth rate and the maintenance requirements. Measuring biomass yields for different growth rates in continuous processes enables not only estimation of the maintenance coefficient but also of the theoretical maximum biomass yield (biomass yield that could be achieved if there were no maintenance requirements) [1]. The theoretical maximum biomass yields and ATP yields were computed for all single-species models and compared with literature data (if available). In some cases no data were available especially for organisms that grow in syntrophy on certain substrates (e.g. *S. wolfei* growth on butyrate or *S. fumaroxidans* on propionate). The models of *D. vulgaris*, *M. hungatei*, *M. barkeri,* and *E. coli* have been published and validated before and are not discussed here again.

Table A: Overview of literature data and simulation results (single-species models) for (maximum) biomass yields and (maximum) ATP yields for the different organisms. In cases with more than one substrate at a time, the biomass yield refers to the underlined substrate.

| **Organism** | **Substrate** | **Reported biomass yield [gDW/mol]**  **(with reference)** | **Comment** | **Max. biomass yield model [gDW/mol]** | **Max. ATP yield model**  **[mol/mol]** |
| --- | --- | --- | --- | --- | --- |
| *P. freudenreichii* | glucose | 21.6-27 [2] | not max. biomass yield | 42.9 | 2.89 |
|  |  | 43.2 [3] |  |  |  |
|  | lactate | 9 [3] |  | 10.0 | 0.78 |
|  | Ethanol |  |  | 10.0 | 0.78 |
| *S. wolfei* | butyrate |  |  | 3.9 | 0.33 |
|  | crotonate | 14.6 [4] | not max. biomass yield | 12.9 | 0.83 |
| *A. woodii* | glucose |  |  | 62.4 | 4.45 |
|  | fructose | 61 [5] |  | 62.4 | 4.45 |
|  | lactate | 14.4 [6] |  | 13.4 | 1.08 |
|  | H_2_ + CO_2_ | 1.7 [6] |  | 1.4 | 0.11 |
|  | ethanol | 5.6 [7] | not max. biomass yield | 8.7 | 0.70 |
|  | ethanol + CO_2_ |  |  | 11.5 | 0.92 |
|  | methanol + CO_2_ | 8.1 [8] |  | 9.2 | 0.73 |
|  | methanol + formate | 16.5 [9] | (methanol : formate 1:1) | 10.6 | 0.85 |
| *S. fumaroxidans* | fumarate |  | (no accumulation of H_2_ or formate) | 12.3 | 0.7 |
|  | propionate |  | (syntrophic) | 6.6 | 0.5 |
|  | propionate + sulfate | 1.24 [10] | not max. biomass yield | 10.2 | 0.81 |

References

1. Pirt SJ. The maintenance energy of bacteria in growing cultures. Proceedings of the Royal Society of London. Series B, Biological sciences. 1965; 163: 224–231.

2. Zhang A, Sun J, Wang Z, Yang S-T, Zhou H. Effects of carbon dioxide on cell growth and propionic acid production from glycerol and glucose by Propionibacterium acidipropionici. Bioresource Technol. 2015; 175: 374–381. doi: 10.1016/j.biortech.2014.10.046.

3. Lewis V, Yang S-T. Propionic acid fermentation by Propionibacterium acidipropionici. Effect of growth substrate. Appl. Microbiol. Biotechnol. 1992; 37. doi: 10.1007/BF00180964.

4. Beaty PS, McInerney MJ. Growth of Syntrophomonas wolfei in pure culture on crotonate. Arch. Microbiol. 1987; 147: 389–393. doi: 10.1007/BF00406138.

5. Godley A, Linnett P, Robinson J. The effect of carbon dioxide on the growth kinetics of fructose-limited chemostat cultures of Acetobacterium woodii DSM 1030. Arch. Microbiol. 1990; 154: 5–11. doi: 10.1007/BF00249170.

6. Peters V, Janssen PH, Conrad R. Efficiency of hydrogen utilization during unitrophic and mixotrophic growth of Acetobacterium woodii on hydrogen and lactate in the chemostat. FEMS Microbiology Ecology. 1998; 26: 317–324. doi: 10.1111/j.1574-6941.1998.tb00516.x.

7. Buschhorn H, Dürre P, Gottschalk G. Production and Utilization of Ethanol by the Homoacetogen Acetobacterium woodii. Appl. Environ. Microbiol. 1989; 55: 1835–1840.

8. Bainotti AE, Yamaguchi K, Nakashimada Y, Nishio N. Kinetics and energetics of Acetobacterium sp. in chemostat culture on methanol-CO 2. Journal of fermentation and bioengineering. 1998; 85: 223–229.

9. Bainotti AE, Nishio N. Growth kinetics of Acetobacterium sp. on methanol‐formate in continuous culture. Journal of Applied Microbiology. 2000; 88: 191–201.

10. Scholten JCM, Conrad R. Energetics of Syntrophic Propionate Oxidation in Defined Batch and Chemostat Cocultures. Appl. Environ. Microbiol. 2000; 66: 2934–2942. doi: 10.1128/AEM.66.7.2934-2942.2000.
