# Supplementary material for: RedCom: A strategy for reduced metabolic modeling of complex microbial communities and its application for analyzing experimental datasets from anaerobic digestion
Source: PLoS Comput Biol. 2019 Feb 1;15(2):e1006759. doi: 10.1371/journal.pcbi.1006759 (PMC6373973; doi:10.1371/journal.pcbi.1006759)
Supplement: S5 Text — (DOCX) [file pcbi.1006759.s005.docx]

# S5 Text: Simulation results of the three-species community model

We carried out simulations with the bilinear, the linearized full and the (linear) reduced three-species community model (organisms: *D. vulgaris*, *M. hungatei*, *M. barkeri*; see also main text) by determining for each model the feasible ranges of fractional abundances and exchange rates. For all three models we considered two scenarios (Table A): in the first scenario we allowed accumulation of acetate and enabled external CO_2_ supply, whereas in the second scenario, accumulation of acetate and uptake of external CO_2_ was not allowed. The reason for us to look at the acetate accumulation scenario was a study of Tatton et al. (2009) [1] were a two-species community of a *Desulfovibrio* and *Methanobacterium* was investigated. Due to pH-control acetate accumulation was enabled and we thus wanted to include that case.

In case of the linearized full and reduced model we considered in addition four different community growth rates corresponding to 99%, 50%, 5% and 0% of $\mu_{c,max}$ and computed then for each of these cases feasible ranges of fractional abundances and exchange rates (via flux variability analysis with linear optimizations) as well as for methane yield (via linear-fractional optimization [2])

Generally, the broadest ranges for exchange rates, community composition, and methane yield can be found for the bilinear model (Table A). This is not surprising given that these predictions are made for all possible growth rates (dilution rates). All models predict correctly that *D. vulgaris* is essential for the community while the methanogens are compositionally variable in the first scenario and *M. barkeri* essential in the second scenario where no acetate accumulation is allowed.

Furthermore, for the linear full and reduced model, the largest ranges in feasible biomass compositions and exchange fluxes can be seen for small $\mu_{c}$ while with increasing $\mu_{c}$ this flexibility decreases. Generally, narrower ranges for biomass composition, exchange rates, and methane yield can be observed for the second scenario (no acetate accumulation). Most importantly, due to the exclusion of unrealistic solutions with low biomass yields in the single-species models, the reduced model has a smaller solution space compared to the linearized full model resulting in significantly smaller predicted ranges (especially for exchange rates). This is particularly pronounced in the second scenario (no acetate accumulation) and, in both scenarios, under low growth rates.

Table A: Predicted ranges of the community composition (F_x_: fractional biomass abundance of species x, DV: *D. vulgaris*, MH: *M. hungatei*, MB: *M. barkeri*), methane yield (Y_CH4/Eth_), substrate (ethanol) uptake, and product formation rates (mmol/gDW_c_/h) (r_Eth_: ethanol uptake, r_CH4_: methane excretion, r_Ac_: acetate excretion) in the bilinear, the linearized full, and the reduced three-species model. For the linearized and reduced model, $\boldsymbol{\mu}_{\boldsymbol{c}}$ was fixed to 99%, 50%, 5%, and 0% of the $\boldsymbol{\mu}_{\boldsymbol{c,max}}$ of the community model. Two scenarios were simulated: without acetate accumulation (complete degradation of produced acetate) and with acetate accumulation and additional CO_2_ supply.

|  | $\boldsymbol{\mu}_{\boldsymbol{c}}$ [h^-1^] | **F_DV_** | **F_MH_** | **F_MB_** | **Y_CH4/Eth_** | **r_Eth_** | **r_CH4_** | **r_Ac_** |
| --- | --- | --- | --- | --- | --- | --- | --- | --- |
| **Accumulation of acetate enabled** | | | | | | | | |
| bilinear | 0-0.052 (predicted) | 0.024-0.54 | 0-0.93 | 0-0.98 | 0.39-1.50 | 1.19-19.99 | 1.48-12.5 | 0-19.68 |
| linearized  full model | fixed to 0.052 (99% $\mu_{c,max}$) | 0.074-0.39 | 0-0.68 | 0-0.93 | 0.39-1.37 | 3.76-19.98 | 3.58-12.34 | 0-19.67 |
| reduced model |  | 0.074-0.32 | 0-0.68 | 0-0.93 | 0.39-1.24 | 3.76-16.03 | 3.58-7.14 | 0-15.79 |
| linearized  full model | fixed to  0.026 (50% $\mu_{c,max}$) | 0.050-0.45 | 0-0.79 | 0-0.95 | 0.41-1.43 | 2.49-19.37 | 2.54-12.42 | 0-19.22 |
| reduced model |  | 0.065-0.26 | 0-0.74 | 0-0.94 | 0.41-1.30 | 2.49-10.04 | 2.54-4.55 | 0-9.93 |
| linearized  full mode | fixed to 0.0026 (5% $\mu_{c,max}$) | 0.027-0.53 | 0-0.92 | 0-0.97 | 0.48-1.49 | 1.32-18.81 | 1.58-12.49 | 0-18.80 |
| reduced model |  | 0.049-0.15 | 0-0.86 | 0-0.95 | 0.48-1.46 | 1.32-3.93 | 1.58-2.07 | 0-3.92 |
| linearized  full model | fixed to 0 (0% $\mu_{c,max}$) | 0.024-0.54 | 0-0.93 | 0-0.98 | 0.5-1.5 | 1.19-18.75 | 1.48-12.5 | 0-18.75 |
| reduced model |  | 0.046-0.12 | 0-0.88 | 0-0.95 | 0.5-1.5 | 1.19-3.16 | 1.48-1.84 | 0-3.16 |
| **Without accumulation of acetate** | | | | | | | | |
| bilinear | 0-0.0520 (predicted) | 0.024-0.28 | 0-0.76 | 0.17-0.98 | 1.17-1.5 | 1.19-9.04 | 1.79-12.5 | 0 |
| linearized  full model | fixed to 0.052 (99% $\mu_{c,max}$) | 0.074-0.18 | 0-0.35 | 0.49-0.93 | 1.17-1.37 | 3.76-9.04 | 4.42-12.34 | 0 |
| reduced model |  | 0.074-0.092 | 0-0.20 | 0.71-0.93 | 1.17-1.24 | 3.76-4.63 | 4.41-5.74 | 0 |
| linearized  full model | fixed to  0.026 (50% $\mu_{c,max}$) | 0.050-0.22 | 0-0.47 | 0.39-0.95 | 1.25-1.43 | 2.49-8.69 | 3.12-12.42 | 0 |
| reduced model |  | 0.065-0.078 | 0-0.22 | 0.70-0.94 | 1.25-1.30 | 2.49-2.99 | 3.12-3.89 | 0 |
| linearized  full model | fixed to 0.0026 (5% $\mu_{c,max}$) | 0.027-0.27 | 0-0.72 | 0.21-0.97 | 1.45-1.49 | 1.32-8.37 | 1.92-12.49 | 0 |
| reduced model |  | 0.049-0.053 | 0-0.31 | 0.64-0.95 | 1.45-1.46 | 1.32-1.42 | 1.92-2.07 | 0 |
| linearized  full model | fixed to 0 (0% $\mu_{c,max}$) | 0.024-0.28 | 0-0.76 | 0.17-0.98 | 1.5 | 1.19-8.33 | 1.79-12.5 | 0 |
| reduced model |  | 0.046-0.048 | 0-0.34 | 0.61-0.95 | 1.5 | 1.19-1.22 | 1.79-1.84 | 0 |

References

1. Tatton MJ, Archer DB, Powell GE, Parker ML. Methanogenesis from ethanol by defined mixed continuous cultures. Appl. Environ. Microbiol. 1989; 55: 440–445.

2. Klamt S, Müller S, Regensburger G, Zanghellini J. A mathematical framework for yield (vs. rate) optimization in constraint-based modeling and applications in metabolic engineering. Metab. Eng. 2018; 47: 153–169. doi: 10.1016/j.ymben.2018.02.001.
