# Supplementary material for: RedCom: A strategy for reduced metabolic modeling of complex microbial communities and its application for analyzing experimental datasets from anaerobic digestion
Source: PLoS Comput Biol. 2019 Feb 1;15(2):e1006759. doi: 10.1371/journal.pcbi.1006759 (PMC6373973; doi:10.1371/journal.pcbi.1006759)
Supplement: S6 Text — (DOCX) [file pcbi.1006759.s006.docx]

# S6 Text: Experimental setup and analysis of an ethanol enrichment culture

## Cultivation

For the cultivation on ethanol performed in this study, we used biomass from an enrichment that was run 520 days on glucose-cellulose medium [1]. As main carbon sources, glucose and cellulose were replaced by 14.6% (v/v) ethanol; all other medium components were left the same. Cultivations were carried out in a Sixfors multi bioreactor system (INFORS AG, Bottmingen, Switzerland) with three glass vessels of 400 mL working volume at 40°C and semi-continuous feeding to allow adaption of microbial communities to the new substrate.

After another 80 days, 150 mL of the reactor content of these small-scale cultures were pooled and transferred to one of two 1.5 L BioFlo 320 bioreactor systems (Unit 1: U1, Unit 2, U2; Eppendorf AG, Hamburg, Germany) each. The vessels were equipped as follows: online pH electrode (ISM 12, Mettler Toledo, Columbus, USA), probe for temperature control, sampling port, exhaust cooler, stirrer with direct drive motor (Industrial Indexing Systems; Victor, USA), feeding (1.59 mm inner diameter tubing) and waste (4.76 mm inner diameter tubing) ports coupled to onboard pumps and a scale for volume control. Gastight tubing (AdvantaPure, NewAge Industries; Southampton, USA) were used to connect the exhaust cooler to the anaerobic lab fermentation system GärOnA with integrated gas analysis (Gesellschaft zur Förderung von Medizin- Bio- und Umwelttechnologie e.V., Halle, Germany), which was coupled to an online gas chromatography system (GC, ECH Elektrochemie Halle GmbH, Halle Germany, see Kohrs et al. [1] for equipment and setup details). Both the reactors and the feed bottles were covered with aluminum foil and the latter stirred constantly to avoid possible inhomogeneity due to precipitations.

After inoculation, medium containing water instead of ethanol was used to adjust reactor volumes to 1 L; process parameters were set to 40°C temperature and 100 rpm agitation. By applying a feeding rate of 7 mL every 8 h, a fed batch mode was implemented for the following days to adjust reactor volumes stepwise to 1.5 L. After reaching 1.5 L, the continuous cultivation mode was initiated using a constant feeding rate and automatic volume control. In the following, different hydraulic retention times were targeted to reach steady-state conditions, starting at hydraulic retention times (HRT) of approximately 100 d followed by incremental reduction of 10 d every 1-2 weeks. Steady states were reached and experimental data are available for HRTs between 79 and 33 days corresponding to dilution rates of 5.3⋅10^-4^ h^-1^ to 1.7⋅10^-3^ h^-1^. The so-called transient state cultivation [2] allows recording multiple virtual steady-states in shorter time periods compared to the regular continuous process mode. Due to mechanical fatigue of tubing, the transport rates of the peristaltic pumps decreased over time. Therefore, true feeding rates we estimated by regular weighting of the feed bottles (what explains uneven HRT values). Steady-state conditions were considered being reached when the following process parameters remained stable for at least one day: pH value, biogas production rate and biogas composition [3].

After reaching steady-state conditions, samplings for subsequent analyses were performed: samples taken for the determination of ethanol and organic acids were first centrifuged for 10 min at 16,400 x g, supernatants were then filtered (0.2 µm Whatman SPARTANTM 13/0.2 RC Filter Units, GE Healthcare Life Sciences, München, Deutschland) using disposable syringes and stored at -20°C. Samples for metaproteomics analyses and Lowry assay were directly stored at -20°C until further use.

## Enzymatic determination of ethanol

Ethanol concentrations were determined indirectly by enzymatic conversion to acetaldehyde and further to acetic acid + NADH. The latter was detected in a UV photometer at 340 nm wavelength following the manufacturer’s instructions (kit# 10176290035; Boehringer Mannheim/R-Biophram AG, Darmstadt, Germany).

## Chromatographic determination of organic acids.

Lactate, acetate, propionate, formate and butyrate were quantified by anion exchange chromatography (Dionex ICS-5000 Reagent-Free HPLC System, Thermos Fisher Scientific Inc., Waltham, USA). Separation of acids was performed on two anion exchange columns (Dionex IonPac AS11 Analytical Column 2 x 250 mm, Thermo Fisher Scientific Inc.) in a potassium hydroxide gradient followed by conductivity detection (see [1] for details).

## Determination of protein content by Lowry assay.

Protein quantification by Lowry assay [4] was carried out in triplicates with a preceding cell lysis step as follows. For homogenization of flocks of microorganisms, 200 µL sample material was given to 1 g silica beads (diameter of 0.5 mm, BioSpec Products Inc., Bartlesville, USA) and shaken for 1 min at 30 Hz in a ball mill (MM 400, Retsch GmbH, Haan, Germany). Subsequently, 25 µL of suspension was mixed with 100 µL of 0.2 M NaOH for 1 min at 100°C. After cooling to ambient temperature, 1 mL of a solution containing 30 g L^-1^ Na_2_CO_3_, 0.2 g L^-1^ Cu_2_SO_4_ dihydrate, and 0.4 g L^-1^ KNaC_4_H_4_O_6_ tetrahydrate was added and incubated for 10 min before adding another 50 µL of Folin's phenol reagent followed by incubation in the dark for 45 min. Finally, optical density was measured at 700 nm wavelength in a UV photometer and protein concentrations determined by reference to a standard curve of bovine serum albumin.

References

1. Kohrs F, Heyer R, Bissinger T, Kottler R, Schallert K, Püttker S, et al. Proteotyping of laboratory-scale biogas plants reveals multiple steady-states in community composition. Anaerobe. 2017. doi: 10.1016/j.anaerobe.2017.02.005.

2. Müller RH, Bley T, Babel W. Transient state cultivation as a means for determining maximum growth rates of microorganism in inhibition kinetics. J. Microbiol. Methods. 1995; 22: 209–219. doi: 10.1016/0167-7012(94)00075-I.

3. Luo G, Xie L, Zhou Q, Angelidaki I. Enhancement of bioenergy production from organic wastes by two-stage anaerobic hydrogen and methane production process. Bioresource Technol. 2011; 102: 8700–8706. doi: 10.1016/j.biortech.2011.02.012.

4. Lowry OH, Rosebrough NJ, Farr AL, & Randall RJ. Protein Measurement with the folin phenol reagent. J. Biol. Chem. 1951: 265–275.
