# Supplementary material for: RedCom: A strategy for reduced metabolic modeling of complex microbial communities and its application for analyzing experimental datasets from anaerobic digestion
Source: PLoS Comput Biol. 2019 Feb 1;15(2):e1006759. doi: 10.1371/journal.pcbi.1006759 (PMC6373973; doi:10.1371/journal.pcbi.1006759)
Supplement: S7 Text — (DOCX) [file pcbi.1006759.s007.docx]

# S7 Text: Simulation results for the six-species community model and experimental data from the ethanol enrichment culture

Simulations were carried out with a six- species community model (organisms: *A. woodii*, *P. freudenreichii*, *D. vulgaris*, *S. fumaroxidans*, *M. barkeri*, *M. hungatei*) for different growth rates with the linearized full and reduced model. The simulation results were compared with experimental data (Table A) from cultivations in two bioreactors. The growth rates were fixed to the respective dilution rates of the experiments.

The experimental data lie always in the predicted ranges for the exchange rates and close to the predicted ranges for methane yield and methane content (deviation almost always below 10%). However, the predicted ranges were (partially significantly) smaller for the reduced model compared to the full model while still covering the experimental data equally well. Hence, the reduced model has a significantly higher predictive power.

Table A: Simulation results of the linearized full model and of the reduced model for the six-species community and experimental data from the enrichment culture for growth on ethanol. In the simulations, $\boldsymbol{\mu}_{\boldsymbol{c}}$ was fixed to the respective dilution rates of the experiments and accumulation of organic acids was switched off (according to experimental data). The last row shows the feasible ranges of rates and yields as predicted by the bilinear model (growth rate unconstrained; accumulation of organic acids switched off according to experimental data). The predicted ranges are colored according to their deviation from the experimental data: dark green: data are in the predicted range, light green: deviation below 5%, yellow: deviation between 5% and 10%, orange: deviation between 10% and 15%. The six species in the model are: *P. freudenreichii*, *A. wooddii*, *D. vulgaris*, *S. fumaroxidans*, *M. barkeri* and *M. hungatei.*

|  | Dilution rate  [h^-1^] | Exchange rates [mmol/gDW_c_/h] | | | | | | | | | Product yields/ratios  [mol/mol] | | | | | |
| --- | --- | --- | --- | --- | --- | --- | --- | --- | --- | --- | --- | --- | --- | --- | --- | --- |
|  |  | Ethanol | | | CO_2_ | | | CH_4_ | | | CH_4_:CO_2_ | | | CH_4_:Ethanol | | |
|  |  | Full model | Red. model | Exper. | Full model | Red. model | Exper. | Full model | Red. model | Exper. | Full model | Red. model | Exper. | Full model | Red. model | Exper. |
| Reactor 1 | 0.00054 | 0.19-8.34 | 0.19-1.27 | 0.43 | 0.083-4.16 | 0.084-0.62 | 0.17 | 0.27-12.50 | 0.27-1.89 | 0.59 | 3.01-3.24 | 3.03-3.24 | 3.56 | 1.44-1.50 | 1.44-1.49 | 1.38 |
|  | 0.00060 | 0.19-8.34 | 0.19-1.27 | 0.49 | 0.084-4.16 | 0.084-0.62 | 0.19 | 0.27-12.50 | 0.27-1.89 | 0.67 | 3.01-3.26 | 3.04-3.27 | 3.50 | 1.43-1.50 | 1.43-1.49 | 1.39 |
|  | 0.00072 | 0.20-8.34 | 0.20-1.28 | 0.58 | 0.084-4.16 | 0.085-0.63 | 0.22 | 0.28-12.50 | 0.28-1.90 | 0.75 | 3.01-3.31 | 3.05-3.31 | 3.47 | 1.42-1.50 | 1.42-1.49 | 1.29 |
|  | 0.00085 | 0.20-8.34 | 0.20-1.29 | 0.67 | 0.085-4.15 | 0.085-0.63 | 0.30 | 0.29-12.50 | 0.289-1.91 | 0.99 | 3.01-3.37 | 3.05-3.37 | 3.31 | 1.41-1.50 | 1.41-1.48 | 1.47 |
|  | 0.0010 | 0.21-8.35 | 0.21-1.30 | 0.42 | 0.086-4.15 | 0.086-0.63 | 0.19 | 0.29-12.50 | 0.29-1.93 | 0.61 | 3.01-3.43 | 3.06-3.43 | 3.25 | 1.40-1.50 | 1.40-1.48 | 1.47 |
|  | 0.0012 | 0.22-8.35 | 0.22-1.32 | 0.56 | 0.087-4.15 | 0.087-0.63 | 0.25 | 0.30-12.50 | 0.30-1.95 | 0.86 | 3.01-3.49 | 3.07-3.49 | 3.44 | 1.38-1.50 | 1.38-1.48 | 1.52 |
|  | 0.0017 | 0.26-8.36 | 0.26-1.36 | 0.66 | 0.089-4.14 | 0.089-0.64 | 0.29 | 0.33-12.49 | 0.33-1.89 | 0.93 | 3.02-3.72 | 3.11-3.72 | 3.17 | 1.34-1.50 | 1.34-1.47 | 1.42 |
| Reactor 2 | 0.00053 | 0.19-8.34 | 0.189-1.27 | 0.57 | 0.083-4.16 | 0.083-0.62 | 0.22 | 0.27-12.50 | 0.27-1.89 | 0.76 | 3.00-3.23 | 3.03-3.24 | 3.52 | 1.44-1.50 | 1.44-1.49 | 1.34 |
|  | 0.00058 | 0.19-8.34 | 0.19-1.28 | 0.50 | 0.084-4.16 | 0.084-0.62 | 0.19 | 0.27-12.50 | 0.27-1.90 | 0.65 | 3.00-3.26 | 3.04-3.26 | 3.46 | 1.43-1.50 | 1.43-1.49 | 1.32 |
|  | 0.00071 | 0.20-8.34 | 0.20-1.29 | 0.67 | 0.084-4.16 | 0.084-0.63 | 0.26 | 0.28-12.50 | 0.28-1.92 | 0.88 | 3.01-3.31 | 3.04-3.31 | 3.37 | 1.42-1.50 | 1.42-1.49 | 1.32 |
|  | 0.00083 | 0.20-8.34 | 0.20-1.30 | 0.78 | 0.085-4.16 | 0.085-0.63 | 0.34 | 0.29-12.50 | 0.29-1.93 | 1.12 | 3.01-3.36 | 3.05-3.36 | 3.27 | 1.41-1.50 | 1.41-1.49 | 1.43 |
|  | 0.0010 | 0.21-8.35 | 0.21-1.31 | 0.75 | 0.086-4.15 | 0.086-0.63 | 0.34 | 0.29-12.50 | 0.30-1.94 | 1.12 | 3.01-3.43 | 3.06-3.43 | 3.25 | 1.40-1.50 | 1.40-1.48 | 1.48 |
|  | 0.0012 | 0.22-8.35 | 0.22-1.36 | 0.64 | 0.087-4.15 | 0.087-0.63 | 0.27 | 0.31-12.50 | 0.31-1.99 | 0.93 | 3.01-3.53 | 3.08-3.53 | 3.46 | 1.38-1.50 | 1.38-1.48 | 1.45 |
| Feasible ranges in bilinear model | | 0.16-9.03 | | | 0.080-4.17 | | | 0.24-12.50 | | | 3-14.47 | | | 0.97-1.5 | | |
