# Supplementary material for: RedCom: A strategy for reduced metabolic modeling of complex microbial communities and its application for analyzing experimental datasets from anaerobic digestion
Source: PLoS Comput Biol. 2019 Feb 1;15(2):e1006759. doi: 10.1371/journal.pcbi.1006759 (PMC6373973; doi:10.1371/journal.pcbi.1006759)
Supplement: S8 Text — (DOCX) [file pcbi.1006759.s008.docx]

# S8 Text: Metaproteomic analysis of ethanol enrichment culture and resulting model adjustments

## Metaproteomic measurements

Samples of the ethanol enrichment culture (see S6 Text) were treated as described by Heyer et al. [1] with minor changes. Briefly, 2 mL of sample sludge were mixed with 5 g silica beads, 2 mL of 2 M sucrose solution and 3.5 mL of phenol solution (10 g mL^-1^) in a 50 mL vessel. Lysis was achieved by shaking in a FastPrep-96 homogenizer (MP Biomedicals, Santa Ana, USA) for 5 min at 30 Hz. After centrifugation for 10 min at 10,000 x g, supernatant phenol phase was transferred to a new vial and mixed with (v/v) 1 M sucrose solution for 10 min, followed by another centrifugation and transfer of the phenol phase. Proteins where then precipitated by ammonium acetate in methanol, washed with 80% acetone and 70% ethanol before being solved in urea/thiourea buffer. Protein quantification was carried out using amido black dye with 25 µg of protein precipitated and loaded for SDS-PAGE. After staining with colloidal Coomassie G-250, sample lanes were chopped off the gel for tryptic in gel digestion. Extracted peptides were analyzed by LC-MS/MS (UltiMate 3000 nano splitted LC system and Orbitrap Elite™ Hybrid Ion Trap-Orbitrap MS system, Thermo Fisher Scientific Inc., Walthem, USA). For details of the LC and MS configurations as well as the acquisition parameters, we refer to Kohrs et al. [2]. Recorded spectra were converted into .mgf-file format for subsequent import to the MetaProteomeAnalyzer [3] software (version 2.11, <http://www.mpa.ovgu.de/>), in which X!Tandem [4] and OMSSA [5] performed protein identifications (default settings) against UniProtKB/Swiss-Prot and seven metagenome sequence sets, followed by BLAST [6] to derive protein annotations (for respective details see Kohrs et al. [2]). Metaproteins were assigned using the peptide rule ‘share at least one peptide’ with FDR ≤5% before being exported and evaluated on the basis of normalized SpC.

## Analysis of the metaproteomic data

We analyzed the metaproteome of the enrichment culture aiming for a taxonomic characterization. Furthermore, identification of certain marker proteins can help to distinguish between metabolic routes used or not used by the community. As described before, we chose organisms representing certain functional guilds to model anaerobic digestion. Typically, hundreds of organisms are involved in anaerobic digestion in a real biogas plant. Thus, the question arises whether we can map the organisms found in our enrichment culture to the organisms of our model and whether there are further groups of organisms involved that we have not considered so far.

In a first step, we looked at the spectral counts for the different superkingdoms (see Figure A) and considered them to correlate with the abundance of those groups of organisms. We found between 40 and 50% archaea, 20-25% bacteria, 10-15% eukaryota and small amounts (<1%) of viruses. Additionally, a little more than 20% of the spectra could not be assigned to any superkingdom (referred to as unknown). All methanogens are archaea and all ethanol oxidizers belong to the bacteria superkingdom. Viruses and eukaryota are not considered in the model. Eukaryotic cells may come from impurities (keratin) or plant residues from sludge forming persistent flocks and are not considered in the following.

In a second step, to distinguish between the different functional guilds, we examined the spectral abundance on the taxonomic level of orders. The percentage of not assigned spectra was on average 50% of the spectra. The most abundant taxonomic orders among the assigned spectra (spectral abun­dance >5% in at least one sample, see Figure 7 in the main text) were Methanosarcinales, Methano­­bacteriales, Desulfovibrionales, Enterobacteriales, Methanomicrobiales, Methano­coccales, Bacillales, Clostridiales and Archaeoglobales. Most of these groups can be mapped to organisms in the model. *D. vulgaris* represents the order Desulfovibrionales, *M. barkeri* represents the order Methano­sarcinales and *M. hungatei* the orders Methano­bacteriales, Methano­microbiales and Methano­coccales since the organisms in these orders all use either formate or hydrogen plus CO_2_ for methano­gensis. Baccillales accounting on average for 5.4% of the assigned spectra on order level are not expected to play a role for anaerobic digestion because they contain mainly aerobic organisms [7,8]. However, they have also been repeatedly identified in biogas plants [9] and a few species seem to be able to grow anaerobi­cally as well [10,11]. Archaeoglobales accounting for 3.8% on average are also not expected because this order contains hyperthermophilic archaea and cul­ti­vations were per­formed under mesophilic conditions. The proteins found in those organisms were house­­keeping proteins and thus do not allow conclusions on metabolic functions related to the process.

While we did not expect Enterobacteriales to be involved in ethanol oxidation, 79% of the proteins assigned to Enterobacteriales were hits for an alcohol dehydrogenase (EC 1.1.1.1). This enzyme is present in many organisms of Enterobacteriales, including *E. coli* but usually ethanol is produced and not consumed by these organisms. Additionally, we found large amounts of aldehyde dehydrogenase (EC 1.2.99.7), which is part of the same pathway (ethanol production or ethanol oxidation), assigned to Desulfovibrionales. We expect both enzymes to be originating from the same organism and since we did not find an entry in the Swiss-Prot database [12] for alcohol dehydrogenase (EC 1.1.1.1) in the Desulfovibrionales, we assume that it was wrongly assigned to Enterobacteriales by BLAST but originates from Desulfovibrionales. Thus, we manually assigned the spectral counts for alcohol dehydrogenase to Desulfovibrionales. Additionally, Clostridiales were among the most abundant orders. However, Clostridiales is a diverse group of organisms and the enzymes found were not specific for a certain pathway (among most abundant proteins: malate dehydrogenase, groEL, cell wall proteins).


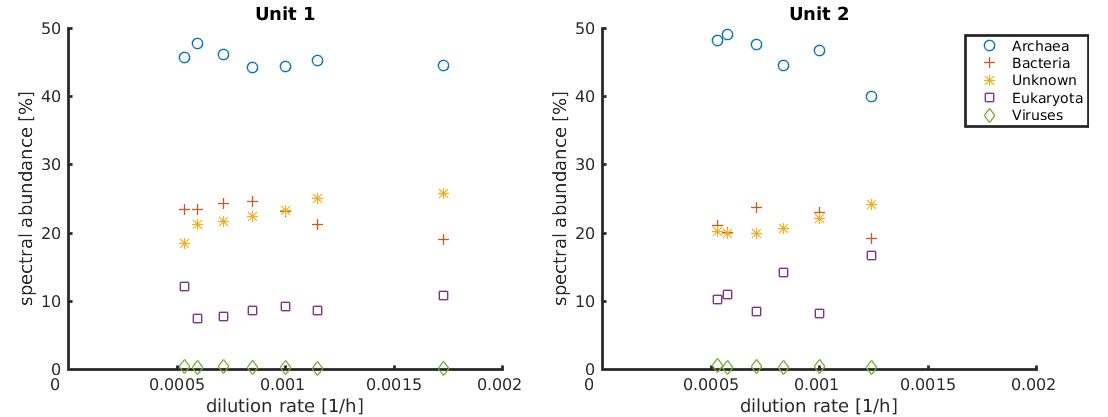


Figure A: Normalized spectral counts for the community composition at the superkingdom level for the ethanol enrichment culture with different dilution rates.

## Enzyme analysis

Some general metabolic pathways are spread among several different taxa whereas others can be specific for only very few. That makes mapping of taxa to functional guilds in the model difficult and additional analysis becomes necessary. We therefore looked for enzymes, which are specific for certain pathways. Pathways of interest in our process were ethanol oxidation, acetate production or consumption (homoacetogenesis, syntrophic acetate oxidation or acetoclastic methanogenesis), hydrogenotrophic methanogenesis and the methylmalonyl-CoA pathway. Many enzymes are involved in different pathways (e.g. acetate production and consumption, methanogenesis from acetate and methanogensis from hydrogen). In these cases, identifying the taxonomic group can help to distinguish between the pathways. For example, carbon monoxide dehydrogenase and CO-methylating acetyl-CoA synthase originating from archaea indicate for acetoclastic methanogenesis, whereas bacterial origin of these enzymes hint for homoacetogenesis or syntrophic actetate oxidation [13]. Table A shows a list of potential key enzymes for different pathways that might be relevant in our process. We chose enzymes that are specific for only one pathway. Among the ten most abundant enzymes we found enzymes for methanogenesis (5,10-methylenetetrahydromethanopterin reductase (EC:[1.5.98.2](http://www.genome.jp/dbget-bin/www_bget?ec:1.5.98.2), SpC: 785), methyl-coenzyme M reductase (EC:[2.8.4.1](http://www.genome.jp/dbget-bin/www_bget?ec:2.8.4.1), SpC: 1,585), methylene­tetra­hydromethanopterin dehydrogenase (EC:[1.5.98.1](http://www.genome.jp/dbget-bin/www_bget?ec:1.5.98.1), SpC: 700), tetrahydromethanopterin S-methyl­transferase (EC:[2.1.1.86](http://www.genome.jp/dbget-bin/www_bget?ec:2.1.1.86), SpC: 937)), acetate production and acetoclastic methano­genesis (acetyl-CoA synthetase (EC:[6.2.1.1](http://www.genome.jp/dbget-bin/www_bget?ec:6.2.1.1), SpC: 688), acetyl-CoA decarbonylase/synthase complex (EC:[1.2.7.4](http://www.genome.jp/dbget-bin/www_bget?ec:1.2.7.4), SpC: 588)) and ethanol oxidation (alcohol dehydrogenase (EC: 1.1.1.1, SpC: 594)). Other highly abundant proteins were not specific for any major degradation pathway. A few hits were found for propionyl-CoA carboxylase (EC 6.4.1.3) and methyl­malonyl-CoA carboxytransferase (EC 2.1.3.1), which can be involved in propionate fermen­tation. Compared to others pathways, the abundance of these enzymes was low (average: 8.23) indicating only a minor relevance for the process. We also found only little evidence for homo­acetogenesis. There were only a few hits (on average 2.35 spectra) for 2.3.1.169 assigned to *Moorella thermoacetica*, which is a homoacetogenic organism capable of using several substrates including ethanol and hydrogen plus carbon dioxide.

Table A: Potential key enzymes for different pathways and the expected taxonomic superkingdom as well as the average number of spectral Count (SpeC) found in the ethanol enrichment culture according to LC-MS/MS analysis.

| **Pathway** | **Enzyme** | **Expected taxonomy** | **Average SpeC** |
| --- | --- | --- | --- |
| Ethanol oxidation | Alcohol dehydrogenase (EC 1.1.1.1) | Bacteria | 584.6 |
|  | Aldehyde dehydrogenase (EC 1.2.99.7) | Bacteria | 763.2 |
| Methanogenesis from acetate | 5-methyltetrahydrosarcinapterin:corrinoid/iron-sulfur protein Co-methyltransferase (2.1.1.245) | Archaea | 0 |
|  | anaerobic carbon-monoxide dehydrogenase  (EC 1.2.7.4) | Archaea | 569.4 |
|  | CO-methylating acetyl-CoA synthase (EC 2.3.1.169) | Archaea | 38.9 |
|  | acetyl-CoA synthetase (EC 6.2.1.1) | Archaea | 508 |
| Methanogenesis from CO_2_ + H_2_ or formate | methylenetetrahydromethanopterin dehydrogenase (EC 1.5.98.1) | Archaea | 665.1 |
|  | 5,10-methylenetetrahydromethanopterin reductase (EC 1.5.98.2) | Archaea | 723.8 |
| Homoacetogenesis (from CO_2_ + H_2_) | anaerobic carbon-monoxide dehydrogenase (EC 1.2.7.4) | Bacteria | 0.00 |
|  | CO-methylating acetyl-CoA synthase (EC 2.3.1.169) | Bacteria | 2.35 |
| Propionate degradation | Propionate CoA-transferase (EC 2.8.3.1) | Bacteria | 0.00 |
|  | Propionyl-CoA carboxylase (EC 6.4.1.3) | Bacteria | 8.23 |
| Methylmalonyl-CoA pathway | methylmalonyl-CoA carboxytransferase (EC 2.1.3.1) | Bacteria | 8.23 |
|  | (S)-methylmalonyl-CoA decarboxylase (EC 4.1.1.41) | Bacteria | 0.00 |

## Refinement of the six-species model

Overall, the metaproteomic analyses presented above indicate that homoacetogenesis (*A. woodii*) and ethanol oxidation via propionate (*P. freudenreichii* and *S. fumaroxidans*) seem to be of minor relevance. As a consequence, mainly three out of the six (guild) organisms of the original six-species model are relevant to represent the ethanol enrichment culture. These organisms are *D. vulgaris*, *M. hungatei* and *M. barkeri*, which, accidentally, exactly correspond to the three-species community studied in the first subsection of the Results section in the main text. Accordingly, we adapted the reduced six-species model to reflect this composition (basically, the fractional biomass abundance of A. woodii, *P. freudenreichii* and *S. fumaroxidans* was set to zero). Further analysis also revealed that the majority of the spectral counts in Methanosarcinales belonged to the Methanosaeta species, which can only use acetate for methanogenesis. Since we have Methanosarcina instead of Methanosaeta in our model, we also switched off the hydrogen uptake for *M. barkeri* to mimic Methanosaeta.

Including the respective constraints for the observations made in the six-species model (which then becomes effectively a three-species model) we can further confine the solution space of the reduced model (Table B; selected results for $\mu_{c}=$ 0.001 h^-1^ are given in Table 4 in the main text). Due to the tighter constraints, the rates of specific ethanol uptake as well as of CO_2_ and methane production can now be determined exactly (no range) but the predictions appear, again, to be higher compared to the experimental data (Sim1 in Table B). With the results from the six-species model, we may assume that the comparably very high maintenance coefficient of *D. vulgaris* (4.3 mmolATP/gDW/h) was overestimated. Indeed, using instead a common maintenance coefficient of 1 mmolATP/gDW/h for all three remai­ning species (Sim2 in Table B) the simulation results are closer to experimental data (Table B) thus confirming a likely overestimation of the *D. vulgaris* maintenance coefficient. For Sim2, the simulation results deviated less than 0.5 mmol/gDW/h and methane yields and methane to CO_2_ ratio deviated less than 15% from the experimental data. Given a relatively high variation of the rate and yield measurements (cf. data for $\mu_{c}=$ 0.001 h^-1^ in Table B) a reasonable agreement between experimental data and predictions can be concluded.

Table B: Simulation results and experimental data for specific substrate uptake and product formation rates as well as methane yield and biogas composition. For simulations we used constraints according to mass-spectrometric data (disabling three of the six organisms and certain pathways inn the reduced six-species model; see description above). We performed two simulations, the first (Sim1) with the original maintenance coefficients from the single-species models and the second (Sim2) with a unique maintenance coefficient of 1 mmolATP/gDW/h for all involved organisms. The simulation results are colored according to the deviation to the experimental data: for the exchange rates: dark green: deviation <0.1 mmol/gDW/h, light green: between 0.1 and 0.5 mmol/gDW/h, yellow: between 0.5 and 1 mmol/gDW/h, orange: >1 mmol/gDW/h; for the product yields and rates we chose relative deviation as a criterion: dark green: data in predicted range, light green: deviation <5%, yellow: deviation between 5 and 10%, orange: deviation between 10 and 15%, red: deviation above 15%. Due to the small measured rated we used the absolute deviation as a criterion for the exchange rates.

|  | Dilution  rate[h^-1^] | Exchange rates [mmol/gDW_c_/h] | | | | | | | | | Product yields and ratios [mol/mol] | | | | | |
| --- | --- | --- | --- | --- | --- | --- | --- | --- | --- | --- | --- | --- | --- | --- | --- | --- |
|  |  | Ethanol | | | CO_2_ | | | Methane | | | CH_4_:CO_2_ | | | CH_4_:Ethanol | | |
|  |  | Sim1 | Sim2 | Exp. | Sim1 | Sim2 | Exp. | Sim1 | Sim2 | Exp. | Sim1 | Sim2 | Exp. | Sim1 | Sim2 | Exp. |
| Reactor 1 | 0.00054 | 1.27 | 0.63 | 0.43 | 0.62 | 0.31 | 0.17 | 1.89 | 0.94 | 0.59 | 3.03 | 3.07 | 3.56 | 1.49 | 1.48 | 1.38 |
|  | 0.00060 | 1.27 | 0.64 | 0.49 | 0.62 | 0.31 | 0.19 | 1.89 | 0.94 | 0.67 | 3.04 | 3.08 | 3.50 | 1.49 | 1.48 | 1.39 |
|  | 0.00072 | 1.28 | 0.66 | 0.58 | 0.63 | 0.31 | 0.22 | 1.90 | 0.95 | 0.75 | 3.05 | 3.09 | 3.47 | 1.49 | 1.47 | 1.29 |
|  | 0.00085 | 1.29 | 0.65 | 0.67 | 0.63 | 0.31 | 0.30 | 1.92 | 0.96 | 0.99 | 3.05 | 3.11 | 3.31 | 1.48 | 1.47 | 1.47 |
|  | 0.0010 | 1.30 | 0.66 | 0.42 | 0.63 | 0.31 | 0.19 | 1.93 | 0.97 | 0.61 | 3.06 | 3.13 | 3.25 | 1.48 | 1.46 | 1.47 |
|  | 0.0012 | 1.31 | 0.67 | 0.56 | 0.63 | 0.31 | 0.25 | 1.94 | 0.98 | 0.86 | 3.07 | 3.15 | 3.44 | 1.48 | 1.46 | 1.52 |
|  | 0.0017 | 1.36 | 0.71 | 0.66 | 0.64 | 0.32 | 0.29 | 1.99 | 1.02 | 0.93 | 3.11 | 3.22 | 3.17 | 1.47 | 1.44 | 1.42 |
| Reactor 2 | 0.00053 | 1.27 | 0.63 | 0.57 | 0.62 | 0.31 | 0.22 | 1.89 | 0.94 | 0.76 | 3.03 | 3.07 | 3.52 | 1.49 | 1.48 | 1.34 |
|  | 0.00058 | 1.27 | 0.64 | 0.50 | 0.62 | 0.31 | 0.19 | 1.89 | 0.94 | 0.65 | 3.04 | 3.08 | 3.46 | 1.49 | 1.48 | 1.32 |
|  | 0.00071 | 1.28 | 0.64 | 0.67 | 0.63 | 0.31 | 0.26 | 1.90 | 0.95 | 0.88 | 3.04 | 3.09 | 3.37 | 1.49 | 1.47 | 1.32 |
|  | 0.00083 | 1.29 | 0.65 | 0.78 | 0.63 | 0.30 | 0.34 | 1.91 | 0.96 | 1.12 | 3.05 | 3.11 | 3.27 | 1.49 | 1.47 | 1.43 |
|  | 0.0010 | 1.30 | 0.66 | 0.75 | 0.63 | 0.31 | 0.34 | 1.93 | 0.97 | 1.12 | 3.06 | 3.13 | 3.25 | 1.48 | 1.46 | 1.48 |
|  | 0.0012 | 1.32 | 0.68 | 0.64 | 0.63 | 0.31 | 0.27 | 1.95 | 0.99 | 0.93 | 3.08 | 3.16 | 3.46 | 1.48 | 1.46 | 1.45 |

References

1. Heyer R, Kohrs F, Benndorf D, Rapp E, Kausmann R, Heiermann M, et al. Metaproteome analysis of the microbial communities in agricultural biogas plants. N. Biotechnol. 2013; 30: 614–622.

2. Kohrs F, Heyer R, Bissinger T, Kottler R, Schallert K, Püttker S, et al. Proteotyping of laboratory-scale biogas plants reveals multiple steady-states in community composition. Anaerobe. 2017. doi: 10.1016/j.anaerobe.2017.02.005.

3. Muth T, Behne A, Heyer R, Kohrs F, Benndorf D, Hoffmann M, et al. The MetaProteomeAnalyzer. A powerful open-source software suite for metaproteomics data analysis and interpretation. J Proteome Res. 2015; 14: 1557–1565. doi: 10.1021/pr501246w.

4. Craig R, Beavis RC. TANDEM. Matching proteins with tandem mass spectra. Bioinformatics. 2004; 20: 1466–1467. doi: 10.1093/bioinformatics/bth092.

5. Geer LY, Markey SP, Kowalak JA, Wagner L, Xu M, Maynard DM, et al. Open mass spectrometry search algorithm. J Proteome Res. 2004; 3: 958–964. doi: 10.1021/pr0499491.

6. Shevchenko A, Sunyaev S, Loboda A, Shevchenko A, Bork P, Ens W, et al. Charting the Proteomes of Organisms with Unsequenced Genomes by MALDI-Quadrupole Time-of-Flight Mass Spectrometry and BLAST Homology Searching. Anal. Chem. 2001; 73: 1917–1926. doi: 10.1021/ac0013709.

7. Shida O, Takagi H, Kadowaki K, Komagata K. Proposal for two new genera, Brevibacillus gen. nov. and Aneurinibacillus gen. nov. Int. J. Syst. Bacteriol. 1996; 46: 939–946. doi: 10.1099/00207713-46-4-939.

8. Spencer RC. Bacillus anthracis. J. Clin. Pathol. 2003; 56: 182–187. doi: 10.1136/jcp.56.3.182.

9. Heyer R, Benndorf D, Kohrs F, Vrieze J de, Boon N, Hoffmann M, et al. Proteotyping of biogas plant microbiomes separates biogas plants according to process temperature and reactor type. Biotechnol Biofuels. 2016; 9: 155. doi: 10.1186/s13068-016-0572-4.

10. Nakano MM, Zuber P. Anaerobic growth of a "strict aerobe" (Bacillus subtilis). Annu. Rev. Microbiol. 1998; 52: 165–190. doi: 10.1146/annurev.micro.52.1.165.

11. Marino M, Ramos HC, Hoffmann T, Glaser P, Jahn D. Modulation of anaerobic energy metabolism of Bacillus subtilis by arfM (ywiD). J. Bacteriol. 2001; 183: 6815–6821. doi: 10.1128/JB.183.23.6815-6821.2001.

12. Bairoch A, Bougueleret L, Altairac S, Amendolia V, Auchincloss A, Argoud-Puy G, et al. The Universal Protein Resource (UniProt) 2009. Nucleic Acids Res. 2009; 37: 74.

13. Müller B, Sun L, Schnurer A. First insights into the syntrophic acetate-oxidizing bacteria--a genetic study. Microbiologyopen. 2013; 2: 35–53. doi: 10.1002/mbo3.50.
